# Low-Valent-Tungsten-Catalyzed Aerobic Oxidative Cross-Dehydrogenative Coupling Reaction

**DOI:** 10.3390/molecules28248071

**Published:** 2023-12-13

**Authors:** Chunsheng Li, Yaoyang Chen, Feihua Ye, Junhua Chen, Jia Zheng

**Affiliations:** 1 School of Environmental and Chemical Engineering, Zhaoqing University, Zhaoqing 526061, China; cyy1483211575@163.com (Y.C.); yefeihua@zqu.edu.cn (F.Y.);; 2 The Marine Biomedical Research Institute, Guangdong Medical University, Zhanjiang 524023, China

**Keywords:** cross-dehydrogenative coupling, aerobic, thiophosphates, 3-sulfenylated indoles

## Abstract

A straightforward and convenient protocol was established for the synthesis of thiophosphates and 3-sulfenylated indoles via low-valent-tungsten-catalyzed aerobic oxidative cross-dehydrogenative coupling reactions. These reactions occur under mild conditions and simple operations with commercially available starting materials, processing the advantage of excellent atom and step economy, broad substrate scope, and good functional groups tolerance. Moreover, this transformation could be practiced on the gram scale, which exhibits great potential in the preparation of drug-derived or bioactive molecules.

## 1. Introduction

**Figure 1:**
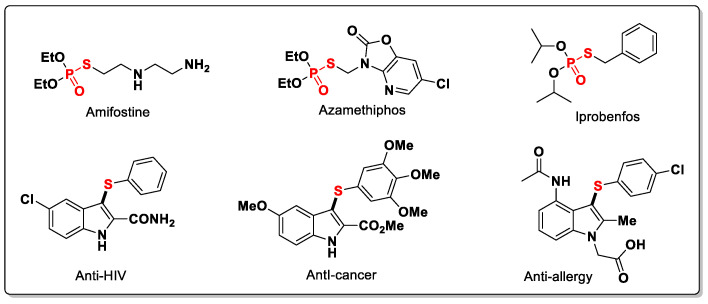
Representative biologically active sulfur-containing molecules.

Organosulfur molecules are an essential constituent of modern biological and pharmaceutical chemistry [1,2]. So far, a large portion of marketed drugs, which contain a thioethers structural motif, display incomparable anticancer, antibacterial, high blood pressure, diabetes, anti-inflammatory, anti-HIV, and so on, bioactivity (Figure 1) [3,4]. Moreover, organosulfur compounds also exhibit excellent mechanical properties in the field of materials science [5,6]. Therefore, much attention has been devoted to the construction of these sulfur-containing privileged structural fragments.

**Scheme 1:**
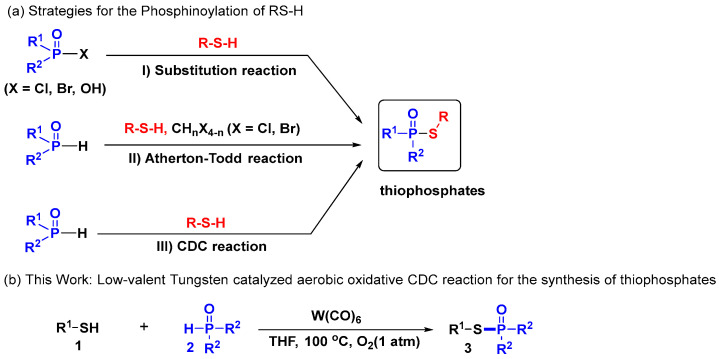
Synthetic routes of thiophosphates.

Due to the diverse remarkable chemical and biological properties of sulfur-containing motifs (such as thiophosphates and 3-sulfenylated indoles), these molecules featured broad utility in the field of pharmaceutical chemistry, organic synthesis, and materials science. Accordingly, several synthetic routes for the construction of thiophosphates have been well established in recent years (Scheme 1) [7,8,9]. These methods could be divided into four categories: (a) direct nucleophilic substitution of pre-functionalized S-X or P(O)-X reagents; [10,11,12] (b) the Atherton–Todd reaction with the utilization of polyhaloalkanes as both the reagent and solvent; [13,14,15,16] and (c) cross-dehydrogenative couplings of P-H bond and S-H bond [17,18,19,20,21,22,23]. Among these methods, the direct phosphorylation of thiols is atom- and step-economic, practical, and environmentally friendly. Recently, Han’s group reported direct Pd-catalyzed dehydrogenative cross-coupling reactions of the readily available P(O)-H molecules with thiols for the construction of phosphorothioates in excellent yields [17]. Subsequently, Wu and coworkers successfully applied a visible light photoredox catalytic system for the direct phosphorylation of thiols with excellent substrate scope [23]. Despite tremendous progress having been achieved, due to P-H and S-H being easily oxidized by stoichiometric oxidants, the transition-metal-catalyzed aerobic oxidative cross-dehydrogenative coupling of thiols with readily available P(O)-H substrates is still hard to achieve.

On the other hand, 3-arylthioindoles occur abundantly in biologically active compounds (Figure 1) [24,25]. Therefore, considerable methods, such as transition metal catalysis [26,27], hyper-valent iodine catalysis [28], organo-catalysis [29], photocatalysis [30,31,32], electrocatalysis [33], etc., have been exploited for the preparation of 3-arylthioindoles. For example, Liu and coworkers reported a visible-light-induced, graphene-oxide-promoted C3-chalcogenylation of indoles for the synthesis of 3-sulfenylindoles and 3-selenylindoles with the advantages of high yields and a simple operation. However, some of these works suffer from certain drawbacks like harsh reaction conditions, pre-functionalized reagents, or potentially dangerous oxidants. Therefore, the development of more optional coupling reactions for the synthesis of 3-arylthioindoles is in high demand. Moreover, Song’s group revealed that the transition metal tungsten catalytic system could realize the oxidative dehydrogenative coupling of anilines for the synthesis of azoaromatics and azoxyaromatics [34]. Herein, considering the significance of organosulfur molecules and our continuous interest in aerobic cross-dehydrogenative coupling reactions for the construction of organosulfur compounds [35,36], we disclosed a tungsten-catalyzed aerobic oxidative cross-dehydrogenative coupling of thiols and phosphonates or indoles for the facile synthesis of organosulfur derivatives after exerting tremendous efforts.

## 2. Results and Discussion

In order to verify the feasibility of our hypothesis, we selected 4-methylbenzenethiol (**1a**) and diethyl phosphite (**2a**) as the model substrates to investigate the optimal reaction conditions of this transformation as in Table 1. To our delight, when this reaction was conducted in the presence of tungsten catalysts, such as W(CO)_6_, W(CH_3_CN)_3_(CO)_3_, and W(COD)_2_(CO)_4_ in MeCN at 80 °C, the product **3a** was indeed generated as expected (entries 1–3). For the purpose of increasing the efficiency of this reaction, various solvents (such as MeCN, DMSO, DMF, DMA, DCE, toluene, dioxane, and THF) were screened under identical conditions; these experiments revealed that THF was the optimal choice among other solvents (entries 4–10). The yield of product **3a** could be increased to 64%. Further optimization was focused on the examination of different types of oxidants, such as DDQ, NQ, TBHP, air, and O_2_. Fortunately, the utilization of quinone oxidants delivered higher yields of **3a** (entries 10–12, respectively). Notably, when air or O_2_ was examined, comparable results were achieved (entries 15–16). Subsequently, increasing the temperature to 100 °C, the desired product **3a** could be obtained in 90% yield (entry 18). Finally, controlled experiments indicated that tungsten catalysis and an oxidant were essential for this reaction (entries 20–21).

**Scheme 2:**
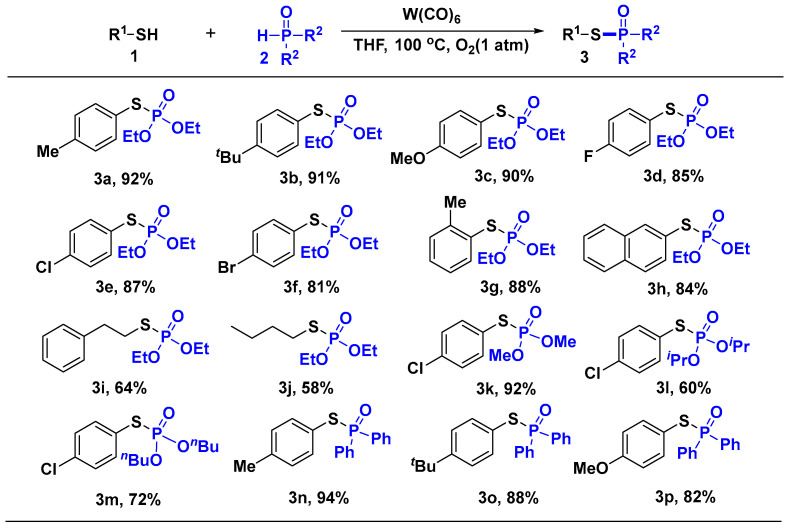
Substrate scope of the aerobic dehydrogenative coupling of thiols with phosphonate. thiols **1** (0.2 mmol), phosphonate **2** (0.3 mmol), O_2_ (1 atm), W(CO)_6_ (10 mol%), THF (2 mL), 100 °C, 24 h.

With the optimal reaction conditions established, we turned to explore the scope of thiols and phosphonates or diarylphosphine oxides. Firstly, substituted thiophenols were evaluated in this transformation, as shown in Scheme 2 (see Supplementary Materials for details). Delightfully, various aryl thiols could participate very well in this reaction to deliver the corresponding products in good yields (**3a**–**3k**). The electron-donating groups methyl (**3a**) and methoxy (**3b**) and electron- withdrawing substituents halogens were all compatible in this reaction (**3d**–**3f**). The halogen substituents are undoubtedly important for the further structural modification of thiophosphates. Moreover, aliphatic thiols such as 2-phenylethane-1-thiol(**1i**) and butane-1-thiol(**1j**) were well tolerated in this reaction system and converted into the products in good yields (**3i**–**3j**). In addition, except for these thiol substrates, several phosphonates were tested. Likewise, the substituents had no significant effect on the yield of products (**3l**–**3m**). Moreover, the diarylphosphine oxides only led to a slight decrease in the reaction yields (**3n**–**3p**).

**Scheme 3:**
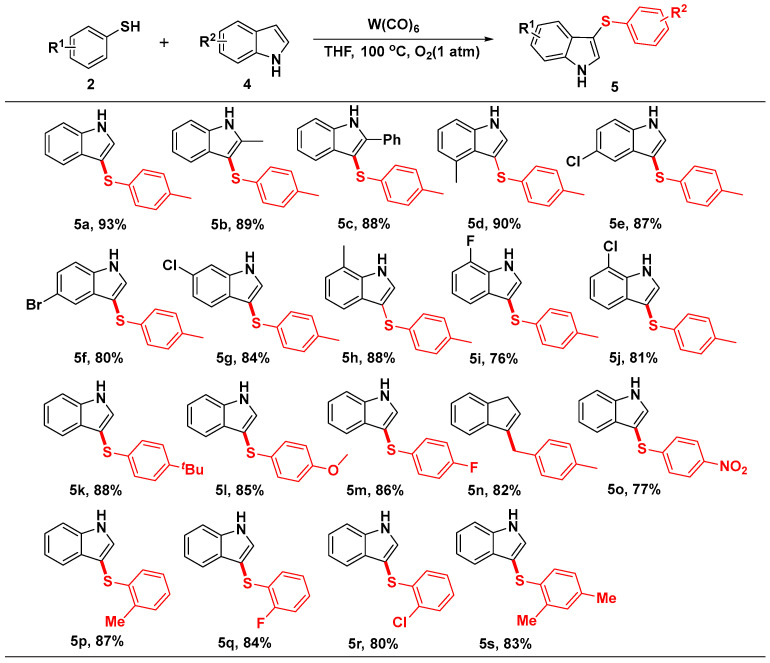
Substrate scope of the aerobic dehydrogenative coupling of thiols with indoles. Thiols **1** (0.2 mmol), indoles **4** (0.3 mmol), O_2_ (1 atm), W(CO)_6_ (10 mol%), THF (2 mL), 100 °C, 24 h.

According to the above experiment results, we supposed that the electron-rich substrates are more suitable in this process. Therefore, the recognized electron-rich substrates, like indoles, were investigated subsequently. Pleasingly, indole could convert into the 3-sulfenylated indoles in good yields. Consequently, different indoles were examined without further conditions optimization. The corresponding results are summarized in Scheme 3. The substituents in the different positions of the indole ring were examined affording the corresponding products in moderate to good yields smoothly (**5a**–**5d**). As expected, the introduction of electron-donating groups indeed increased the yields of the reaction (**5c**–**5d**). Additionally, the halogen-substituted indole substrates were well tolerated in this reaction, which could allow for the further structural modification of functionalized 3-sulfenylated indoles. The substrates with electron-donating (alkyl groups, methoxy) and electron-withdrawing substituents on the specific positions of the ring had no obvious influence, providing the desired 3-sulfenylated indoles successfully (**5g**–**5j**). On the other hand, a wide range of aryl thiols efficiently underwent the aerobic oxidative cross-dehydrogenative coupling reaction, leading to the formation of various 3-sulfenylated indoles with good to excellent yields. The result indicated that the position of the substituents only had a slight effect on the efficiency of this transformation (**5k**–**5s**).

**Scheme 4:**
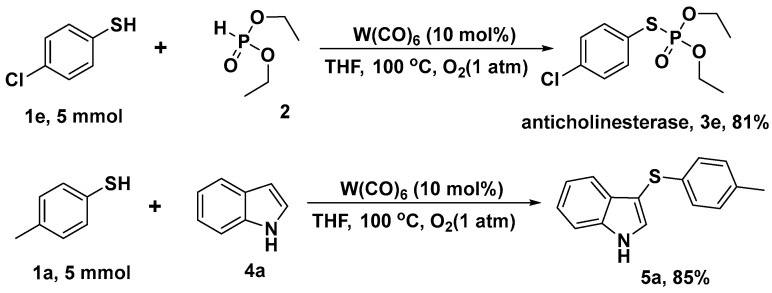
Large-scale preparation.

To further evaluate the practicality of this reaction, a gram-scale reaction was conducted under optimal conditions (Scheme 4). The bioactive molecules **3e** could be obtained in high yields, which confirmed the potential application of this protocol. Moreover, product **5a** could also be synthesized at the gram scale under optimal reaction conditions.

**Scheme 5:**
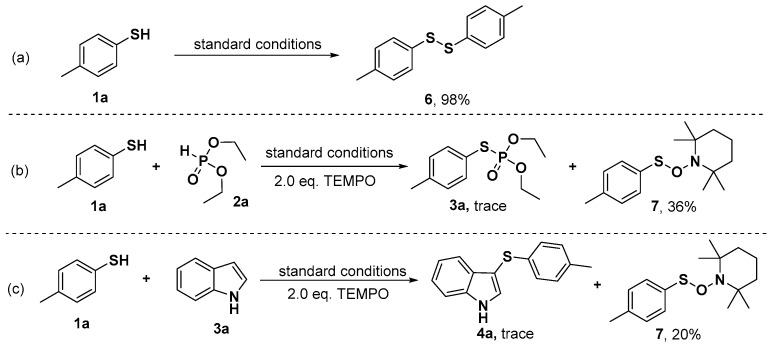
Controlled experiments.

To elucidate the mechanism of this reaction, several controlled experiments were performed. Under the standard conditions without the addition of phosphonates or indoles, disulfide (**6**) was observed as the only product in a high yield (Scheme 5a). Subsequently, when phosphonates (**2a**) and 4-methylbenzenethiol (**2a**) were reacted in the presence of radical scavenger TEMPO, this reaction process was inhibited, and the expected byproduct **7** was detected in low yields (Scheme 5b). The radical trapping adducts were obtained in 36% yield. Likewise, the reaction of indole with 4-methylbenzenethiol (**2a**) could also be inhibited by the radical scavenger TEMPO, revealing that the radical pathway was involved in these two reactions (Scheme 5c).

**Scheme 6:**
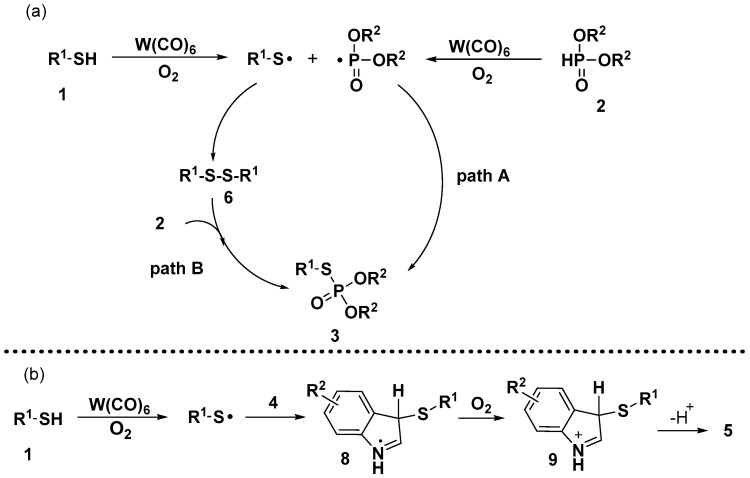
Plausible mechanistic pathway.

Based on the mechanistic experiments and previous reports [34], the plausible mechanism of this aerobic oxidative cross-dehydrogenative coupling reaction is proposed in Scheme 6. Firstly, as shown in Scheme 6a, the thiol converts into the thiyl radical species, which underwent the homocoupling reaction to generate the disulfide product **6**. On the other hand, the P-centered radical was formed from phosphonates **2** through a similar aerobic oxidative process. Finally, the P-centered radical coupled with the thiyl radical to deliver the desired product **3**. (path A), or the disulfide product **6** was coupled with the starting materials **2** to generate the corresponding product **3** (path B). On the other hand, the thiyl radical species were generated under the O_2_ atmosphere. Subsequently, the thiyl radical species could react with **4** to give the radical intermediate **8**. Next, the intermediate **9** was formed via the oxidation of the radical intermediate **8**. Finally, the deprotonation of the intermediate **9** led to the generation of 3-sulfenylated indole products.

## 3. Materials and Methods

### 3.1. Materials

All reagents were obtained from commercial sources and used directly without further purification unless otherwise noted. ^1^H NMR and ^13^C NMR spectra were recorded on Bruker AVANCE III 400 MHz or 500 MHz. ^1^H NMR and ^13^C NMR chemical shifts were determined relative to internal standard TMS at δ 0.0. Chemical shifts (δ) are reported in ppm, and coupling constants (J) are in Hertz (Hz).

### 3.2. General Methods for the Preparation of Thiophosphates

A 25 mL dried Schlenk tube was added to the mixture of thiols **1** (0.20 mmol) and phosphonates **2** (0.30 mmol), W(CO)_6_ (0.02 mmol) in anhydrous THF (2.0 mL). The gas in the Schlenk tube was replaced by O_2_ three times. The reaction was then allowed to stir at 100 °C for 24 h. Upon completion, the reaction mixture was washed by saturated NaCl aqueous solution (2 × 10 mL) and then extracted with ethyl acetate (2 × 10 mL), and the organic layers were combined, dried over anhydrous MgSO_4_, filtered, and concentrated under reduced pressure. The residue was separated by column chromatography (petroleum ether/ethyl acetate) to give the pure thiophosphates.

### 3.3. General Methods for the Preparation of Thiophosphates

A 25 mL dried Schlenk tube was added to the mixture of thiols 1 (0.20 mmol), indoles 4 (0.30 mmol), and W(CO)_6_ (0.02 mmol) in anhydrous THF (2.0 mL). The gas in the Schlenk tube was replaced by O_2_ three times. The reaction was then allowed to stir at 100 °C for 24 h. Upon completion, the reaction mixture was washed by saturated NaCl aqueous solution (2 × 10 mL) and then extracted with ethyl acetate (2 × 10 mL), and the organic layers were combined, dried over anhydrous MgSO_4_, filtered, and concentrated under reduced pressure. The residue was separated by column chromatography (petroleum ether/ethyl acetate) to give the pure 3-sulfenylated indoles.

## 4. Conclusions

In summary, we have disclosed an efficient tungsten-catalyzed aerobic oxidative cross-dehydrogenative coupling reaction of thiols and phosphonates or indoles. Environmentally friendly molecular oxygen is the sole oxidant in this transformation. Furthermore, readily available reagents, mild reaction conditions, broad substrates scope, and good functional group tolerance makes this approach applicable in the facile synthesis and modification of bioactive molecules.

## Figures and Tables

**Table 1:** Optimization of the reaction conditions ^a^.

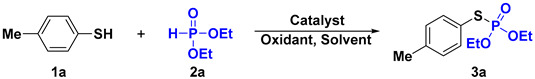				
Entry	Catalyst	Oxidant	Solvent	Yield (%) ^b^
1	W(CO)_6_	BQ	MeCN	29
2	W(CO)_3_(CH_3_CN)_3_	BQ	MeCN	21
3	W(COD)_2_(CO)_4_	BQ	MeCN	23
4	W(CO)_6_	BQ	DMSO	30
5	W(CO)_6_	BQ	DMF	trace
6	W(CO)_6_	BQ	DMA	trace
7	W(CO)_6_	BQ	DCE	32
8 ^c^	W(CO)_6_	BQ	Dioxane	trace
9	W(CO)_6_	BQ	Toluene	24
10	W(CO)_6_	BQ	THF	64
11	W(CO)_6_	DDQ	THF	72
12	W(CO)_6_	NQ	THF	61
13	W(CO)_6_	TBHP	THF	10
14	W(CO)_6_	K_2_S_2_O_8_	THF	16
15	W(CO)_6_	air	THF	70
16	W(CO)_6_	O_2_	THF	73
17	W(CO)_6_	O_2_	THF	50
18 ^d^	W(CO)_6_	O_2_	THF	90
19 ^e^	W(CO)_6_	O_2_	THF	85
20	-	O_2_	THF	10
21	W(CO)_6_	N_2_	THF	n.d.

^a^ 4-methylbenzenethiol **1a** (0.2 mmol), diethyl phosphonate **2a** (0.3 mmol), oxidant (0.4 mmol), catalyst (10 mol%), solvent (2 mL), 80 °C, 24 h; ^b^ yields were determined by GC-MS, n.d. = not detected; ^c^ the reaction temperature was 60 °C; ^d^ the reaction temperature was 100 °C; ^e^ the reaction temperature was 120 °C.

## Data Availability

Data are contained within the article and Supplementary Materials.

## Supplementary Materials

The following supporting information can be downloaded at: https://www.mdpi.com/article/10.3390/molecules28248071/s1, the NMR data and spectra of the catalytic products. Additional references cited within the Supporting Information [19,22,23,27,28,31,32,37,38,39].
