# Supplementary material for: Low-Valent-Tungsten-Catalyzed Aerobic Oxidative Cross-Dehydrogenative Coupling Reaction
Source: Molecules. 2023 Dec 13;28(24):8071. doi: 10.3390/molecules28248071 (PMC10745622; doi:10.3390/molecules28248071)
Supplement: Supplementary file 1 [file molecules-28-08071-s001.zip › molecules-2706661-supplementary.pdf]

Low-Valent-Tungsten-Catalyzed Aerobic Oxidative Cross-Dehydrogenative Coupling Reaction

Chunsheng Li [1,*], Yaoyang Chen [1], Feihua Ye [1], Junhua Chen [1] and Jia Zheng [2,*]

[1] School of Environmental and Chemical Engineering, Zhaoqing University, Zhaoqing 526061, China; cyy1483211575@163.com (Y.C.); yefeihua@zqu.edu.cn (F.Y.)
[2] The Marine Biomedical Research Institute, Guangdong Medical University, Zhanjiang 524023, China
* Correspondence: lichunsheng@zqu.edu.cn (C.L.); jiatiger@163.com (J.Z.)

Table of Contents

General information S3

General methods for the preparation of thiophosphates S4

General methods for the synthesis of 3-sulfenylated indoles S4

Characterization data for all products. S6

References. S12

NMR spectra for all the compounds S13

General Information

Melting points were measured using a melting point instrument and are uncorrected. ^1H and ^{13}C NMR spectra were recorded on a 400 MHz NMR spectrometer. The chemical shifts are referenced to signals at 7.24 and 77.0 ppm, respectively, and chloroform was used as a solvent with TMS as the internal standard. GC-MS data were obtained using electron ionization. TLC was performed using commercially available 100-400 mesh silica gel plates (GF254). Unless otherwise noted, purchased chemicals were used without further purification.

General Methods for the Preparation of Thiophosphates

A 25 mL dried Schlenk tube was added the mixture of thiols **1** (0.20 mmol), phosphonates **2** (0.30 mmol), $W(CO)_6$ (0.02 mmol) in anhydrous THF (2.0 mL). The gas in the Schlenk tube was replaced by O_2 for three times. The reaction was then allowed to stir at 100 °C for 24 h. Upon completion, the reaction mixture was washed by saturated NaCl aqueous solution (2×10 mL) and then extracted with ethyl acetate (2×10 mL), and the organic layers were combined, dried over anhydrous $MgSO_4$, filtered, and concentrated under reduced pressure. The residue was separated by column chromatography (petroleum ether/ethyl acetate) to give the pure thiophosphates.

General Methods for the Preparation of 3-sulfenylated Indoles

A 25 mL dried Schlenk tube was added the mixture of thiols **1** (0.20 mmol), indoles **4** (0.30 mmol), $W(CO)_6$ (0.02 mmol) in anhydrous THF (2.0 mL). The gas in the Schlenk tube was replaced by O_2 for three times. The reaction was then allowed to stir at 100 °C for 24 h. Upon completion, the reaction mixture was washed by saturated NaCl aqueous solution (2×10 mL) and then extracted with ethyl acetate (2×10 mL), and the organic layers were combined, dried over anhydrous $MgSO_4$, filtered, and concentrated under reduced pressure. The residue was separated by column chromatography (petroleum ether/ethyl acetate) to give the pure 3-sulfenylated indoles.

Characterization Data for All Products

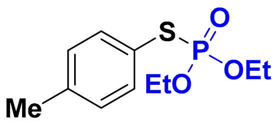

O,O-diethyl S-(*p*-tolyl) phosphorothioate (3a)[22] It was obtained after purification by column chromatography on silica gel (pe0troleum ether/ethyl acetate) as colorless oil (4 7.9 mg, 92%); ^{1}H NMR (400 MHz, Chloroform-d) δ 7.44 (d, J = 7.2 Hz, 2H), 7.15 (d, J = 7.2 Hz, 2H), 4.26 - 4.10 (m, 4H), 2.34 (s, 4H), 1.31 (t, J = 8.0 Hz, 6H); ^{13}C NMR (100 MHz, Chloroform-d) δ 139.2 (d, J = 3.0 Hz), 134.5 (d, J = 5.0 Hz), 130.07 (d, J = 2.3 Hz), 122.7 (d, J = 7.3 Hz), 63.9 (d, J = 6.2 Hz), 21.1, 15.9 (d, J = 7.2 Hz).

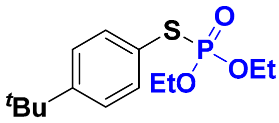

S-(4-(*tert*-butyl)phenyl) *O,O*-diethyl phosphorothioate (3b)[22] It was obtained after purification by column chromatography on silica gel (petroleum ether/ethyl acetate) as colorless oil (54.9 mg, 91%); ^{1}H NMR (400 MHz, Chloroform-d) δ 7.48 (d, J = 7.6 Hz, 2H), 7.36 (d, J = 7.6 Hz, 2H), 4.25 - 4.14 (m, 4H), 1.30 (s, 15H); ^{13}C NMR (100 MHz, Chloroform-d) δ 152.2 (d, J = 3.1 Hz), 134.2 (d, J = 5.1 Hz), 126.3 (d, J = 2.1 Hz), 122.7 (d, J = 7.2 Hz), 63.9 (d, J = 6.1 Hz), 34.5, 31.0, 15.9 (d, J = 7.2 Hz).

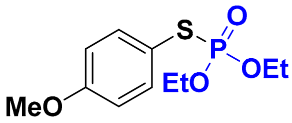

***O,O*-diethyl S-(4-methoxyphenyl) phosphorothioate (3c)**[22] It was obtained after purification by column chromatography on silica gel (petroleum ether/ethyl acetate) as yellow oil (49.7 mg, 90%); ^{1}H NMR (400 MHz, Chloroform-d) δ 7.47 (d, J = 7.8 Hz, 2H), 6.87 (d, J = 7.8 Hz, 2H), 4.23 - 4.12 (m, 4H), 3.79 (s, 3H), 1.31 (s, 6H); ^{13}C NMR (100 MHz, Chloroform-d) δ 160.3 (d, J = 3.0 Hz), 136.2 (d, J = 4.7 Hz), 116.3 (d, J = 7.4 Hz), 114.8 (d, J = 2.2 Hz), 63.8 (d, J = 6.3 Hz), 55.1, 15.8 (d, J = 7.1 Hz).

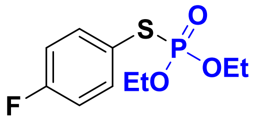

O,O-diethyl S-(4-fluorophenyl) phosphorothioate (3d)[22] It was obtained after purification by column chromatography on silica gel (petroleum ether/ethyl acetate) as colorless oil (44.9 mg, 85 %); ^{1}H NMR (400 MHz, Chloroform-d) δ 7.55 (d, J = 7.8 Hz, 2H), 7.05 (d, J = 7.8 Hz, 2H), 4.25 - 4.13 (m, 4H), 1.32 (s, 6H); ^{13}C NMR (100 MHz, Chloroform-d) δ 163.24 (dd, J = 250.0, 3.2 Hz), 136.60 (dd, J = 8.4, 5.0 Hz), 121.57 (dd, J = 7.3, 3.4 Hz), 116.46 (dd, J = 22.2, 2.1 Hz), 64.11 (d, J = 6.4 Hz), 15.91 (d, J = 7.1 Hz).

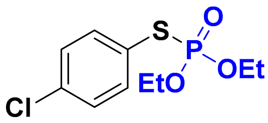

S-(4-chlorophenyl) O,O-diethyl phosphorothioate (3e)[22] It was obtained after purification by column chromatography on silica gel (petroleum ether/ethyl acetate) as colorless oil (48.7 mg, 87 %); ^{1}H NMR (400 MHz, Chloroform-d) δ 7.50 (d, J = 6.8 Hz, 2H), 7.33 (d, J = 6.8 Hz, 2H), 4.25 - 4.13 (m, 4H), 1.32 (s, 6H); ^{13}C NMR (100 MHz, Chloroform-d) δ 135.7 (d, J = 5.2 Hz), 135.5 (d, J = 3.3 Hz), 129.5 (d, J = 1.9 Hz), 125.1 (d, J = 7.3 Hz), 64.2 (d, J = 6.4 Hz), 16.0 (d, J = 7.1 Hz).

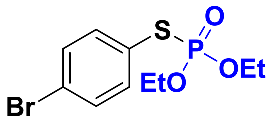

S-(4-bromophenyl) O,O-diethyl phosphorothioate (3f)[22] It was obtained after purification by column chromatography on silica gel (petroleum ether/ethyl acetate) as yellow oil (52.5 mg, 81 %); ^{1}H NMR (400 MHz, Chloroform-d) δ 7.48 (d, J = 7.8 Hz, 2H), 7.43 (d, J = 7.8 Hz, 2H), 4.24 - 4.13 (m, 4H), 1.32 (t, J = 7.2 Hz, 6H); ^{13}C NMR (100 MHz, Chloroform-d) δ 135.9 (d, J = 5.2 Hz), 132.4 (d, J = 2.0 Hz), 125.7 (d, J = 7.2 Hz), 123.6 (d, J = 3.5 Hz), 64.2 (d, J = 6.3 Hz), 16.0 (d, J = 7.0 Hz).

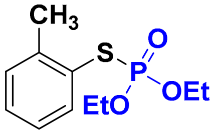

O,O-diethyl S-(*o*-tolyl) phosphorothioate (3g)[37] It was obtained after purification by column chromatography on silica gel (petroleum ether/ethyl acetate) as colorless oil (45.8 mg, 88 %); ^{1}H NMR (400 MHz, Chloroform-d) δ 7.61 (d, J = 6.0 Hz, 1H), 7.26 (s, 2H), 7.18 (s, 1H), 4.21 - 4.10 (m, 4H), 2.52 (s, 3H), 1.29 (t, J = 7.2 Hz, 6H); ^{13}C NMR (100 MHz, Chloroform-d) δ 142.1 (d, J = 5.5 Hz), 136.1 (d, J

= 4.2 Hz), 130.7 (d, *J* = 2.4 Hz), 129.3 (d, *J* = 2.9 Hz), 126.6 (d, *J* = 2.9 Hz), 125.6 (d, *J* = 7.3 Hz), 64.1 (d, *J* = 6.7 Hz), 21.3, 15.9 (d, *J* = 7.1 Hz).

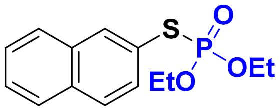

O,O-diethyl S-(naphthalen-2-yl) phosphorothioate (**3h**)[22] It was obtained after purification by column chromatography on silica gel (petroleum ether/ethyl acetate) as yellow oil (49.7 mg, 84 %); ^{1}H NMR (500 MHz, Chloroform-d) δ 8.09 (s, 1H), 7.84 - 7.81 (m, 3H), 7.62 - 7.60 (m, 1H), 7.52 - 7.50 (m, 2H), 4.26 - 4.17 (m, 4H), 1.31 (t, *J* = 7.2 Hz, 6H); ^{13}C NMR (125 MHz, Chloroform-d) δ 134.3 (d, *J* = 6.8 Hz), 133.6 (d, *J* = 2.3 Hz), 133.0 (d, *J* = 1.9 Hz), 130.9 (d, *J* = 4.1 Hz), 129.0 (d, *J* = 1.6 Hz), 127.67 (d, *J* = 4.7 Hz), 127.0 (d, *J* = 1.6 Hz), 126.7 (d, *J* = 1.6 Hz), 123.7 (d, *J* = 7.5 Hz), 64.1 (d, *J* = 6.3 Hz), 16.0 (d, *J* = 7.2 Hz).

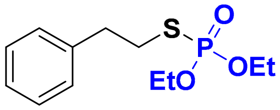

O,O-diethyl S-phenethyl phosphorothioate (**3i**)[18] It was obtained after purification by column chromatography on silica gel (petroleum ether/ethyl acetate) as colorless oil (35.1 mg, 64 %); ^{1}H NMR (400 MHz, Chloroform-d) δ 7.30 (s, 2H), 7.22 (s, 3H), 4.17 - 4.07 (m, 4H), 3.11 - 3.04 (m, 2H), 3.00 - 2.99 (m, 2H), 1.35 (t, *J* = 8.0 Hz, 6H); ^{13}C NMR (100 MHz, Chloroform-d) δ 139.3, 128.5, 128.4, 126.6, 63.4 (d, *J* = 6.0 Hz), 37.1 (d, *J* = 5.5 Hz), 31.9 (d, *J* = 3.8 Hz), 16.0 (d, *J* = 7.3 Hz).

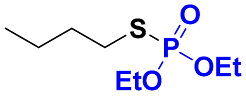

S-butyl O,O-diethyl phosphorothioate (**3j**)[23] It was obtained after purification by column chromatography on silica gel (petroleum ether/ethyl acetate) as yellow oil (26.2 mg, 58 %); ^{1}H NMR (500 MHz, Chloroform-d) δ 4.23 - 4.10 (m, 4H), 2.84 (dt, *J* = 14.4, 7.4 Hz, 2H), 1.67 (p, *J* = 7.4 Hz, 2H), 1.43 (dq, *J* = 14.8, 7.4 Hz, 2H), 1.37 (t, *J* = 7.1 Hz, 6H), 0.93 (t, *J* = 7.4 Hz, 3H); ^{13}C NMR (125 MHz, Chloroform-d) δ 63.4 (d, *J* = 5.9 Hz), 32.8 (d, *J* = 5.9 Hz), 30.6 (d, *J* = 4.0 Hz), 21.6, 16.0 (d, *J* = 7.3 Hz), 13.4.

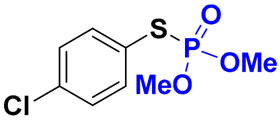

S-(4-chlorophenyl) O,O-dimethyl phosphorothioate (3k)[22] It was obtained after purification by column chromatography on silica gel (petroleum ether/ethyl acetate) as yellow oil (46.4 mg, 92 %); ^{1}H NMR (400 MHz, Chloroform-d) δ 7.50 (d, J = 8.0 Hz, 2H), 7.33 (d, J = 8.0 Hz, 2H), 3.84 (s, 3H), 3.81 (s, 3H); ^{13}C NMR (100 MHz, Chloroform-d) δ 135.8 (d, J = 5.2 Hz), 135.7 (d, J = 3.4 Hz), 129.7 (d, J = 2.1 Hz), 124.5 (d, J = 7.3 Hz), 54.4 (d, J = 6.2 Hz).

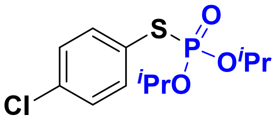

S-(4-chlorophenyl) O,O-di-*iso*propyl phosphorothioate (3l)[22] It was obtained after purification by column chromatography on silica gel (petroleum ether/ethyl acetate) as yellow oil (41.9 mg, 68 %); ^{1}H NMR (400 MHz, Chloroform-d) δ 7.54 (d, J = 7.2 Hz, 2H), 7.31 (d, J = 7.2Hz, 2H), 4.81 - 4.72 (m, 2H), 1.34 (s, 6H), 1.28 (s, 6H); ^{13}C NMR (100 MHz, Chloroform-d) δ 135.4 (d, J = 5.4 Hz), 135.0 (d, J = 3.1 Hz), 129.3 (d, J = 2.1 Hz), 125.8 (d, J = 7.1 Hz), 73.5 (d, J = 6.9 Hz), 23.7 (d, J = 4.2 Hz), 23.4 (d, J = 5.6 Hz).

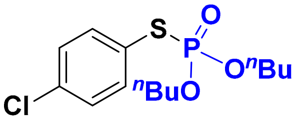

O,O-dibutyl S-(4-chlorophenyl) phosphorothioate (3m)[22] It was obtained after purification by column chromatography on silica gel (petroleum ether/ethyl acetate) as yellow oil (48.4 mg, 72 %); ^{1}H NMR (400 MHz, Chloroform-d) δ 7.50 (d, J = 8.0 Hz, 1H), 7.32 (d, J = 8.0 Hz, 1H), 4.14 - 4.09 (m, 4H), 1.64 (s, 4H), 1.37 (m, 4H), 0.91 (s, 6H); ^{13}C NMR (100 MHz, Chloroform-d) δ 135.6 (d, J = 5.2 Hz), 135.4 (d, J = 3.3 Hz), 129.4 (d, J = 1.9 Hz), 125.1 (d, J = 7.1 Hz), 67.9 (d, J = 6.8 Hz), 32.0 (d, J = 7.0 Hz), 18.6, 13.5.

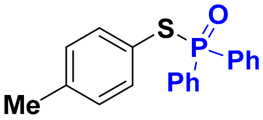

S-(*p*-tolyl) diphenylphosphinothioate (3n)[23] It was obtained after purification by column chromatography on silica gel (petroleum ether/ethyl acetate) as yellow oil (60.9 mg, 94 %); ^{1}H NMR (400 MHz, Chloroform-d) δ 7.84 (s, 4H), 7.42 (s, 6H), 7.31 (s, 2H), 7.00 (s, 2H), 2.23 (s, 3H); ^{13}C NMR (100

MHz, Chloroform-d) δ 139.2 (d, J = 2.1 Hz), 135.4 (d, J = 3.7 Hz), 132.7 (d, J = 106.8 Hz), 132.3 (d, J = 2.8 Hz), 131.7 (d, J = 10.2 Hz), 129.8 , 128.5 (d, J = 13.1 Hz), 122.3 (d, J = 5.2 Hz), 21.2.

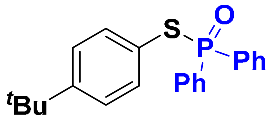

S-(4-(*tert*-butyl)phenyl) diphenylphosphinothioate (**3o**)[23] It was obtained after purification by column chromatography on silica gel (petroleum ether/ethyl acetate) as yellow oil (64.4 mg, 88 %); ^{1}H NMR (400 MHz, Chloroform-d) δ 7.86 - 7.82 (m, 4H), 7.48 - 7.42 (m, 6H), 7.35 (d, J = 6.3 Hz, 2H), 7.21 (d, J = 7.4 Hz, 2H), 1.23 (s, 9H); ^{13}C NMR (100MHz, Chloroform-d) δ 152.2 (d, J = 2.2 Hz), 135.1 (d, J = 3.7 Hz), 132.6 (d, J = 106.6 Hz) 132.1 (d, J = 2.8 Hz), 131.5 (d, J = 10.2 Hz), 128.4 (d, J = 13.1 Hz), 126.2, 122.2 (d, J = 5.2 Hz), 34.5, 31.0.

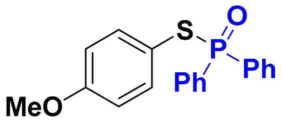

S-(4-methoxyphenyl) diphenylphosphinothioate (**3p**)[38] It was obtained after purification by column chromatography on silica gel (petroleum ether/ethyl acetate) as yellow oil (55.8 mg, 82 %); ^{1}H NMR (400 MHz, Chloroform-d) δ 7.86 - 7.82 (m, 4H), 7.49 - 7.43 (m, 6H), 7.33 (d, J = 6.4 Hz, 2H), 6.72 (d, J = 7.2 Hz, 2H), 3.71 (s, 3H); ^{13}C NMR (100 MHz, Chloroform-d) δ 160.4 (d, J = 2.2 Hz), 137. 0 (d, J = 3.7 Hz), 132. 5(d, J = 106.2 Hz), 132.2 (d, J = 2.8 Hz), 131.5 (d, J = 10.2 Hz), 128.4 (d, J = 13.1 Hz), 115.9 (d, J = 5.3 Hz), 114.7, 55.2.

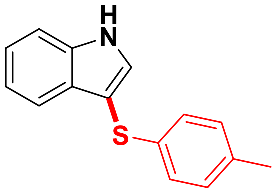

3-(*p*-Tolylthio)-1*H*-indole (**5a**)[28] It was obtained after purification by column chromatography on silica gel (petroleum ether/ethyl acetate) as white solid (M. p.=116-117 °C) (44.5 mg, 93%); ^{1}H NMR (400 MHz, Chloroform-*d*) δ 8.27 (s, 1H), 7.60 (d, J = 7.2 Hz, 1H), 7.37 (d, J = 10.4 Hz, 2H), 7.22 (d, J = 8.0 Hz, 1H), 7.15 (d, J = 8.0 Hz, 1H), 7.02 (d, J = 5.2 Hz, 2H), 6.96 (d, J = 5.2 Hz, 2H), 2.23 (s, 3H); ^{13}C NMR (100 MHz, Chloroform-*d*) δ 136.4, 135.4, 134.6, 130.4, 129.5, 129.1, 126.2, 122.9, 120.8, 119.6, 111.5, 103.3, 20.8.

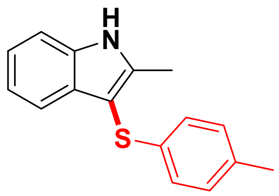

2-Methyl-3-(*p*-tolylthio)-1*H*-indole (5b)[28] It was obtained after purification by column chromatography on silica gel (petroleum ether/ethyl acetate) as white solid (M. p.=97-98 ºC) (45.1 mg, 89%); ^{1}H NMR (400 MHz, Chloroform-*d*) δ 7.95 (s, 1H), 7.45 (d, *J* = 8.0 Hz, 1H), 7.16 (d, *J* = 7.3 Hz, 1H), 7.07 - 7.01 (m, 2H), 6.85 (s, 4H), 2.32 (s, 3H), 2.12 (s, 3H); ^{13}C NMR (100 MHz, Chloroform-*d*) δ 140.9, 135.6, 135.3, 134.3, 130.2, 129.4, 125.7, 122.0, 120.6, 118.9, 110.6, 99.7, 20.8, 12.0.

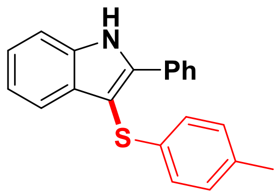

2-Phenyl-3-(*p*-tolylthio)-1*H*-indole (5c)[31] It was obtained after purification by column chromatography on silica gel (petroleum ether/ethyl acetate) as red oil (55.5 mg, 88%); ^{1}H NMR (400 MHz, Chloroform-*d*) δ 8.40 (s, 1H), 7.67 (s, 2H), 7.62 (d, *J* = 7.2 Hz, 1H), 7.34 - 7.32 (m, 4H), 7.21 (s, 1H), 7.13 (d, *J* = 6.8 Hz, 1H), 6.97 (s, 2H), 6.93 (s, 2H), 2.19 (s, 3H); ^{13}C NMR (100 MHz, Chloroform-*d*) δ 141.8, 135.7, 135.5, 134.3, 131.3, 131.1, 129.6, 128.6, 128.5, 128.1, 125.7, 111.1, 99.7, 20.8.

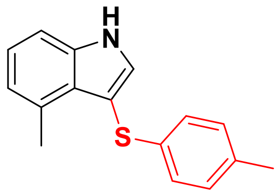

4-Methyl-3-(*p*-tolylthio)-1H-indole (5d)[32] It was obtained after purification by column chromatography on silica gel (petroleum ether/ethyl acetate) as a white solid (M.p.=138-139 ºC) (45.6 mg, 90 %); ^{1}H NMR (400 MHz, Chloroform-*d*) δ 8.33 (s, 1H), 7.38 (s, 1H), 7.22 (s, 1H), 7.13 (d, *J* = 7.2 Hz, 1H), 6.96 (s, 4H), 6.87 (s, 1H), 2.64 (s, 3H), 2.24 (s, 3H); ^{13}C NMR (100 MHz, Chloroform-*d*) δ 137.8, 137.0, 134.2, 132.1, 131.7, 129.5, 127.0, 125.4, 123.0, 122.4, 109.4, 102.8, 20.8, 18.6.

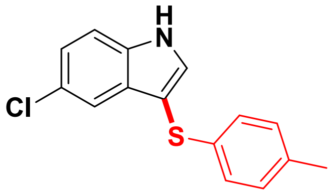

5-Chloro-3-(*p*-tolylthio)-1*H*-indole (5e)[28] It was obtained after purification by column chromatography on silica gel (petroleum ether/ethyl acetate) as white solid (M. p.=120-121 ºC) (47.5 mg, 87 %); ^{1}H NMR (400 MHz, Chloroform-d) δ 8.42 (s, 1H), 7.58 (s, 1H), 7.45 (s, 1H), 7.31 (d, J = 8.0 Hz, 1H), 7.19 (d, J = 8.0 Hz, 1H), 7.00 (s, 4H), 2.25 (s, 3H); ^{13}C NMR (100 MHz, Chloroform-d) δ 134.9, 134.9, 134.8, 131.7, 130.4, 129.6, 126.8, 126.3, 123.4, 119.1, 112.6, 103.5, 20.8.

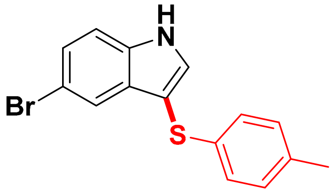

5-Bromo-3-(*p*-tolylthio)-1*H*-indole (5f)[28] It was obtained after purification by column chromatography on silica gel (petroleum ether/ethyl acetate) as white solid (M. p.=124-125 ºC) (50.7mg, 80 %); ^{1}H NMR (400 MHz, Chloroform-*d*) δ 8.38 (s, 1H), 7.74 (s, 1H), 7.40 (s, 1H), 7.30 (s, 1H), 7.24 (d, J = 8.0 Hz, 1H), 6.99 (s, 4H), 2.24 (s, 3H); ^{13}C NMR (100 MHz, Chloroform-*d*) δ 135.0, 135.0, 134.9, 131.6, 130.9, 129.6, 126.3, 126.0, 122.2, 114.3, 113.0, 103.3, 20.8.

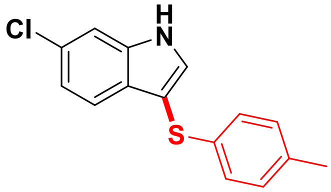

6-Chloro-3-(*p*-tolylthio)-1*H*-indole (5g)[28] It was obtained after purification by column chromatography on silica gel (petroleum ether/ethyl acetate) as white solid (M. p.=127-128 ºC) (45.9 mg, 84 %); ^{1}H NMR (400 MHz, Chloroform-*d*) δ 8.38 (s, 1H), 7.49 (d, J = 8.0 Hz, 1H), 7.44 (s, 1H), 7.40 (s, 1H), 7.11 (d, J = 8.0 Hz, 1H), 6.99 (s, 4H), 2.25 (s, 3H); ^{13}C NMR (100 MHz, Chloroform-*d*) δ 136.8, 134.9, 130.9, 129.5, 129.0, 127.7, 126.4, 121.6, 120.6, 111.5, 104.1, 20.8.

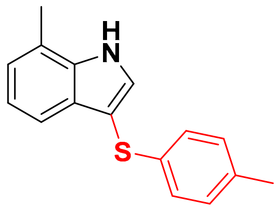

7-Methyl-3-(*p*-tolylthio)-1*H*-indole (5h)[39] It was obtained after purification by column chromatography on silica gel (petroleum ether/ethyl acetate) as white solid (M. p.=107 - 108 ºC) (44.5 mg, 88 %); ^{1}H NMR (400 MHz, Chloroform-*d*) δ 8.30 (s, 1H), 7.46 (s, 2H), 7.06 - 7.01 (m, 4H), 6.97 (s, 2H), 2.51 (s, 3H), 2.24 (s, 3H); ^{13}C NMR (101 MHz, Chloroform-*d*) δ 136.0, 135.5, 134.6, 130.1, 129.4, 128.7, 126.2, 123.5, 121.0, 120.7, 117.4, 103.9, 20.8, 16.4.

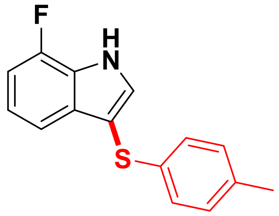

7-Fluoro-3-(*p*-tolylthio)-1*H*-indole (5i)[39] It was obtained after purification by column chromatography on silica gel (petroleum ether/ethyl acetate) as white solid (M.p.=94-96 ºC) (39.1 mg, 76 %); ^{1}H NMR (400 MHz, Chloroform-*d*) δ 8.58 (s, 1H), 7.47 (s, 1H), 7.36 (d, *J* = 8.0 Hz, 1H), 7.04 - 7.02 (m, 3H), 6.99 - 6.95 (m, 3H), 2.25 (s, 3H); ^{13}C NMR (101 MHz, Chloroform-*d*) δ 149.5 (d, *J* = 245.0 Hz), 134.9, 132.6 (d, *J* = 4.4 Hz), 130.9, 129.5, 129.2 (d, *J* = 123.9 Hz), 126.5, 124.8 (d, *J* = 13.3 Hz), 121.1 (d, *J* = 6.1 Hz), 115.4 (d, *J* = 3.5 Hz), 107.8 (d, *J* = 16.0 Hz), 104.8 (d, *J* = 2.5 Hz), 20.8.

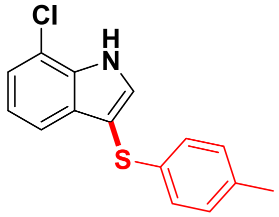

7-chloro-3-(*p*-tolylthio)-1H-indole (5j)[32] It was obtained after purification by column chromatography on silica gel (petroleum ether/ethyl acetate) as yellow oil (44.2 mg, 81 %); ^{1}H NMR (400 MHz, Chloroform-*d*) δ 8.61 (s, 1H), 7.50 (d, *J* = 8.0 Hz, 2H), 7.24 (d, *J* = 5.4 Hz, 1H), 7.08 (t, *J* = 7.2 Hz, 1H), 6.94 - 7.01 (m, 4H), 2.25 (s, 3H); ^{13}C NMR (100 MHz, Chloroform-d) δ 134.9, 134.9, 133.7, 130.8, 130.6, 129.5, 126.5, 122.3, 121.6, 118.4, 116.9, 105.1, 20.8.

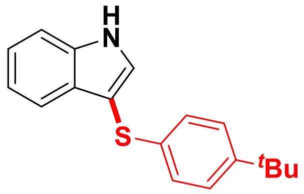

3-((4-(*tert*-Butyl)phenyl)thio)-1*H*-indole (5k)[28] It was obtained after purification by column chromatography on silica gel (petroleum ether/ethyl acetate) as white solid (M. p.=177 - 178 ºC) (49.5 mg, 88 %); ^{1}H NMR (400 MHz, Chloroform-*d*) δ 8.38 (s, 1H), 7.65 (d, *J* = 8.0 Hz, 1H), 7.46 - 7.41 (m, 2H), 7.25 (s, 1H), 7.18 (d, *J* = 8.0 Hz, 3H), 7.05 (d, *J* = 8.0 Hz, 2H), 1.24 (s, 9H); ^{13}C NMR (100 MHz, Chloroform-*d*) δ 147.9, 136.4, 135.7, 130.5, 129.3, 125.7, 123.0, 120.8, 119.7, 111.5, 103.4, 34.3, 31.3.

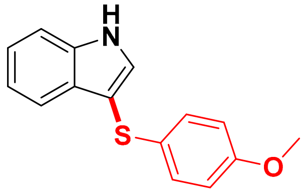

3-((4-Methoxyphenyl)thio)-1*H*-indole (5l)[28] It was obtained after purification by column chromatography on silica gel (petroleum ether/ethyl acetate) as white solid (M. p.=115 - 116 ºC) (43.4 mg, 85 %); ^{1}H NMR (400 MHz, Chloroform-*d*) δ 8.31 (s, 1H), 7.62 (d, *J* = 8.0 Hz, 1H), 7.40 - 7.36 (m, 2H), 7.23 (s, 1H), 7.12 (d, *J* = 8.0 Hz, 3H), 6.72 (d, *J* = 8.0 Hz, 2H), 3.71 (s, 3H); ^{13}C NMR (100 MHz, Chloroform-*d*) δ 157.7, 136.4, 130.0, 129.5, 129.0, 128.5, 122.9, 120.7, 119.6, 114.5, 111.5, 104.5, 55.3.

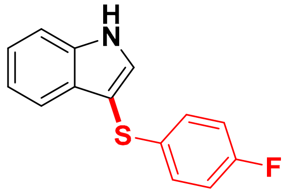

3-((4-Fluorophenyl)thio)-1*H*-indole (5m)[28] It was obtained after purification by column chromatography on silica gel (petroleum ether/ethyl acetate) as white solid (M. p.=143 - 144 ºC) (41.8 mg, 86 %); ^{1}H NMR (400 MHz, Chloroform-*d*) δ 8.40 (s, 1H), 7.59 (d, *J* = 8.0 Hz, 1H), 7.46 - 7.41 (m, 2H), 7.25 (d, *J* = 8.0 Hz, 1H), 7.17 (d, *J* = 8.0 Hz, 1H), 7.08 - 7.07 (m, 2H), 6.86 (t, *J* = 8.0 Hz, 2H); ^{13}C NMR (100 MHz, Chloroform-*d*) δ 160.9 (d, *J* = 243.9 Hz), 136.5, 134.0 (d, *J* = 3.1 Hz), 130.5, 128.8, 127.9 (d, *J* = 7.8 Hz), 123.1, 120.9, 119.5, 115.7 (d, *J* = 22.0 Hz), 111.6, 103.4.

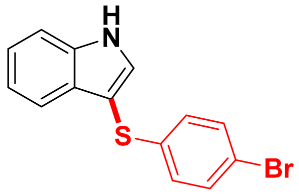

3-((4-Bromophenyl)thio)-1*H*-indole (5n)[28] It was obtained after purification by column chromatography on silica gel (petroleum ether/ethyl acetate) as white solid (M. p.=150 - 151 ºC) (49.7mg, 82 %); ^{1}H NMR (400 MHz, Chloroform-*d*) δ 8.45 (s, 1H), 7.56 (d, J = 8.0 Hz, 1H), 7.48 (s, 1H), 7.44 (d, J = 8.0 Hz, 1H), 7.29 - 7.25 (m, 3H), 7.18 (d, J = 7.2 Hz, 1H), 6.95 (d, J = 8.0 Hz, 1H); ^{13}C NMR (100 MHz, Chloroform-*d*) δ 138.5, 136.5, 131.6, 130.7, 128.8, 127.4, 123.2, 121.1, 119.5, 118.3, 111.7, 102.3.

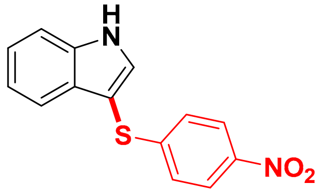

3-((4-Nitrophenyl)thio)-1*H*-indole (5o)[32] It was obtained after purification by column chromatography on silica gel (petroleum ether/ethyl acetate) as yellow solid (M. p.=121 - 122 ºC) (41.6 mg, 77 %); ^{1}H NMR (400 MHz, Chloroform-*d*) δ 8.67 (s, 1H), 7.98 (d, J = 8.0 Hz, 2H), 7.53 - 7.48 (m, 3H), 7.31 (s, 1H), 7.19 (s, 1H), 7.12 (d, J = 8.0 Hz, 2H); ^{13}C NMR (101 MHz, Chloroform-*d*) δ 149.9, 144.8, 136.6, 131.2, 128.4, 125.1, 123.8, 123.5, 121.4, 119.2, 111.9, 100.1.

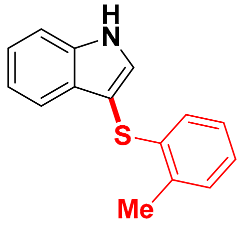

3-(*o*-Tolylthio)-1*H*-indole (5p)[39] It was obtained after purification by column chromatography on silica gel (petroleum ether/ethyl acetate) as yellow oil (41.6 mg, 87 %); ^{1}H NMR (400 MHz, Chloroform-*d*) δ 8.31 (s, 1H), 7.57 (d, J = 8.0 Hz, 1H), 7.40 (s, 2H), 7.25 - 7.21 (m, 1H), 7.16 - 7.11 (m, 2H), 6.95 (t, J = 6.8 Hz, 1H), 6.88 (t, J = 6.8 Hz, 1H), 6.71 (d, J = 8.0 Hz, 1H), 2.49 (s, 3H); ^{13}C NMR (100 MHz, Chloroform-d) δ 138.3, 136.6, 134.4, 130.9, 129.9, 129.3, 126.3, 125.3, 124.5, 123.1, 120.9, 119.7, 111.7, 102.3, 20.0.

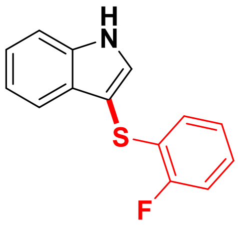

3-((2-Fluorophenyl)thio)-1*H*-indole (5r)[27] It was obtained after purification by column chromatography on silica gel (petroleum ether/ethyl acetate) as white solid (M.p.=145-146 °C) (40.8 mg, 84 %); ^{1}H NMR (400 MHz, Chloroform-*d*) δ 8.43 (s, 1H), 7.62 (d, *J* = 8.0 Hz, 1H), 7.48 (s, 1H), 7.43 (d, *J* = 8.0 Hz, 1H), 7.27 (t, *J* = 7.2 Hz, 1H), 7.17 (t, *J* = 7.2 Hz, 1H), 7.01 (d, *J* = 8.0 Hz, 2H), 6.83 - 6.77 (m, 2H); ^{13}C NMR (100 MHz, Chloroform-d) *δ* 159.0 (d, *J* = 243.6 Hz), 136.5, 131.2, 128.0 (d, *J* = 2.4 Hz), 126.4 (d, *J* = 16.7 Hz), 126.2 (d, *J* = 7.4 Hz), 124.3 (d, *J* = 3.4 Hz), 123.1, 121.0, 119.5, 115.1 (d, *J* = 21.1 Hz), 111.7, 100.7.

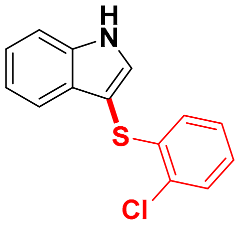

3-((2-Chlorophenyl)thio)-1*H*-indole (5s)[27] It was obtained after purification by column chromatography on silica gel (petroleum ether/ethyl acetate) as white solid (M.p.=135-137 °C) (41.4 mg, 80 %); ^{1}H NMR (400 MHz, Chloroform-*d*) *δ* 8.45 (s, 1H), 7.58 (d, *J* = 8.0 Hz, 1H), 7.44 (d, *J* = 12.0 Hz, 2H), 7.32 - 7.28 (m, 2H), 7.17 (s, 1H), 6.96 (d, *J* = 7.2 Hz, 1H), 6.90 (d, *J* = 7.2 Hz, 1H), 6.64 (d, *J* = 8.0 Hz, 1H); ^{13}C NMR (100 MHz, Chloroform-*d*) *δ* 138.5, 136.5, 131.3, 130.0, 129.2, 128.9, 126.9, 126.3, 125.4, 123.2, 121.1, 119.5, 111.7, 101.1.

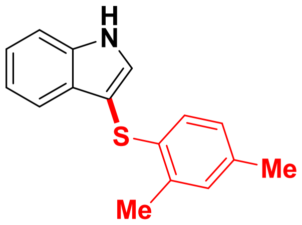

3-((2,4-Dimethylphenyl)thio)-1*H*-indole (5t)[32] It was obtained after purification by column chromatography on silica gel (petroleum ether/ethyl acetate) as a white solid (M.p.=116-118 °C) (42.0 mg, 83 %); ^{1}H NMR (400 MHz, Chloroform-*d*) *δ* 8.29 (s, 1H), 7.57 (d, *J* = 8.0 Hz, 1H), 7.37 (s, 2H), 7.23 (d,

J = 8.0 Hz, 1H), 7.14 (d, *J* = 8.0 Hz, 1H), 6.96 (s, 1H), 6.70 - 6.64 (m, 2H), 2.46 (s, 3H), 2.21 (s, 3H); ^{13}C NMR (100 MHz, Chloroform-*d*) δ 136.5, 134.5, 134.3, 130.8, 130.5, 129.2, 127.0, 125.8, 122.9, 120.8, 119.6, 111.5, 102.8, 20.7, 19.9.

References

19. Song, S.; Zhang, Y.; Yeerlan, A.; Zhu, B.; Liu, J.; Jiao, N. *Angew. Chem. Int. Ed.* **2017**, *56*, 2487-2491.

22. Zhang, H.; Zhan, Z.; Lin, Y.; Shi, Y.; Li, G.; Wang, Q.; Deng, Y.; Hai, L.; Wu, Y. *Org. Chem. Front.* **2018**, *5*, 1416-1422.

23. Shen, J.; Li, Q.-W.; Zhang, X.-Y.; Wang, X.; Li, G.-Z.; Li, W.-Z.; Yang, S.-D.; Yang, B. *Org. Lett.* **2021**, *23*, 1541-1547.

27. Li, J.; Li, C.; Yang, S.; An, Y.; Wu, W.; Jiang, H. *J. Org. Chem.* **2016**, *81*, 7771-7783.

28. Li, W.; Wang, H.; Liu, S.; Feng, H.; Benassi, E.; Qian, B. *Adv. Synth. Catal.* **2020**, *362*, 2666-2671.

31. Yuan, W.; Huang, J.; Xu, X.; Wang, L.; Tang, X. -Y. *Org. Lett.* **2021**, *23*, 7139-7143.

32. Huang, Q.; Peng, X.; Li, H.; He, H.; Liu L. *Molecules* **2022**, *27*, 772-787.

37. Xu, J.; Zhang, L.; Li, X.; Gao, Y.; Tang, G; Zhao, Y. *Org. Lett.* **2016**, *18*, 1266-1269.

38. Xia, M.; Cheng, J. *Tetrahedron Lett.* **2016**, *57*, 4702-4704.

39. Wu, Z.; Li, Y.-C.; Ding, W.-Z.; Zhu, T.; Liu, S.-Z.; Ren, X.; Zou, L.-H. *Asian J. Org. Chem.* **2016**, *5*, 625-628.

NMR Spectra for All the Compounds

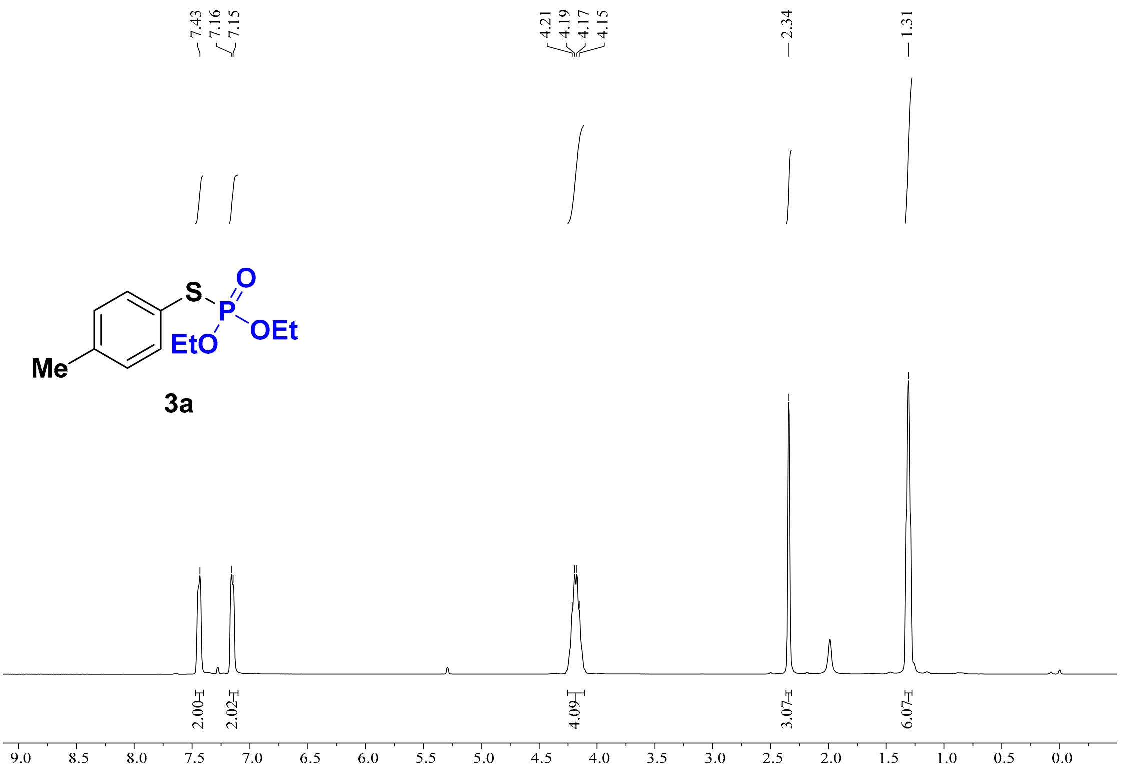

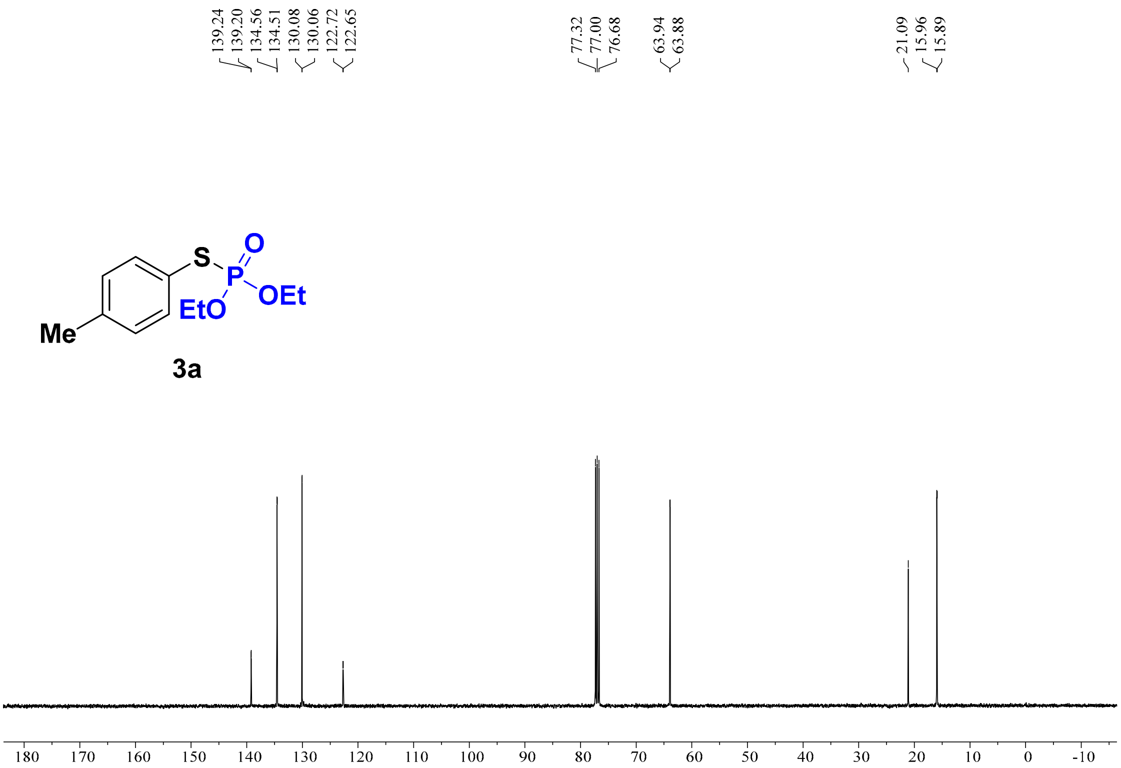

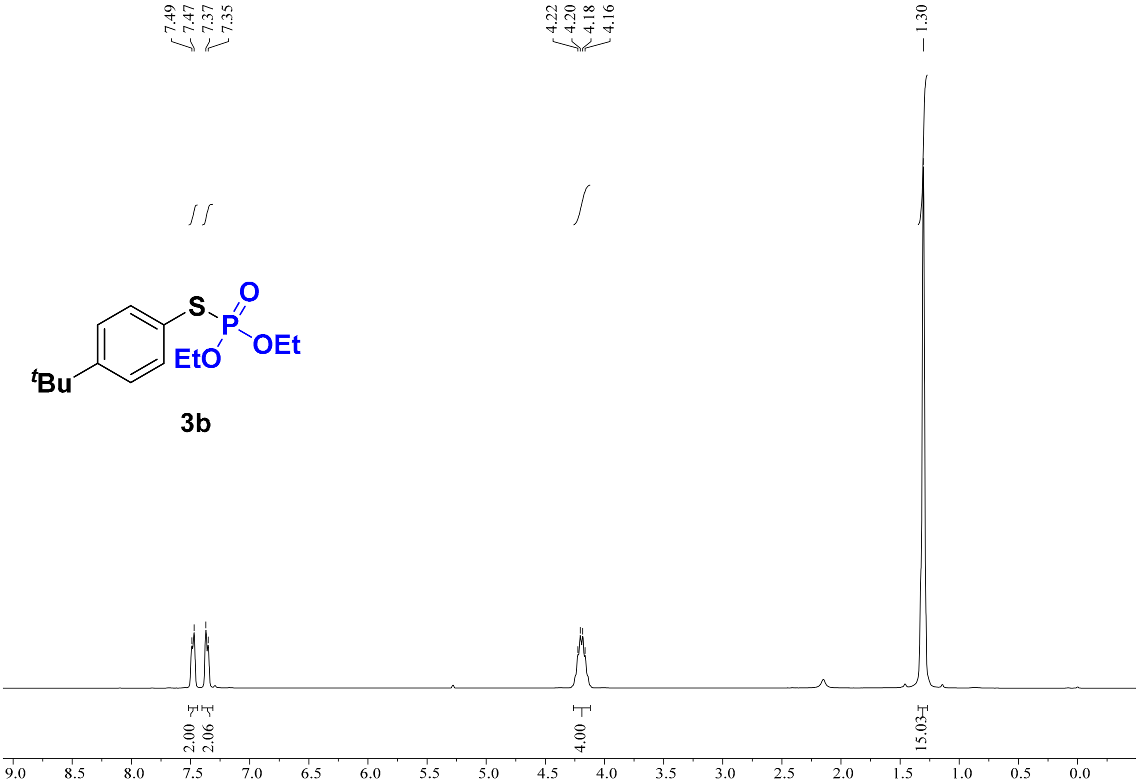
7.49
7.47
7.37
7.35
4.22
4.20
4.18
4.16
1.30
S
P
O
OEt
EtO
tBu
3b
2.00
2.06
4.00
15.03
9.0 8.5 8.0 7.5 7.0 6.5 6.0 5.5 5.0 4.5 4.0 3.5 3.0 2.5 2.0 1.5 1.0 0.5 0.0

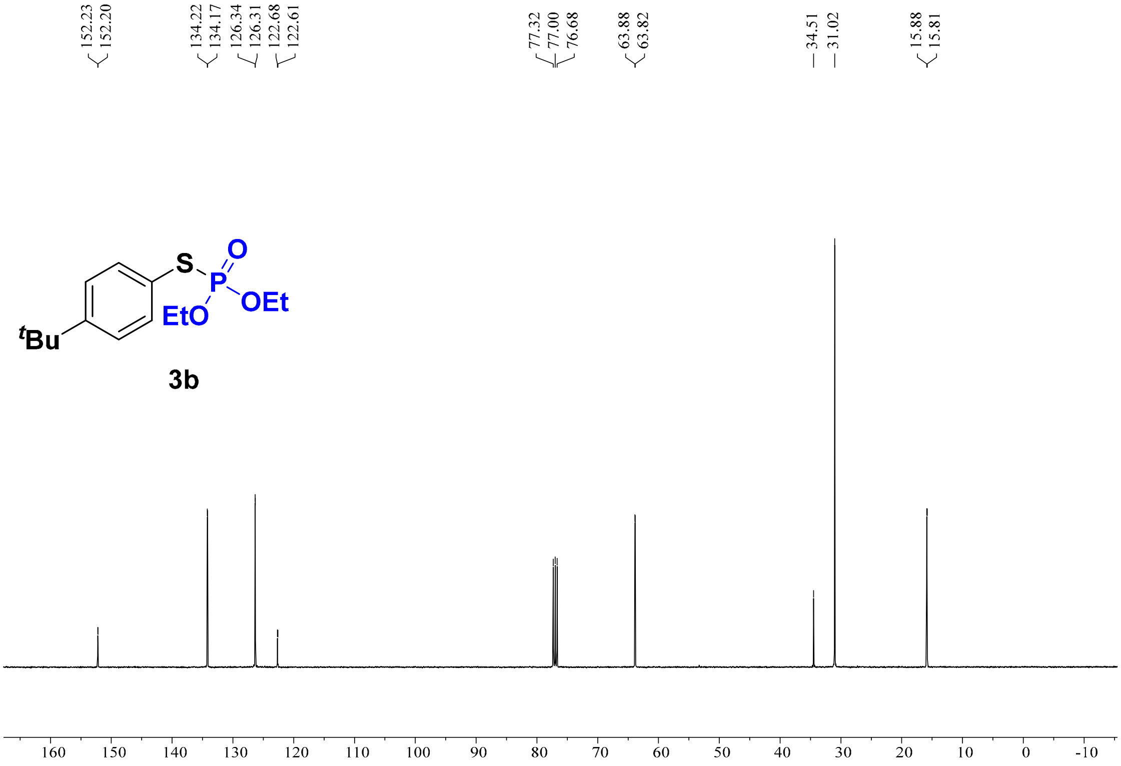
152.23
152.20
134.22
134.17
126.34
126.31
122.68
122.61
77.32
77.00
76.68
63.88
63.82
34.51
31.02
15.88
15.81
S
P
O
OEt
EtO
tBu
3b
160 150 140 130 120 110 100 90 80 70 60 50 40 30 20 10 0 -10

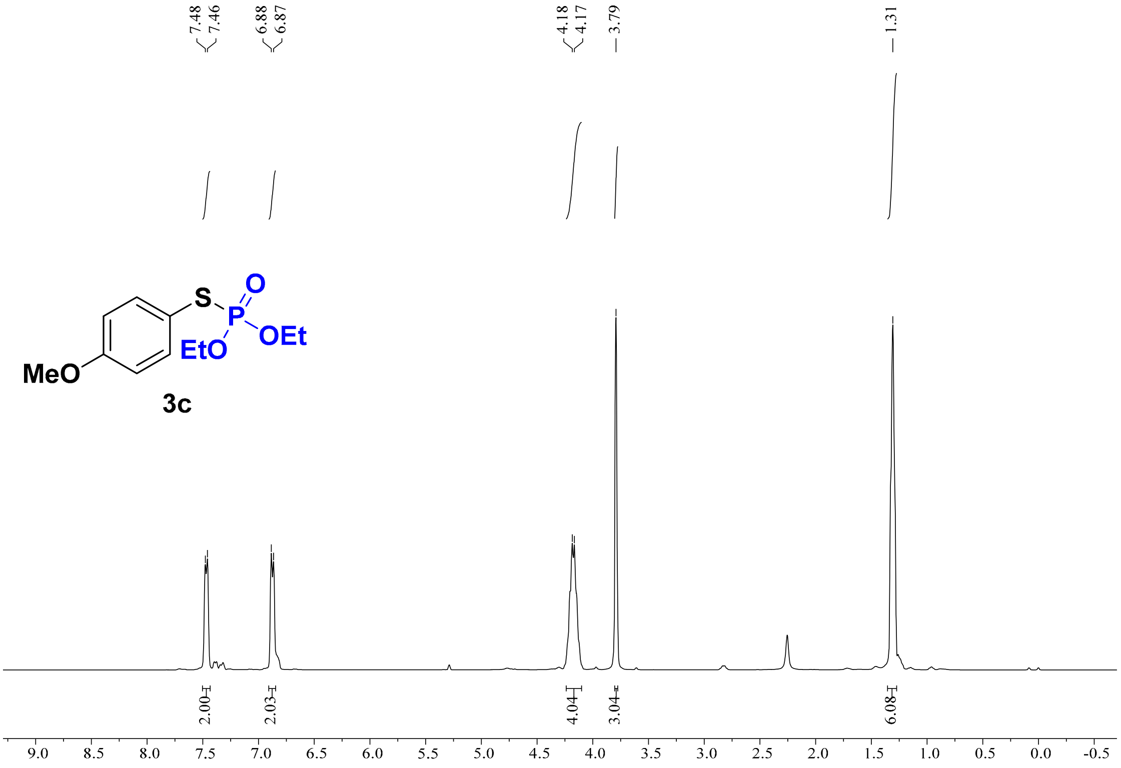
7.48
7.46
6.88
6.87
4.18
4.17
3.79
1.31
S
P
O
OEt
EtO
MeO
3c
2.00
2.03
4.04
3.04
6.08
9.0 8.5 8.0 7.5 7.0 6.5 6.0 5.5 5.0 4.5 4.0 3.5 3.0 2.5 2.0 1.5 1.0 0.5 0.0 -0.5

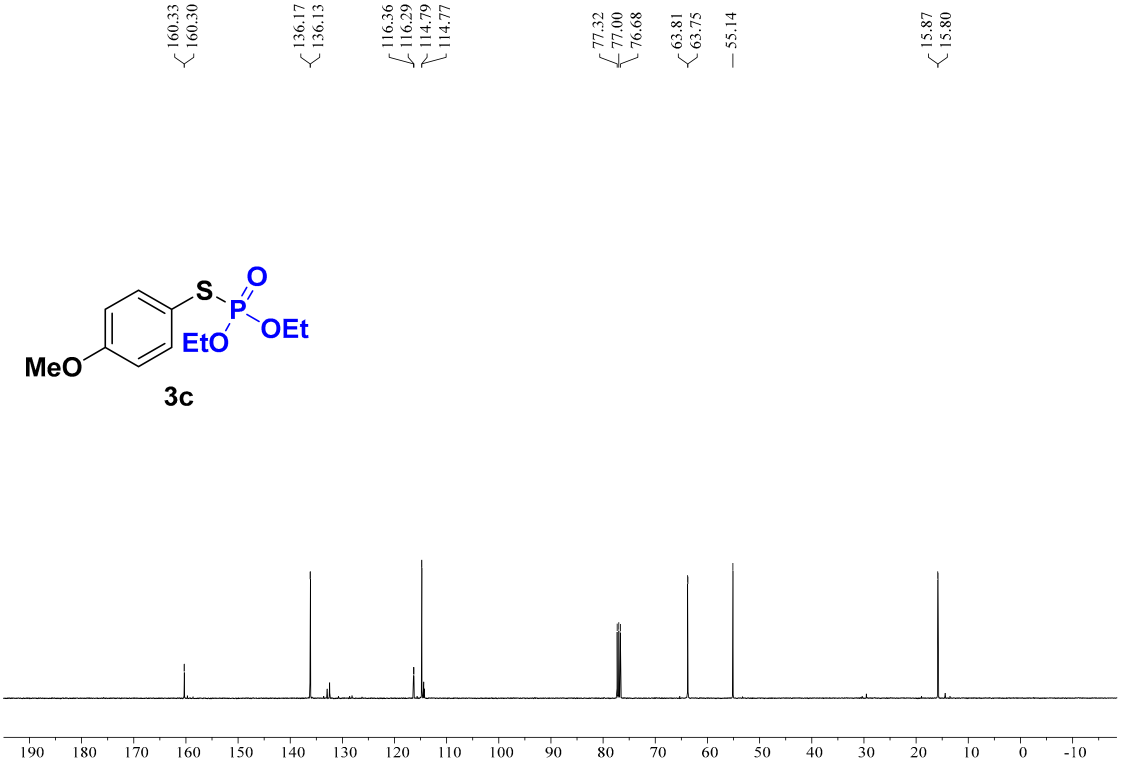
160.33
160.30
136.17
136.13
116.36
116.29
114.79
114.77
77.32
77.00
76.68
63.81
63.75
55.14
15.87
15.80
S
P
O
OEt
EtO
MeO
3c
190 180 170 160 150 140 130 120 110 100 90 80 70 60 50 40 30 20 10 0 -10

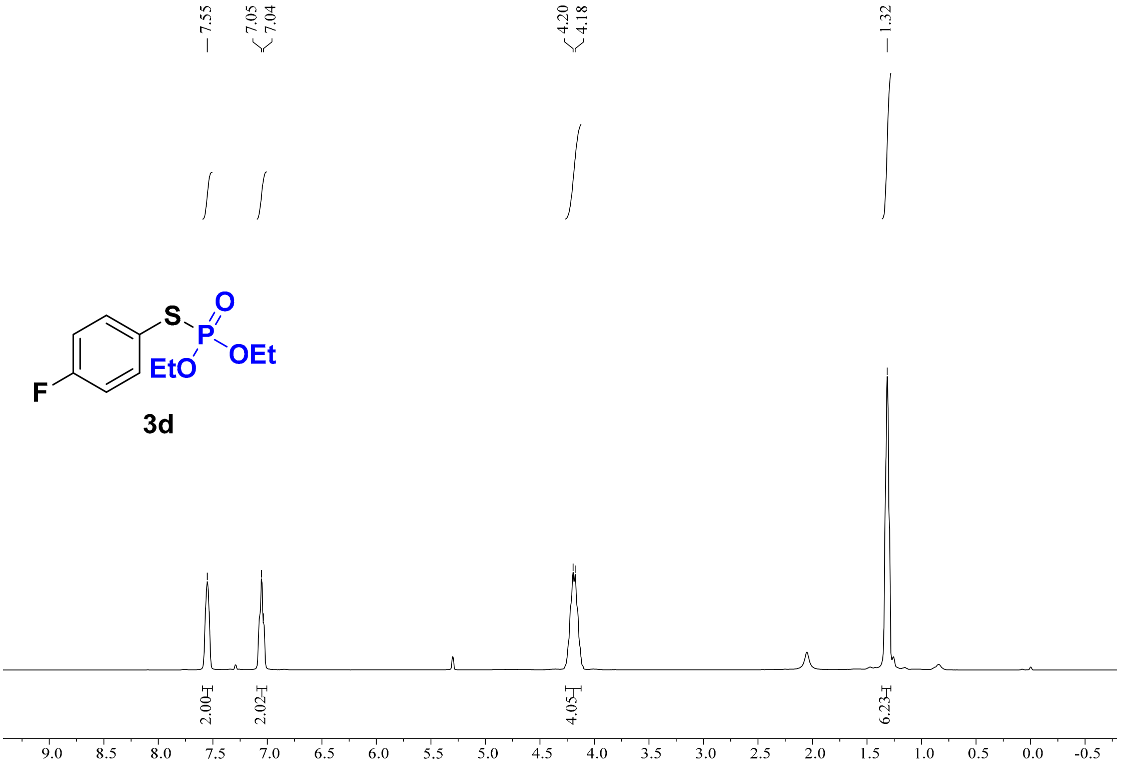
7.55
7.05
7.04
4.20
4.18
1.32
S
O
P
OEt
EtO
F
3d
2.00
2.02
4.05
6.23
9.0 8.5 8.0 7.5 7.0 6.5 6.0 5.5 5.0 4.5 4.0 3.5 3.0 2.5 2.0 1.5 1.0 0.5 0.0 -0.5

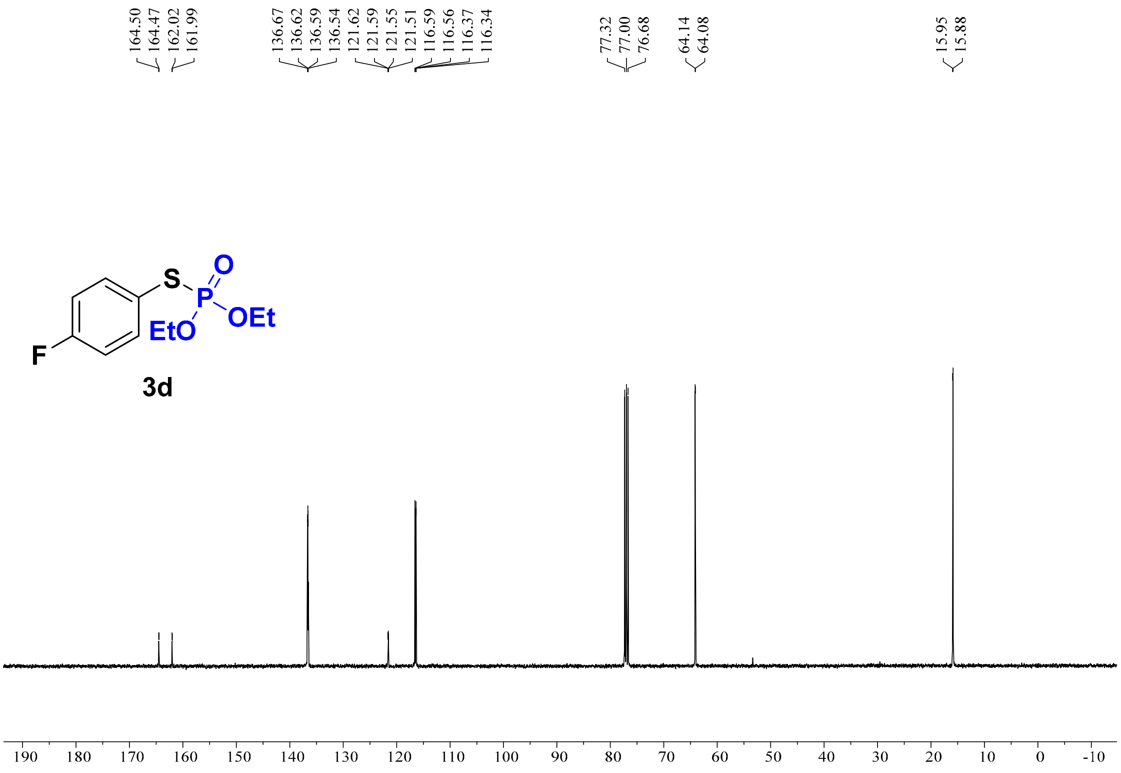
164.50
164.47
162.02
161.99
136.67
136.62
136.59
136.54
121.62
121.59
121.55
121.51
116.59
116.56
116.37
116.34
77.32
77.00
76.68
64.14
64.08
15.95
15.88
S
O
P
OEt
EtO
F
3d
190 180 170 160 150 140 130 120 110 100 90 80 70 60 50 40 30 20 10 0 -10

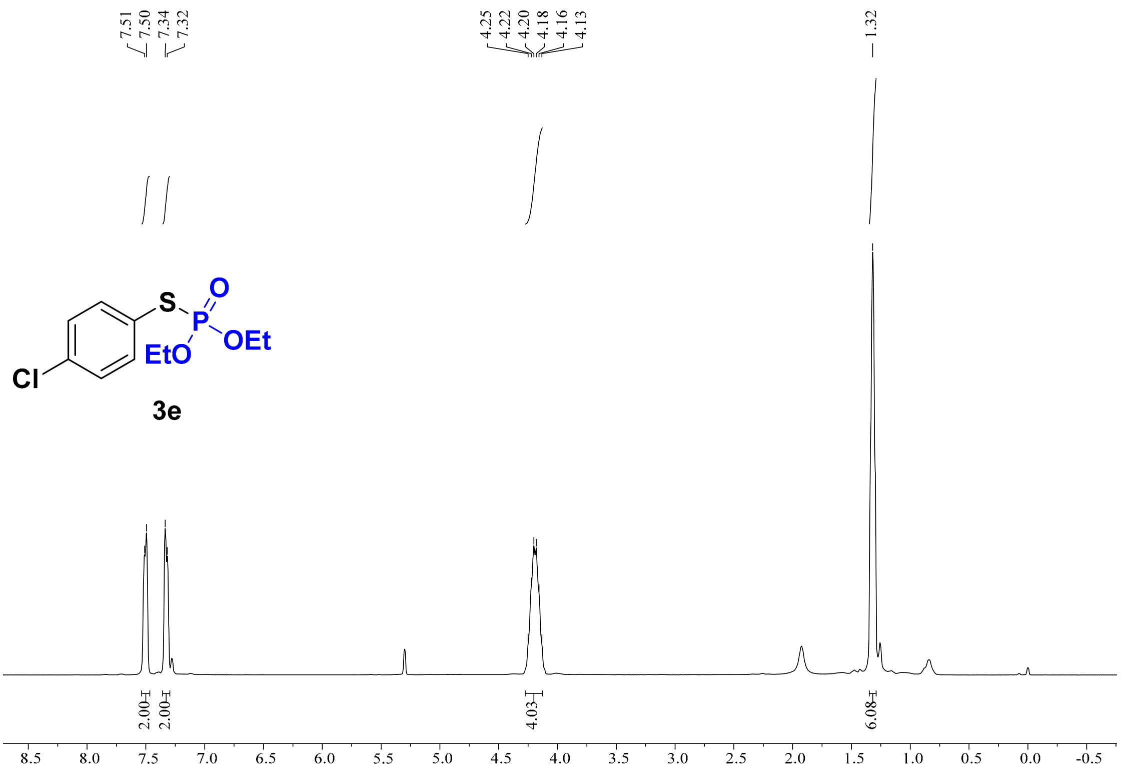
7.51
7.50
7.34
7.32
4.25
4.22
4.20
4.18
4.16
4.13
1.32
S
P
O
OEt
EtO
Cl
3e
2.00
2.00
4.03
6.08
8.5 8.0 7.5 7.0 6.5 6.0 5.5 5.0 4.5 4.0 3.5 3.0 2.5 2.0 1.5 1.0 0.5 0.0 -0.5

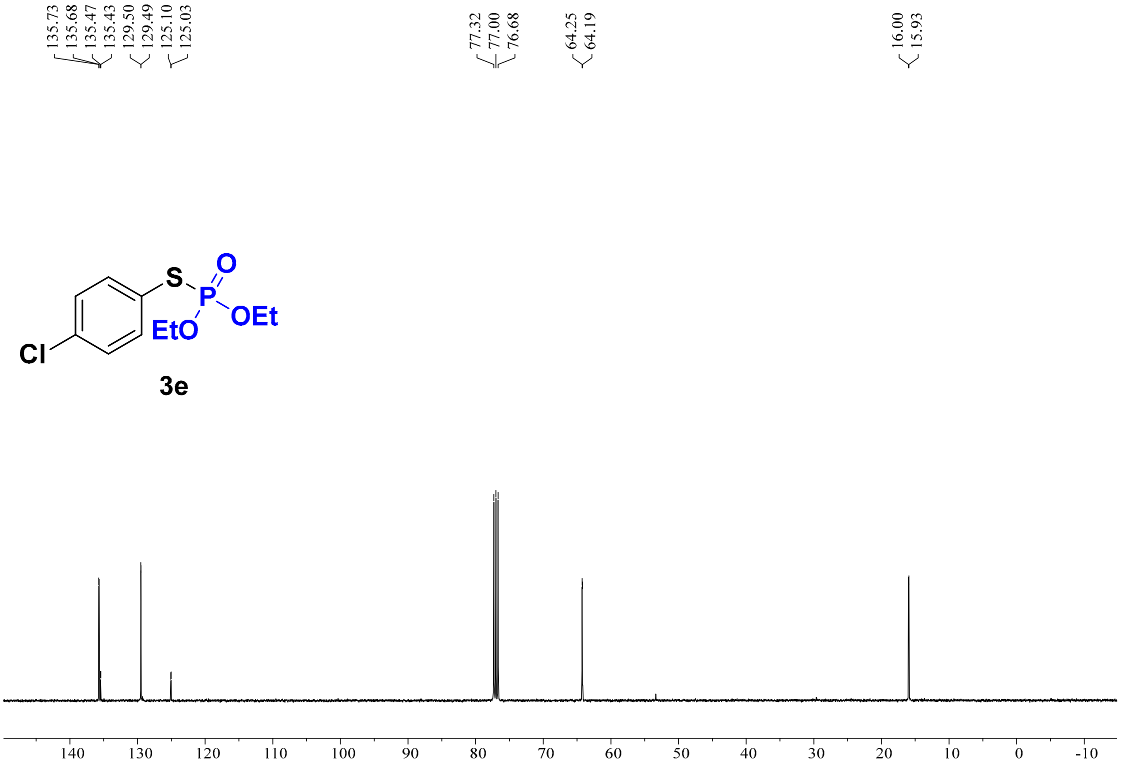
135.73
135.68
135.47
135.43
129.50
129.49
125.10
125.03
77.32
77.00
76.68
64.25
64.19
16.00
15.93
S
P
O
OEt
EtO
Cl
3e
140 130 120 110 100 90 80 70 60 50 40 30 20 10 0 -10

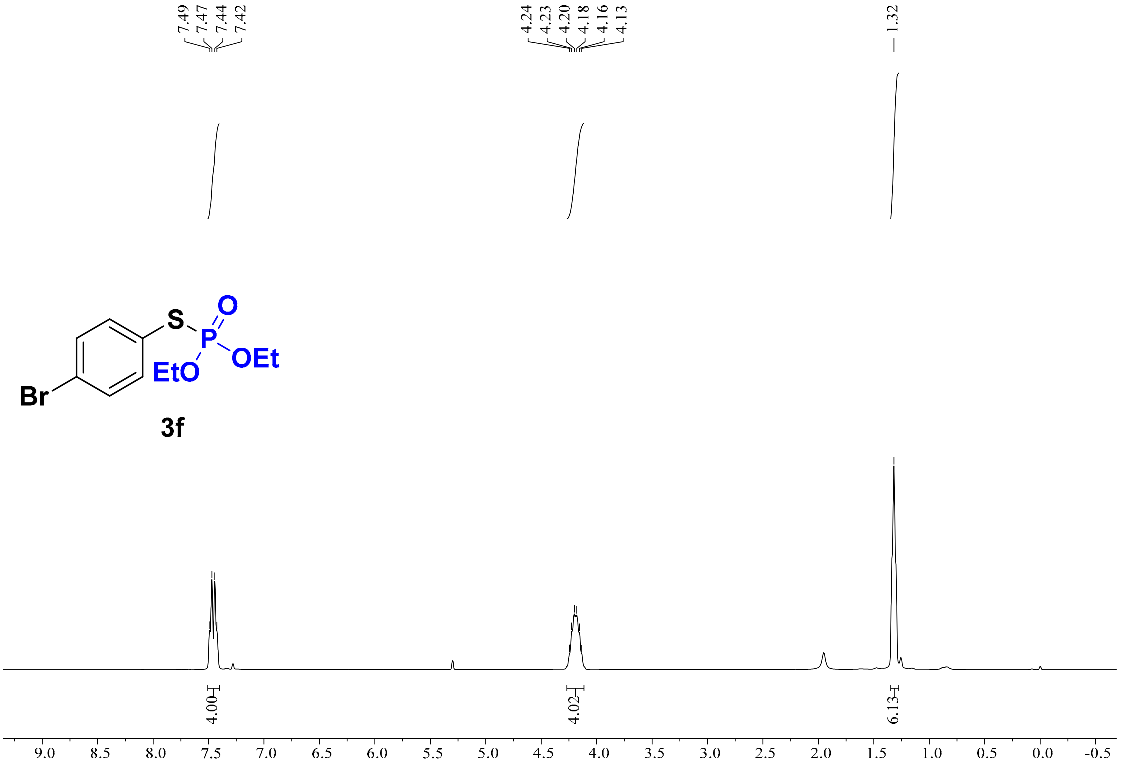
7.49
7.47
7.44
7.42
4.24
4.23
4.20
4.18
4.16
4.13
1.32
S
P
O
OEt
EtO
Br
3f
4.00
4.02
6.13
9.0 8.5 8.0 7.5 7.0 6.5 6.0 5.5 5.0 4.5 4.0 3.5 3.0 2.5 2.0 1.5 1.0 0.5 0.0 -0.5

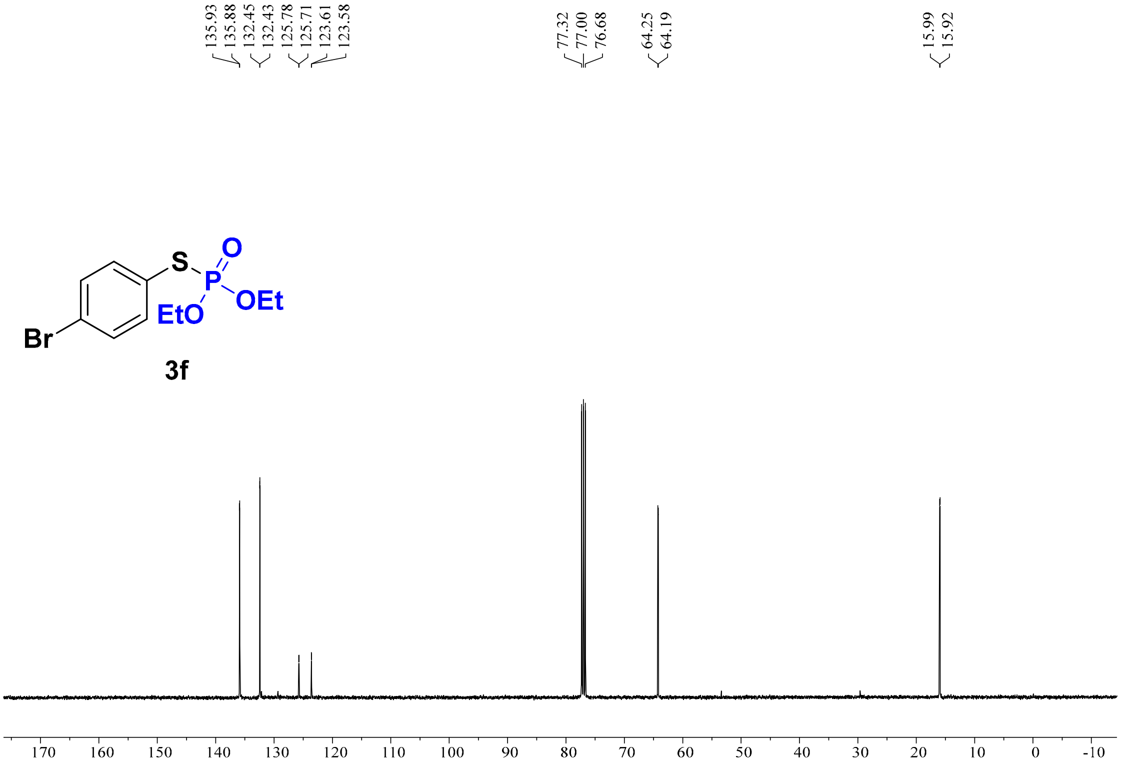
135.93
135.88
132.45
132.43
125.78
125.71
123.61
123.58
77.32
77.00
76.68
64.25
64.19
15.99
15.92
S
P
O
OEt
EtO
Br
3f
170 160 150 140 130 120 110 100 90 80 70 60 50 40 30 20 10 0 -10

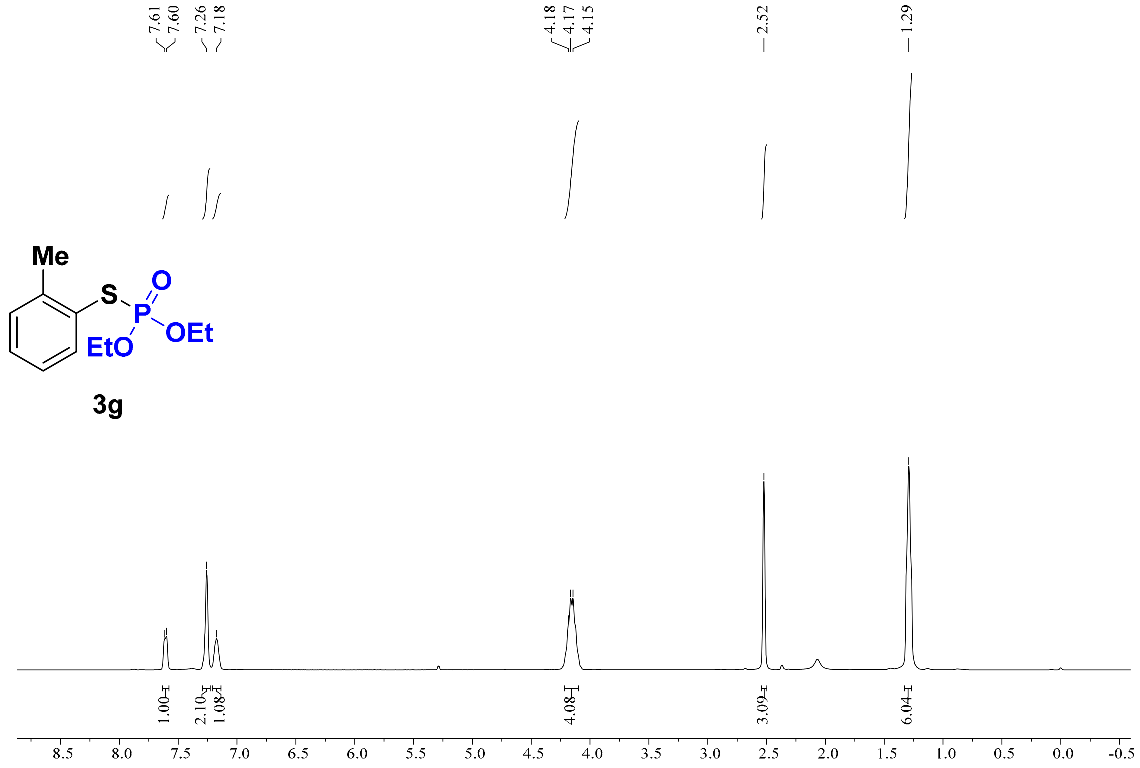
7.61
7.60
7.26
7.18
4.18
4.17
4.15
2.52
1.29
Me
S
P
O
OEt
EtO
3g
1.00
2.10
1.08
4.08
3.09
6.04
8.5 8.0 7.5 7.0 6.5 6.0 5.5 5.0 4.5 4.0 3.5 3.0 2.5 2.0 1.5 1.0 0.5 0.0 -0.5

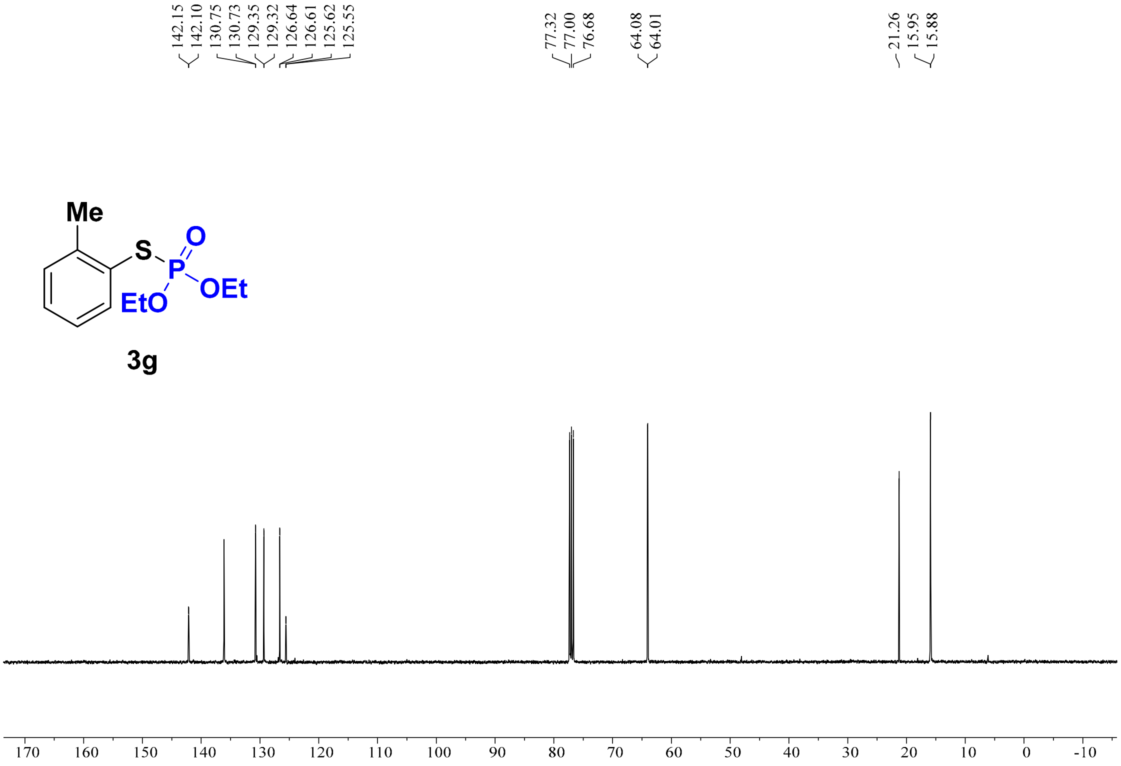
142.15
142.10
130.75
130.73
129.35
129.32
126.64
126.61
125.62
125.55
77.32
77.00
76.68
64.08
64.01
21.26
15.95
15.88
Me
S
P
O
OEt
EtO
3g
170 160 150 140 130 120 110 100 90 80 70 60 50 40 30 20 10 0 -10

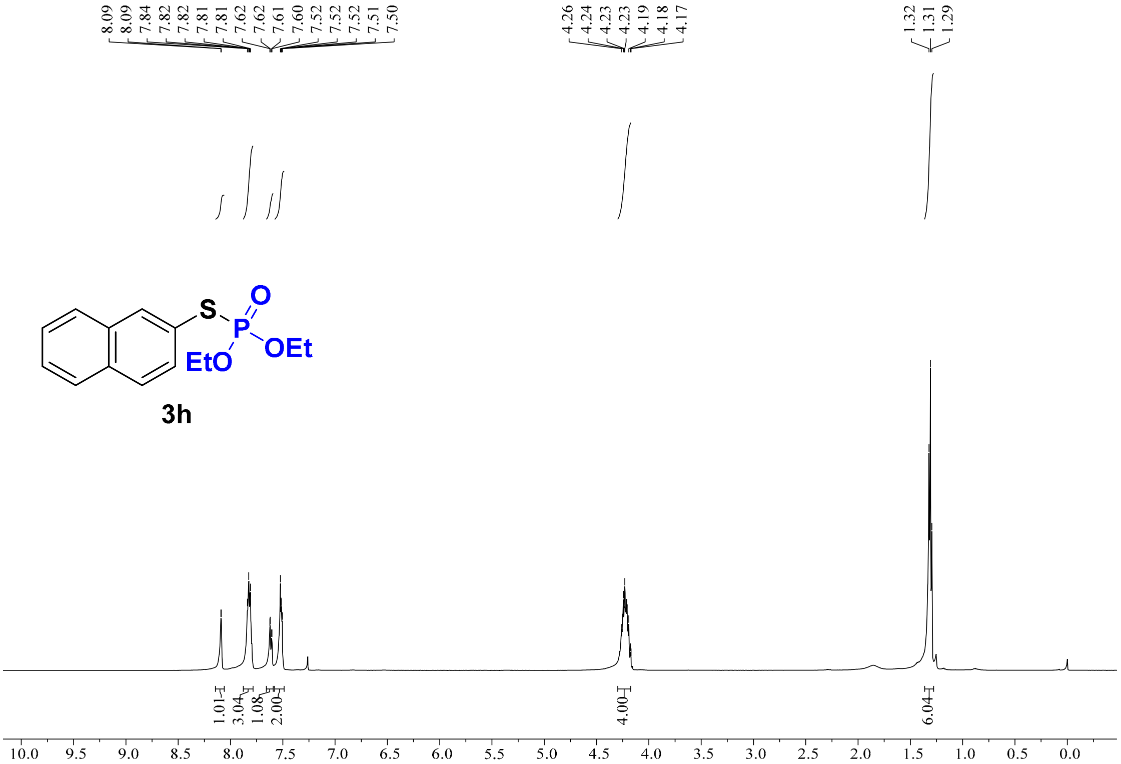
8.09 8.09 7.84 7.82 7.82 7.81 7.81 7.62 7.62 7.61 7.60 7.52 7.52 7.52 7.51 7.50
4.26 4.24 4.23 4.23 4.19 4.18 4.17
1.32 1.31 1.29
S
O
P
OEt
EtO
3h
1.01
3.04
1.08
2.00
4.00
6.04
10.0 9.5 9.0 8.5 8.0 7.5 7.0 6.5 6.0 5.5 5.0 4.5 4.0 3.5 3.0 2.5 2.0 1.5 1.0 0.5 0.0

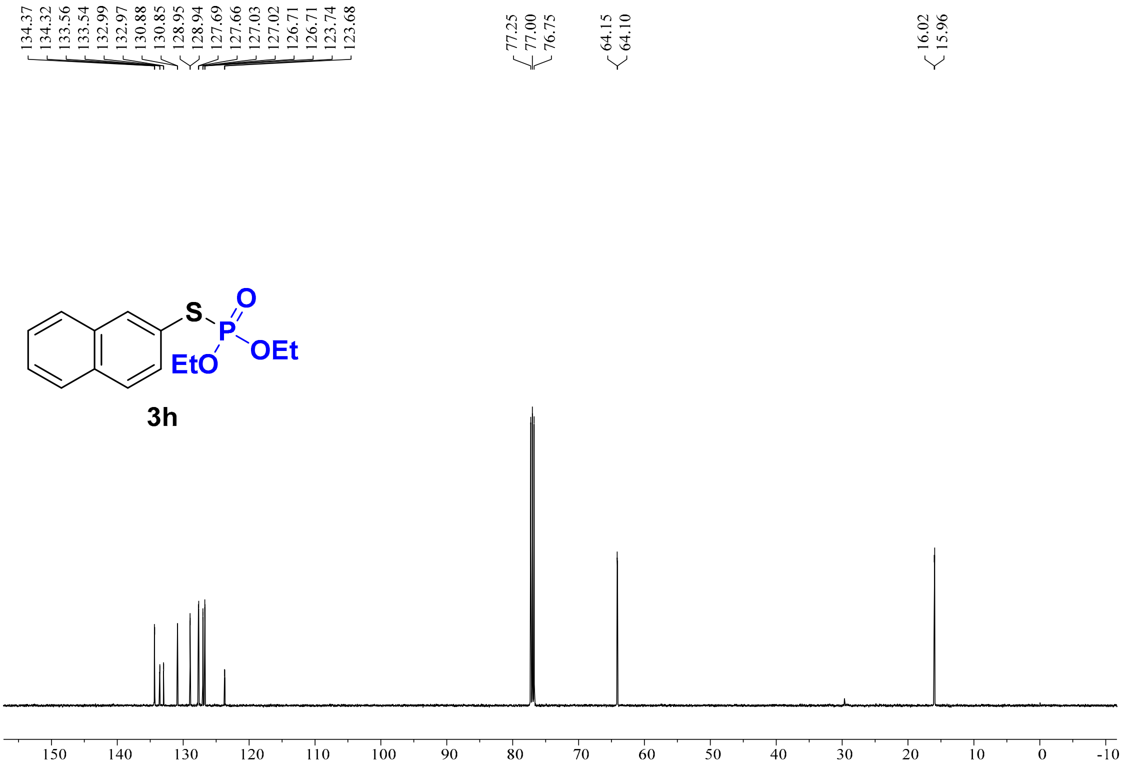
134.37 134.32 133.56 133.54 132.99 132.97 130.88 130.85 128.95 128.94 127.69 127.66 127.03 127.02 126.71 126.71 123.74 123.68
77.25 77.00 76.75
64.15 64.10
16.02 15.96
S
O
P
OEt
EtO
3h
150 140 130 120 110 100 90 80 70 60 50 40 30 20 10 0 -10

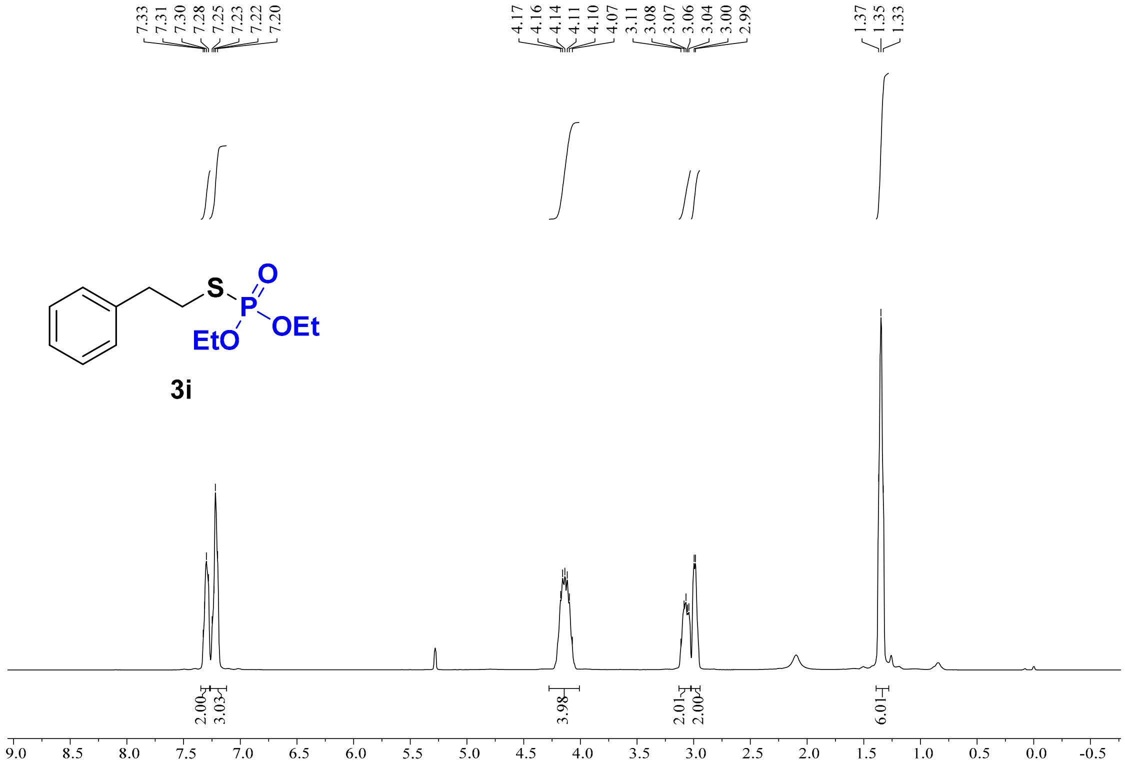
7.33 7.31 7.30 7.28 7.25 7.23 7.22 7.20
4.17 4.16 4.14 4.11 4.10 4.07 3.11 3.08 3.07 3.06 3.04 3.00 2.99
1.37 1.35 1.33
S
O
P
OEt
EtO
3i
2.00 3.03
3.98
2.01 2.00
6.01
9.0 8.5 8.0 7.5 7.0 6.5 6.0 5.5 5.0 4.5 4.0 3.5 3.0 2.5 2.0 1.5 1.0 0.5 0.0 -0.5

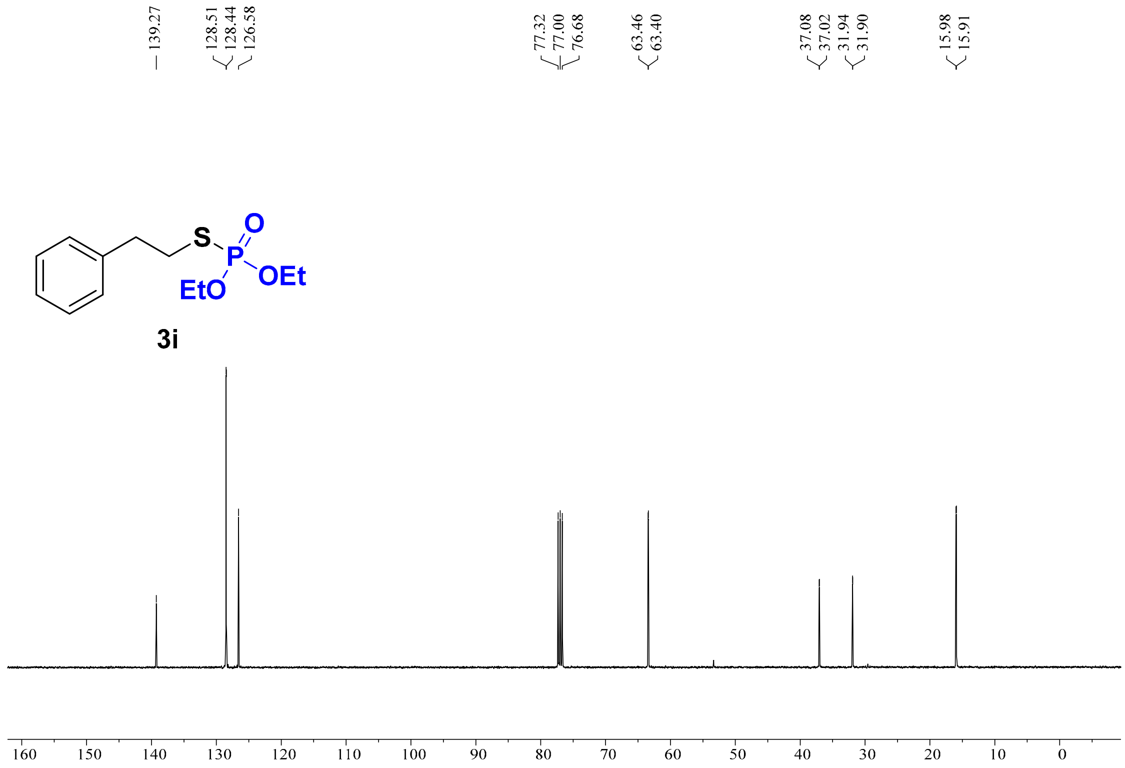
139.27
128.51 128.44 126.58
77.32 77.00 76.68
63.46 63.40
37.08 37.02 31.94 31.90
15.98 15.91
S
O
P
OEt
EtO
3i
160 150 140 130 120 110 100 90 80 70 60 50 40 30 20 10 0

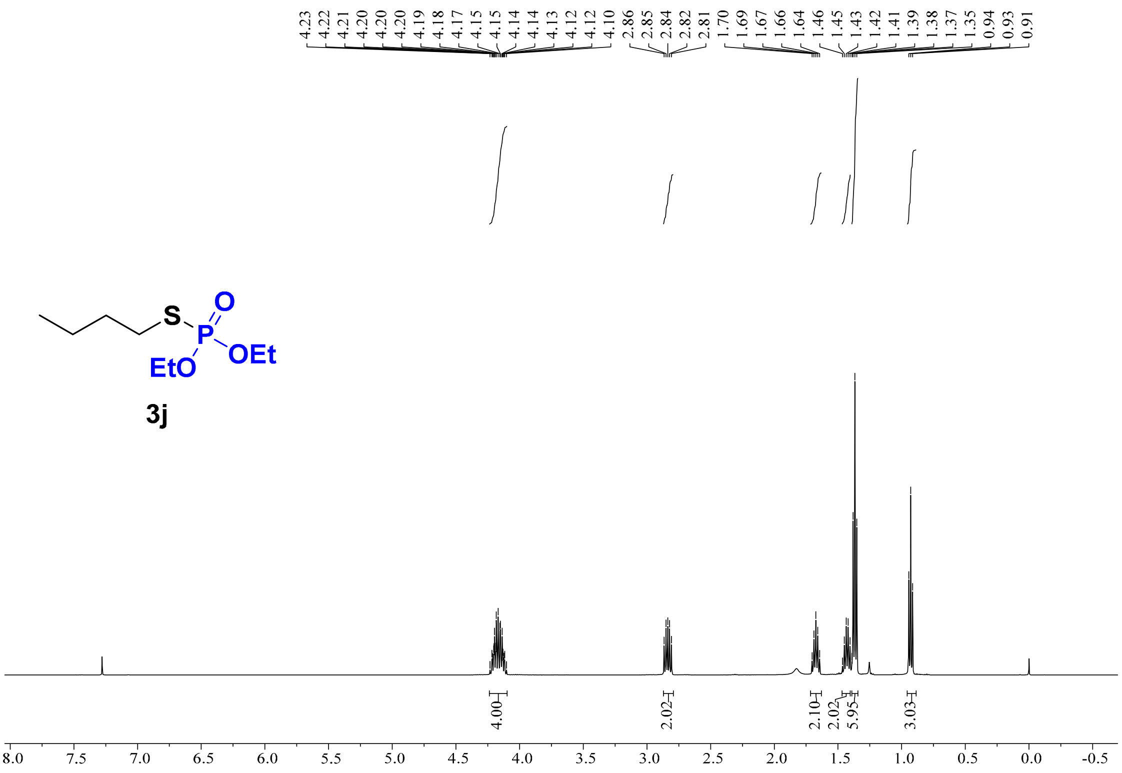
4.23 4.22 4.21 4.20 4.20 4.20 4.19 4.18 4.17 4.15 4.15 4.14 4.14 4.13 4.12 4.12 4.10 2.86 2.85 2.84 2.82 2.81 1.70 1.69 1.67 1.66 1.64 1.46 1.45 1.43 1.42 1.41 1.39 1.38 1.37 1.35 0.94 0.93 0.91
S
O
P
OEt
EtO
3j
4.00
2.02
2.10
2.02
5.95
3.03
8.0 7.5 7.0 6.5 6.0 5.5 5.0 4.5 4.0 3.5 3.0 2.5 2.0 1.5 1.0 0.5 0.0 -0.5

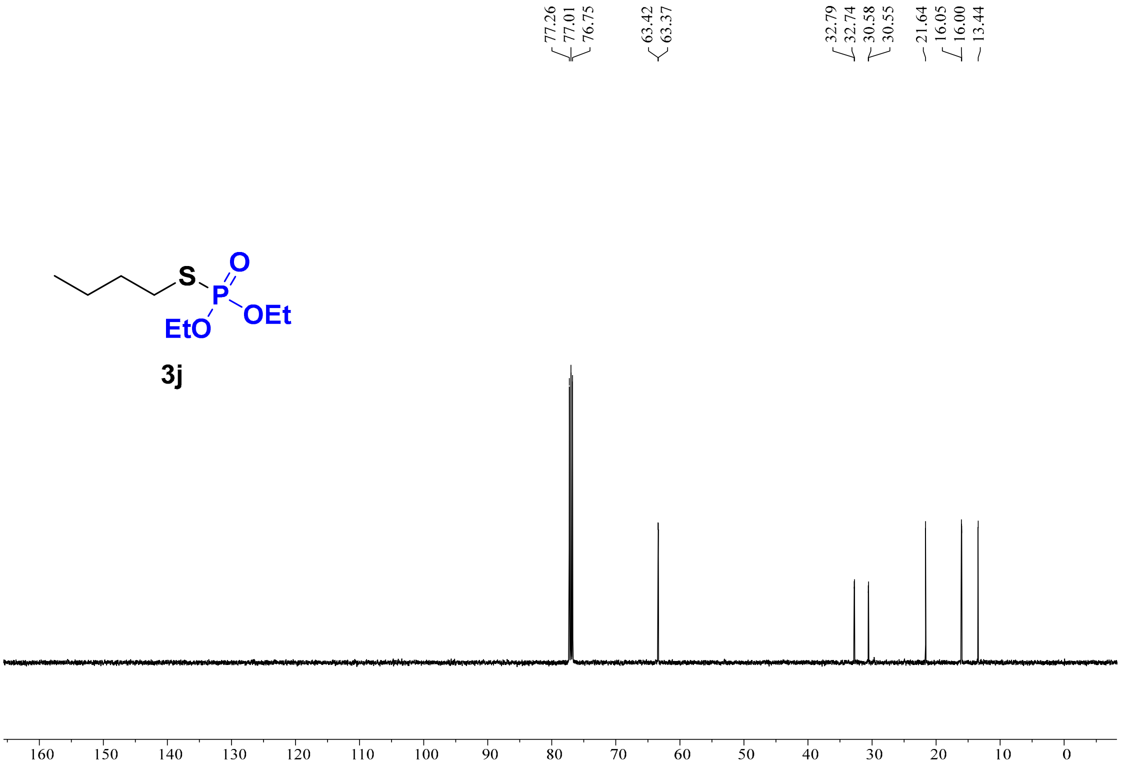
77.26 77.01 76.75
63.42 63.37
32.79 32.74 30.58 30.55
21.64 16.05 16.00 13.44
S
O
P
OEt
EtO
3j
160 150 140 130 120 110 100 90 80 70 60 50 40 30 20 10 0

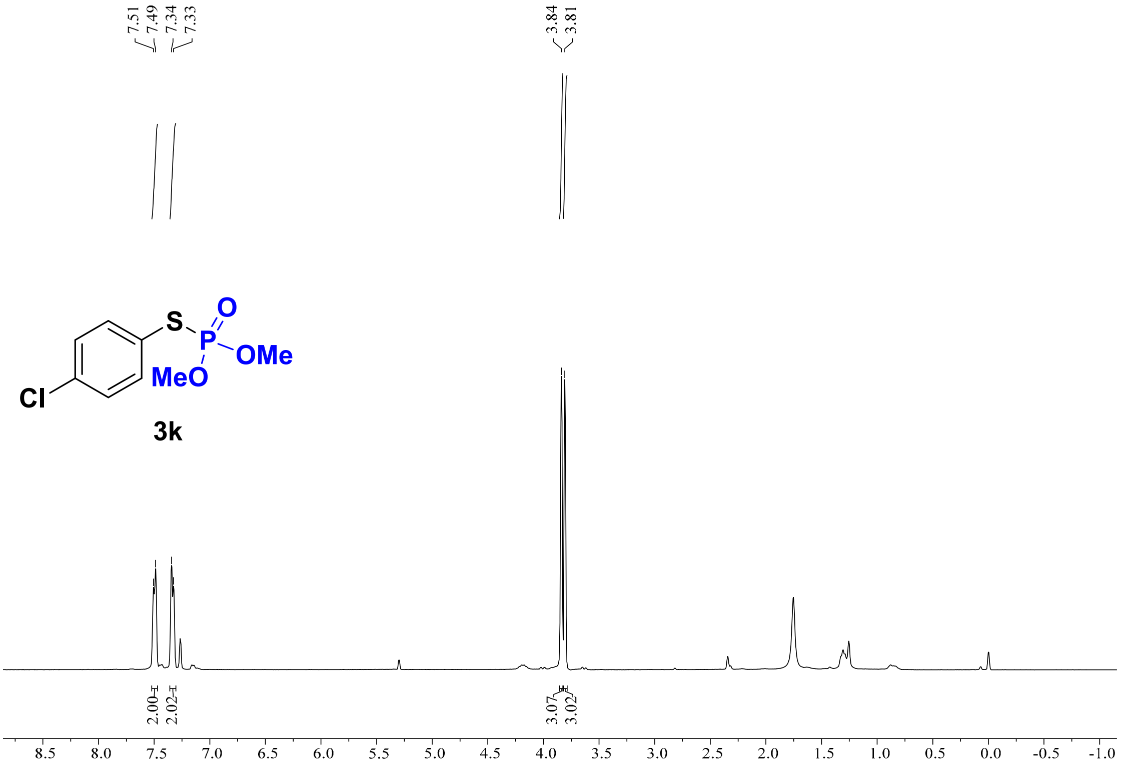
7.51
7.49
7.34
7.33
3.84
3.81
S
O
P
OMe
MeO
Cl
3k
2.00
2.02
3.07
3.02
8.5 8.0 7.5 7.0 6.5 6.0 5.5 5.0 4.5 4.0 3.5 3.0 2.5 2.0 1.5 1.0 0.5 0.0 -0.5 -1.0

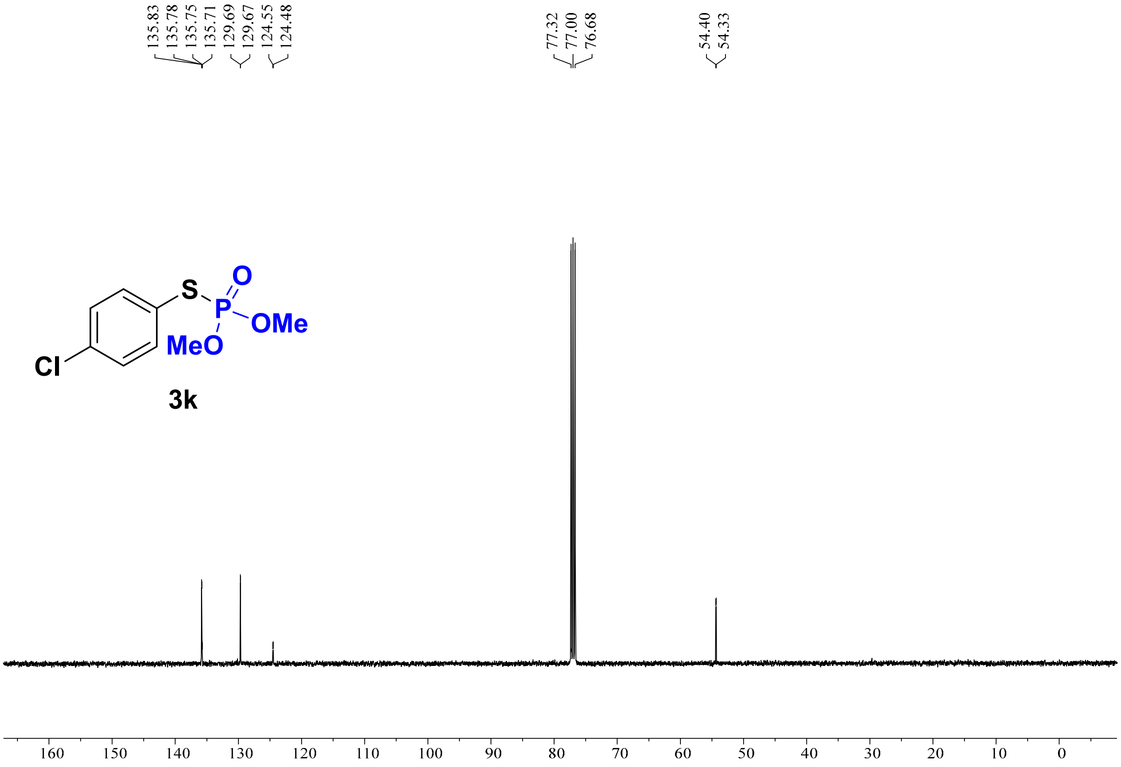
135.83
135.78
135.75
135.71
129.69
129.67
124.55
124.48
77.32
77.00
76.68
54.40
54.33
S
O
P
OMe
MeO
Cl
3k
160 150 140 130 120 110 100 90 80 70 60 50 40 30 20 10 0

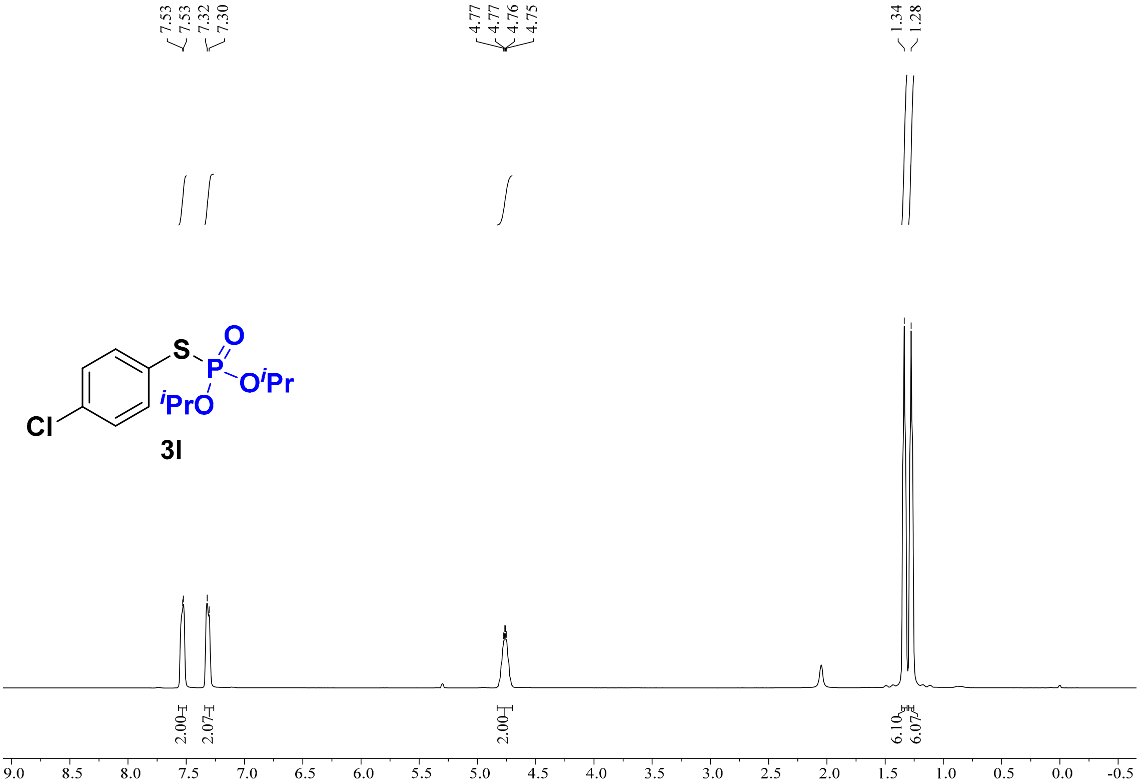

7.53
7.53
7.32
7.30
4.77
4.77
4.76
4.75
1.34
1.28
S
O
P
O^{i}Pr
^{i}PrO
Cl
3l
2.00
2.07
2.00
6.10
6.07
9.0 8.5 8.0 7.5 7.0 6.5 6.0 5.5 5.0 4.5 4.0 3.5 3.0 2.5 2.0 1.5 1.0 0.5 0.0 -0.5

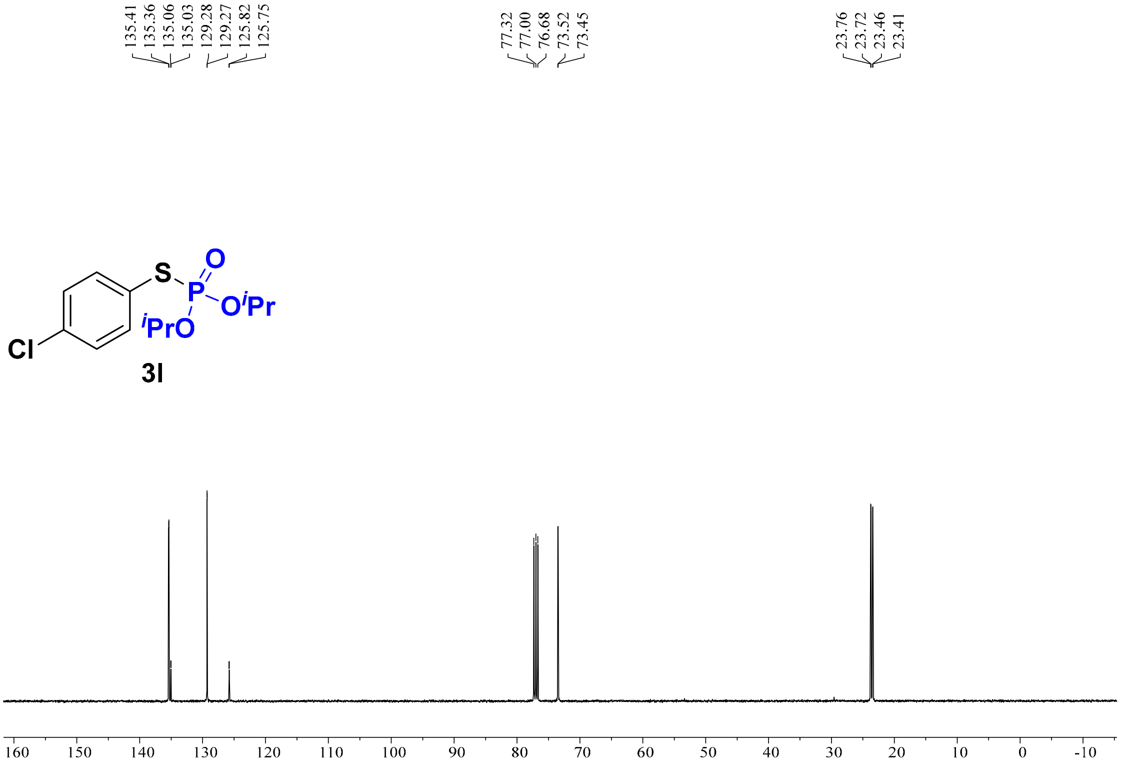

135.41
135.36
135.06
135.03
129.28
129.27
125.82
125.75
77.32
77.00
76.68
73.52
73.45
23.76
23.72
23.46
23.41
S
O
P
O^{i}Pr
^{i}PrO
Cl
3l
160 150 140 130 120 110 100 90 80 70 60 50 40 30 20 10 0 -10

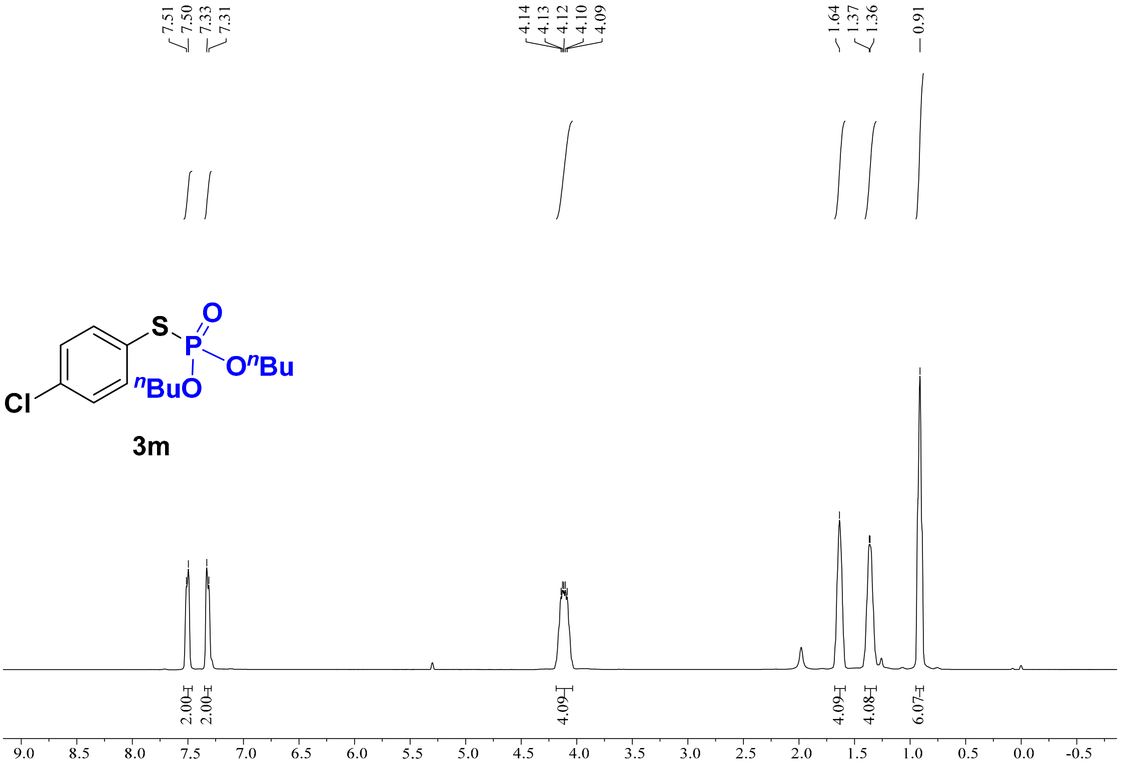
7.51
7.50
7.33
7.31
4.14
4.13
4.12
4.10
4.09
1.64
1.37
1.36
0.91
S
P
O
O^{n}Bu
^{n}BuO
Cl
3m
2.00
2.00
4.09
4.09
4.08
6.07
9.0 8.5 8.0 7.5 7.0 6.5 6.0 5.5 5.0 4.5 4.0 3.5 3.0 2.5 2.0 1.5 1.0 0.5 0.0 -0.5

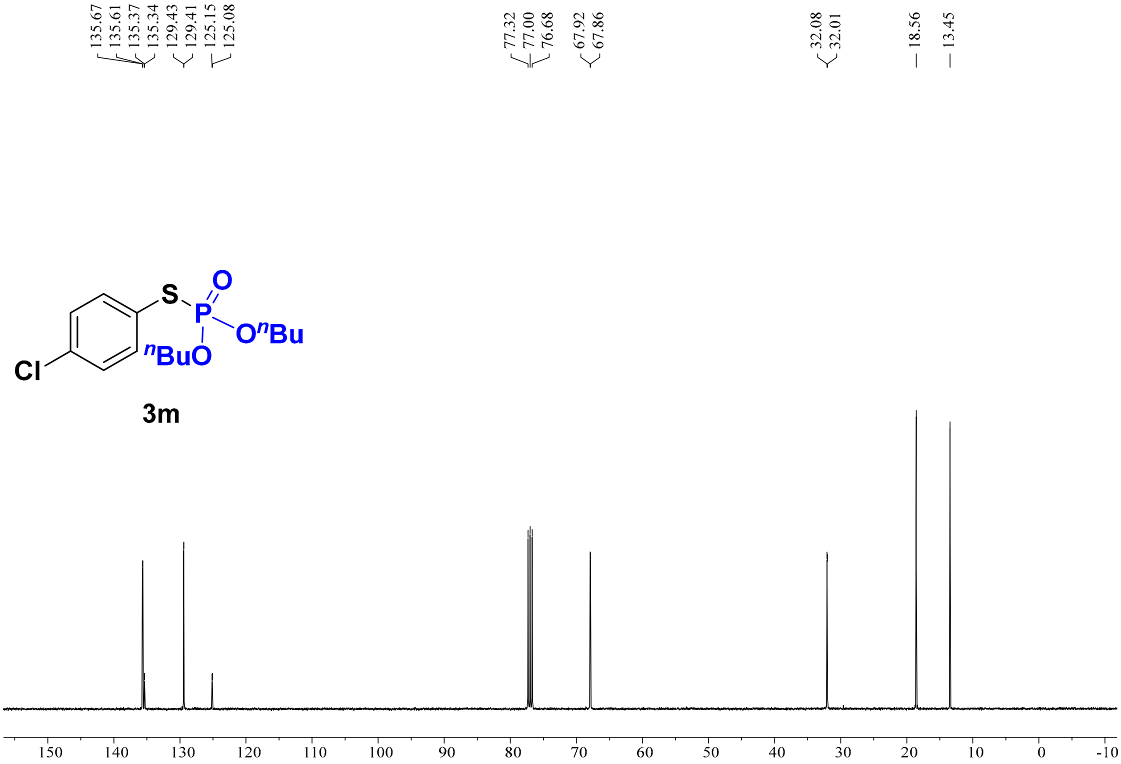
135.67
135.61
135.37
135.34
129.43
129.41
125.15
125.08
77.32
77.00
76.68
67.92
67.86
32.08
32.01
18.56
13.45
S
P
O
O^{n}Bu
^{n}BuO
Cl
3m
150 140 130 120 110 100 90 80 70 60 50 40 30 20 10 0 -10

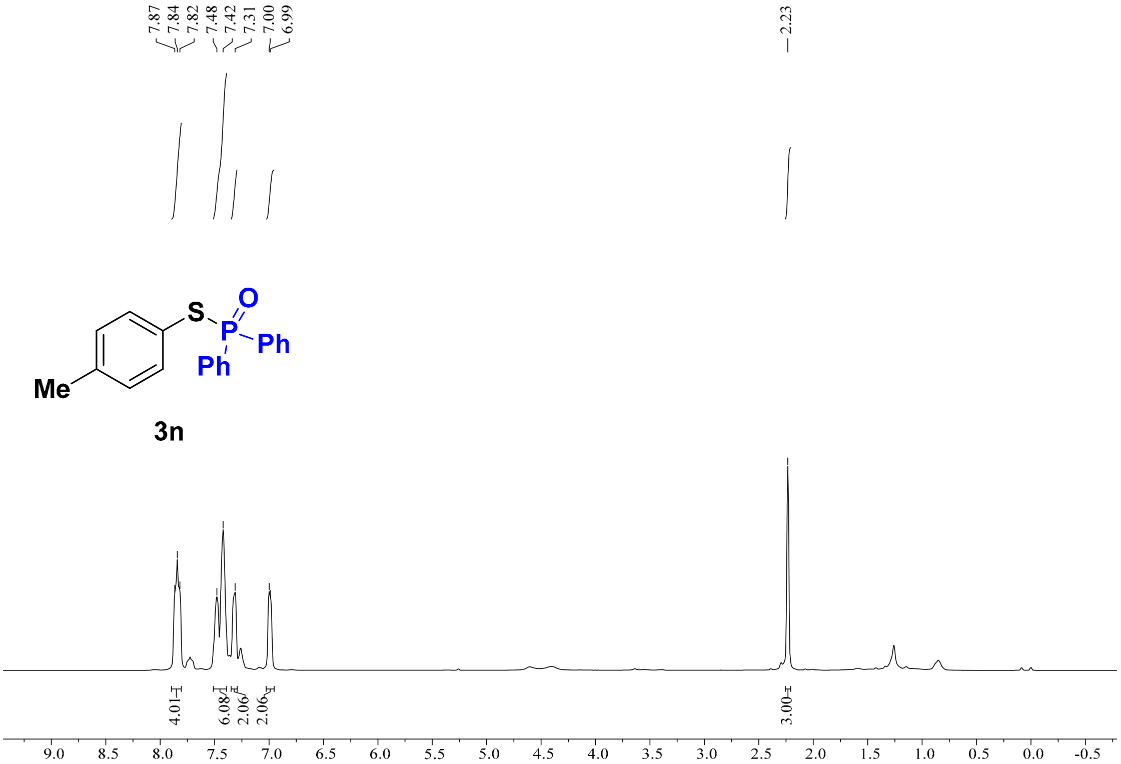
7.87
7.84
7.82
7.48
7.42
7.31
7.00
6.99
2.23
Me
S
P
O
Ph
Ph
3n
4.01
6.08
2.06
2.06
3.00
9.0
8.5
8.0
7.5
7.0
6.5
6.0
5.5
5.0
4.5
4.0
3.5
3.0
2.5
2.0
1.5
1.0
0.5
0.0
-0.5

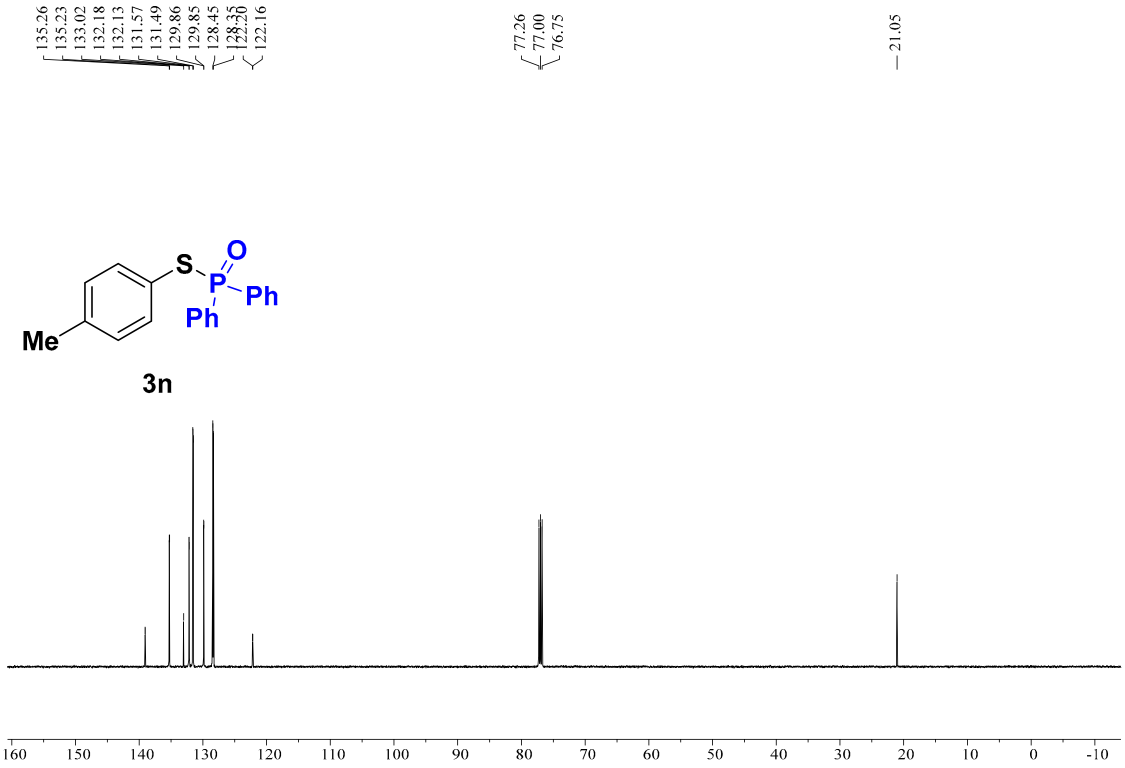
135.26
135.23
133.02
132.18
132.13
131.57
131.49
129.86
129.85
128.45
128.35
122.20
122.16
77.26
77.00
76.75
21.05
Me
S
P
O
Ph
Ph
3n
160
150
140
130
120
110
100
90
80
70
60
50
40
30
20
10
0
-10

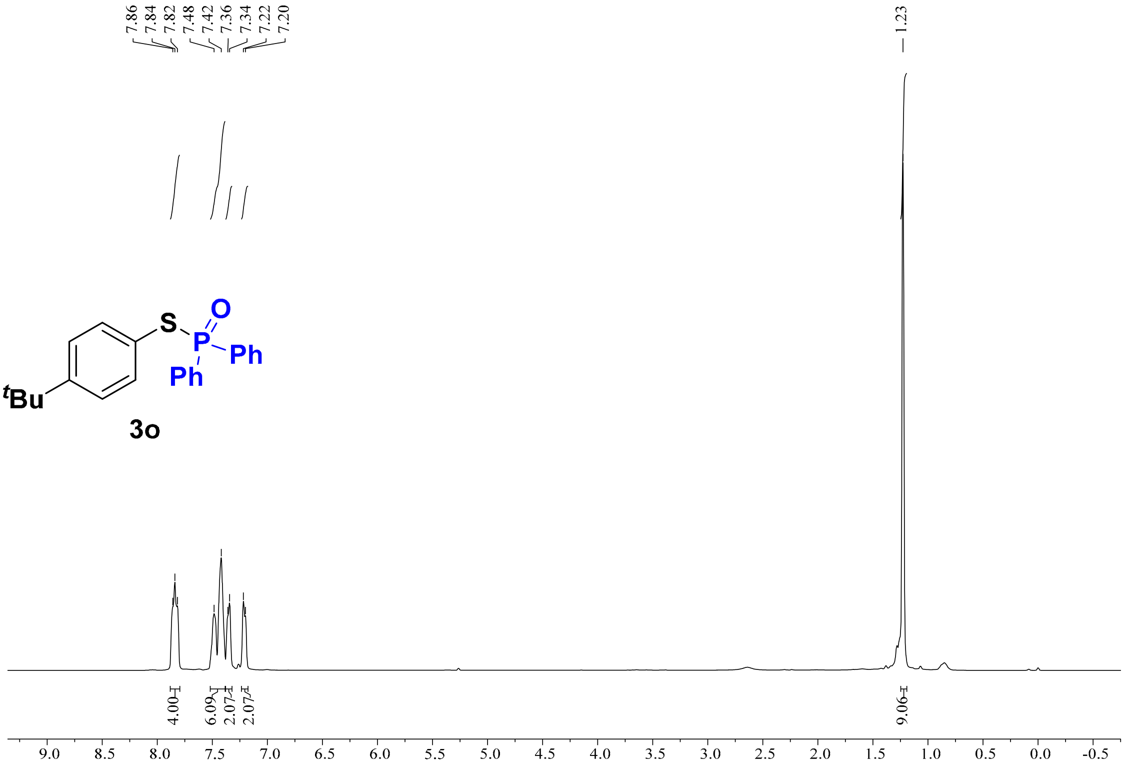
7.86
7.84
7.82
7.48
7.42
7.36
7.34
7.22
7.20
1.23
S
O
P
Ph
Ph
tBu
3o
4.00
6.09
2.07
2.07
9.06
9.0 8.5 8.0 7.5 7.0 6.5 6.0 5.5 5.0 4.5 4.0 3.5 3.0 2.5 2.0 1.5 1.0 0.5 0.0 -0.5

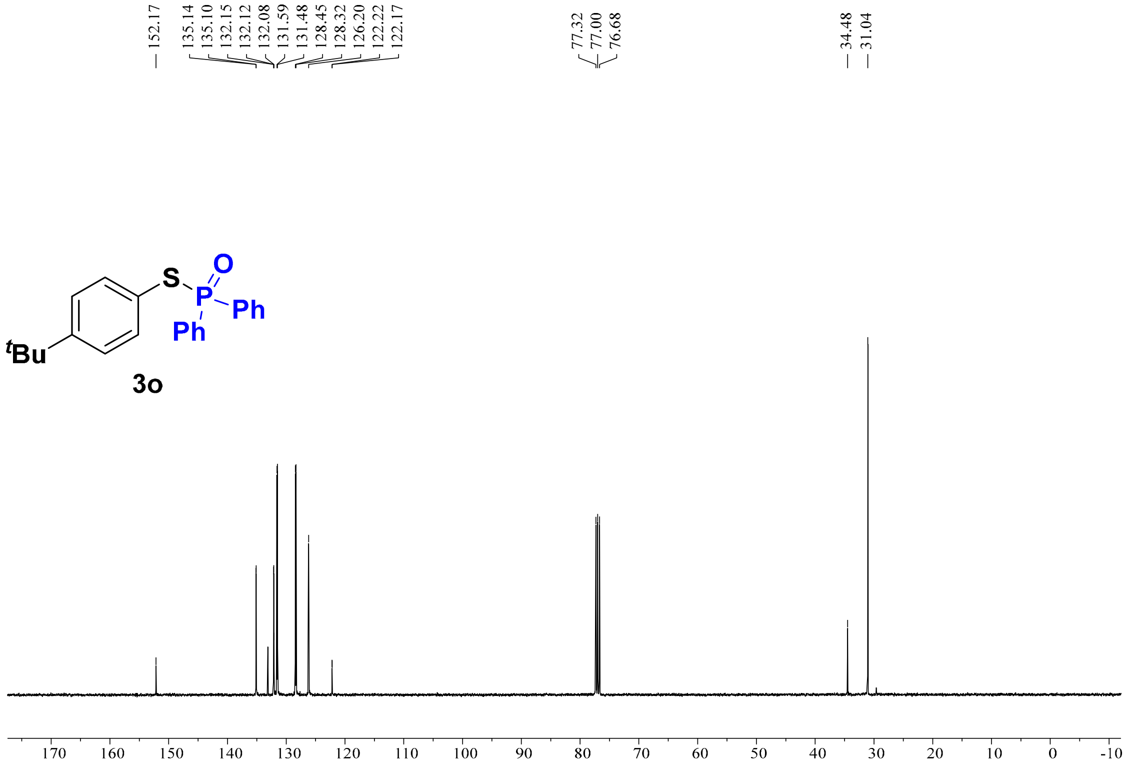
152.17
135.14
135.10
132.15
132.12
132.08
131.59
131.48
128.45
128.32
126.20
122.22
122.17
77.32
77.00
76.68
34.48
31.04
S
O
P
Ph
Ph
tBu
3o
170 160 150 140 130 120 110 100 90 80 70 60 50 40 30 20 10 0 -10

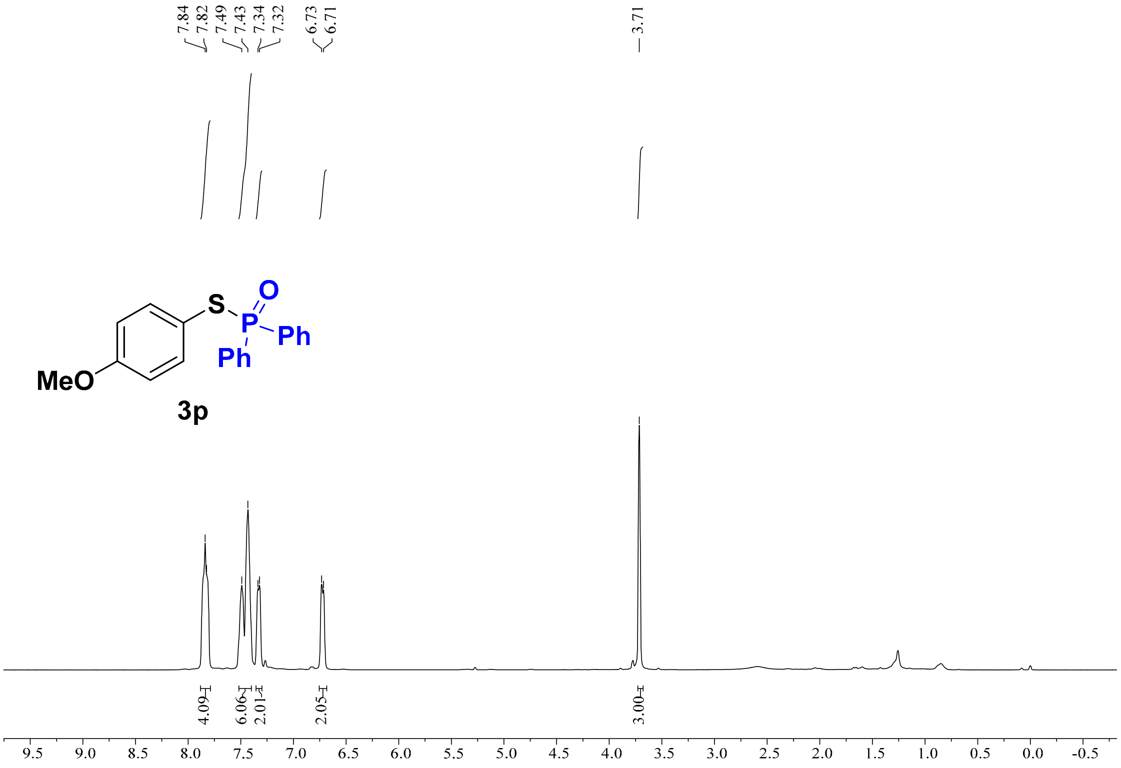
7.84
7.82
7.49
7.43
7.34
7.32
6.73
6.71
3.71
MeO
S
P
O
Ph
Ph
3p
4.09
6.06
2.01
2.05
3.00
9.5 9.0 8.5 8.0 7.5 7.0 6.5 6.0 5.5 5.0 4.5 4.0 3.5 3.0 2.5 2.0 1.5 1.0 0.5 0.0 -0.5

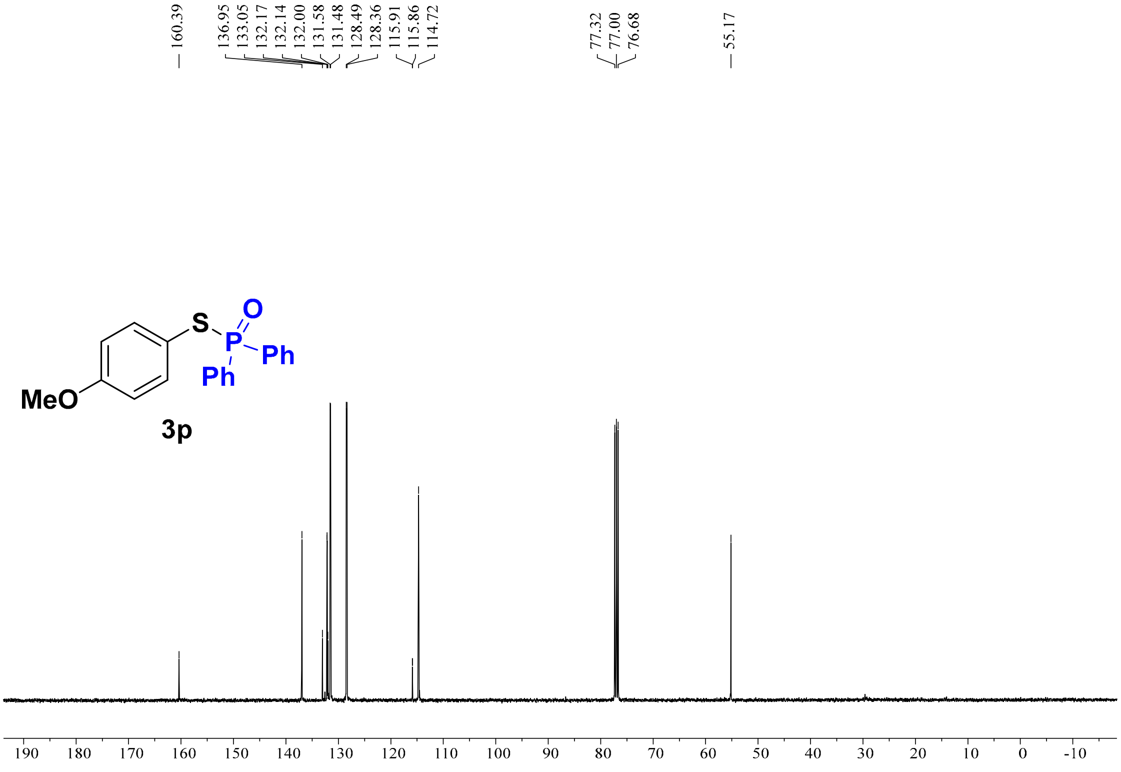
160.39
136.95
133.05
132.17
132.14
132.00
131.58
131.48
128.49
128.36
115.91
115.86
114.72
77.32
77.00
76.68
55.17
MeO
S
P
O
Ph
Ph
3p
190 180 170 160 150 140 130 120 110 100 90 80 70 60 50 40 30 20 10 0 -10

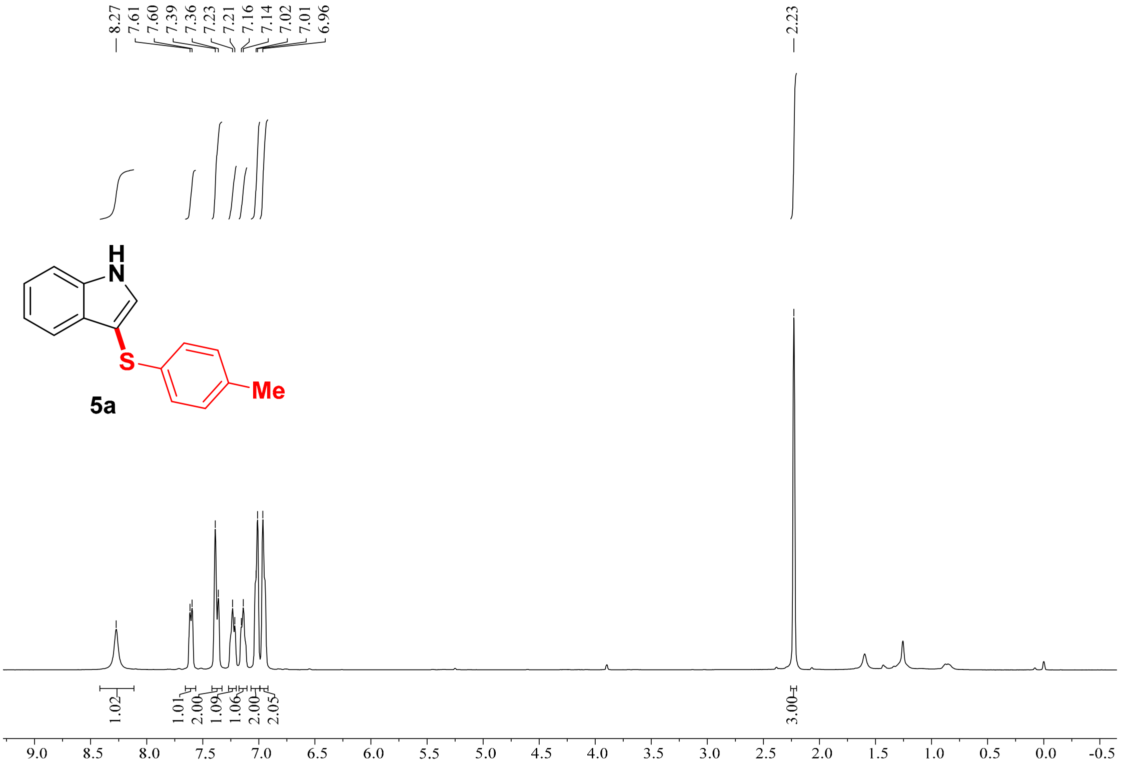
8.27
7.61
7.60
7.39
7.36
7.23
7.21
7.16
7.14
7.02
7.01
6.96
2.23
H
N
S
Me
5a
1.02
1.01
2.00
1.09
1.06
2.00
2.05
3.00
9.0
8.5
8.0
7.5
7.0
6.5
6.0
5.5
5.0
4.5
4.0
3.5
3.0
2.5
2.0
1.5
1.0
0.5
0.0
-0.5

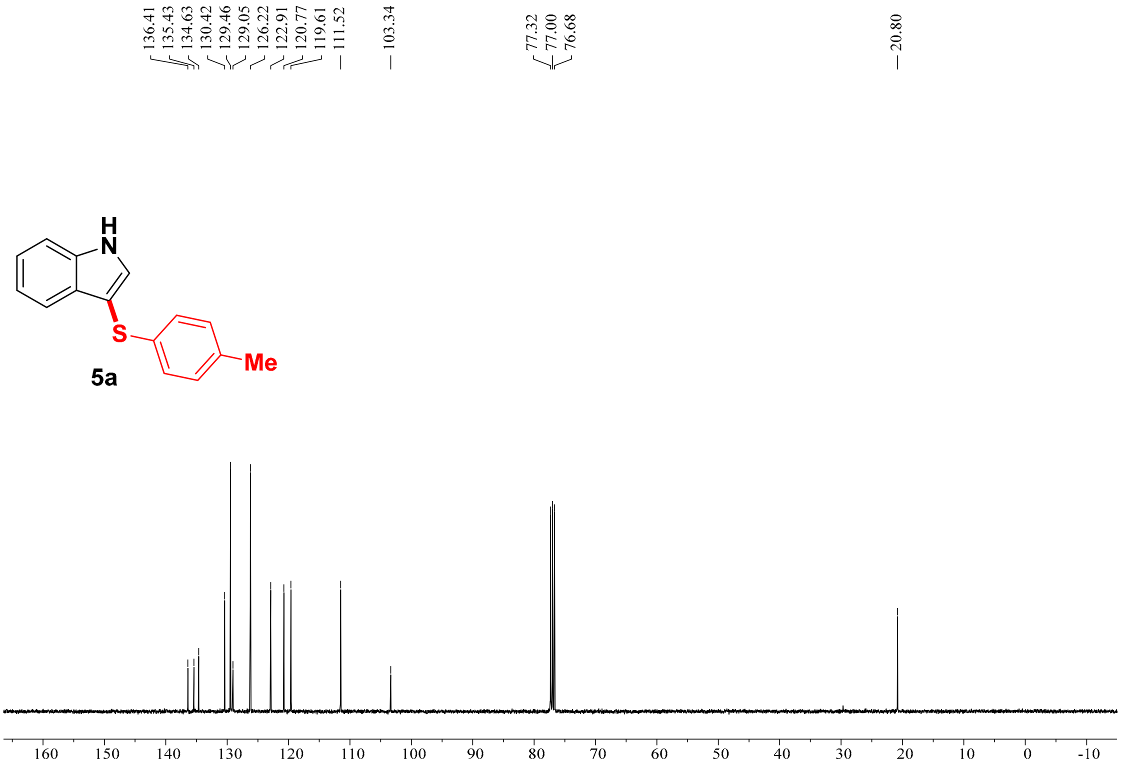
136.41
135.43
134.63
130.42
129.46
129.05
126.22
122.91
120.77
119.61
111.52
103.34
77.32
77.00
76.68
20.80
H
N
S
Me
5a
160
150
140
130
120
110
100
90
80
70
60
50
40
30
20
10
0
-10

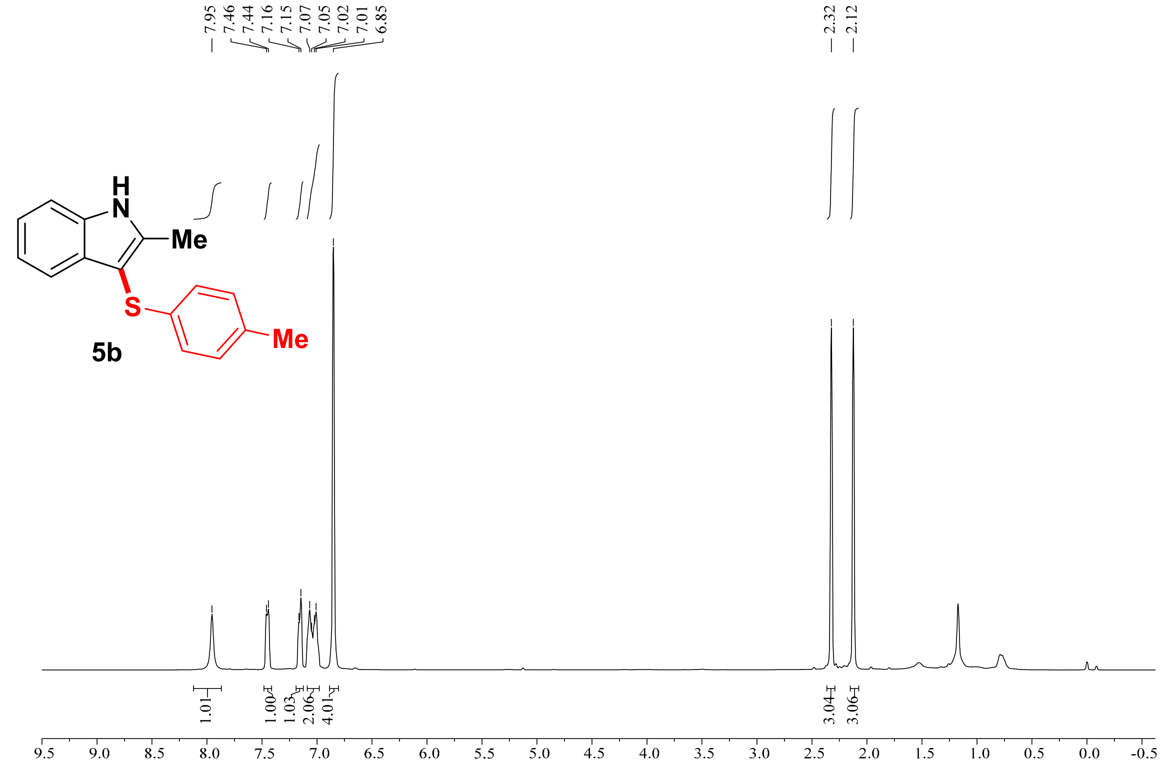
5b

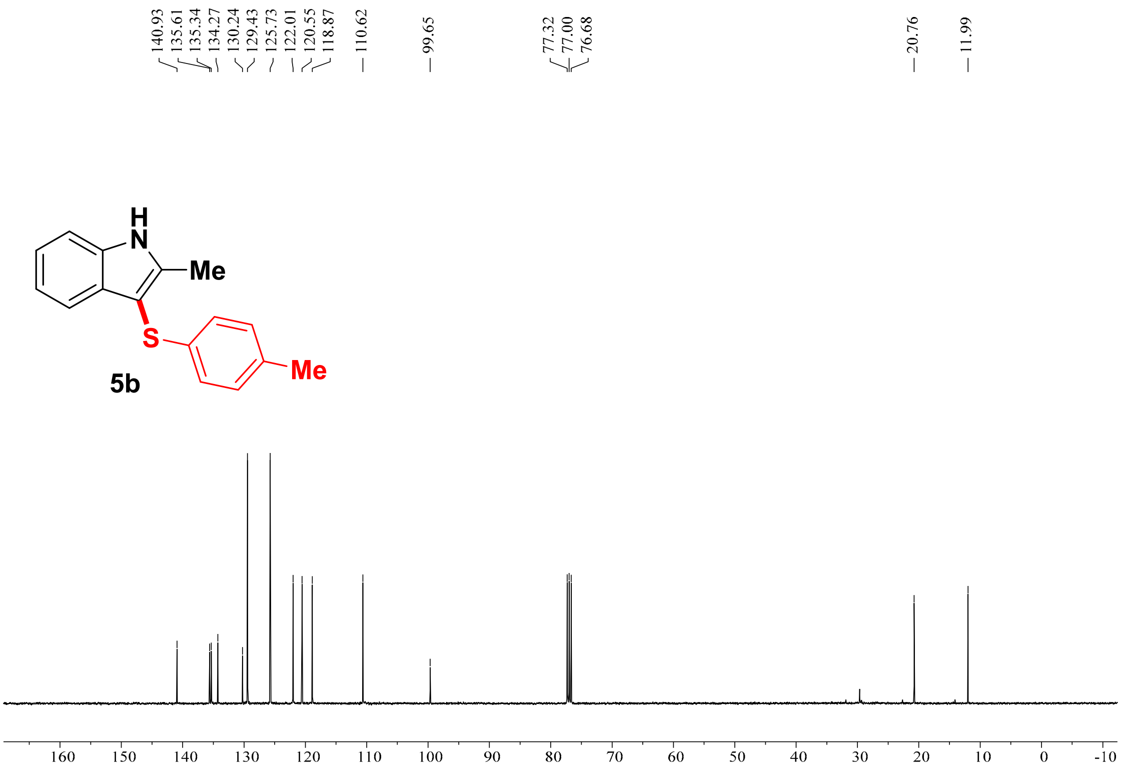
5b

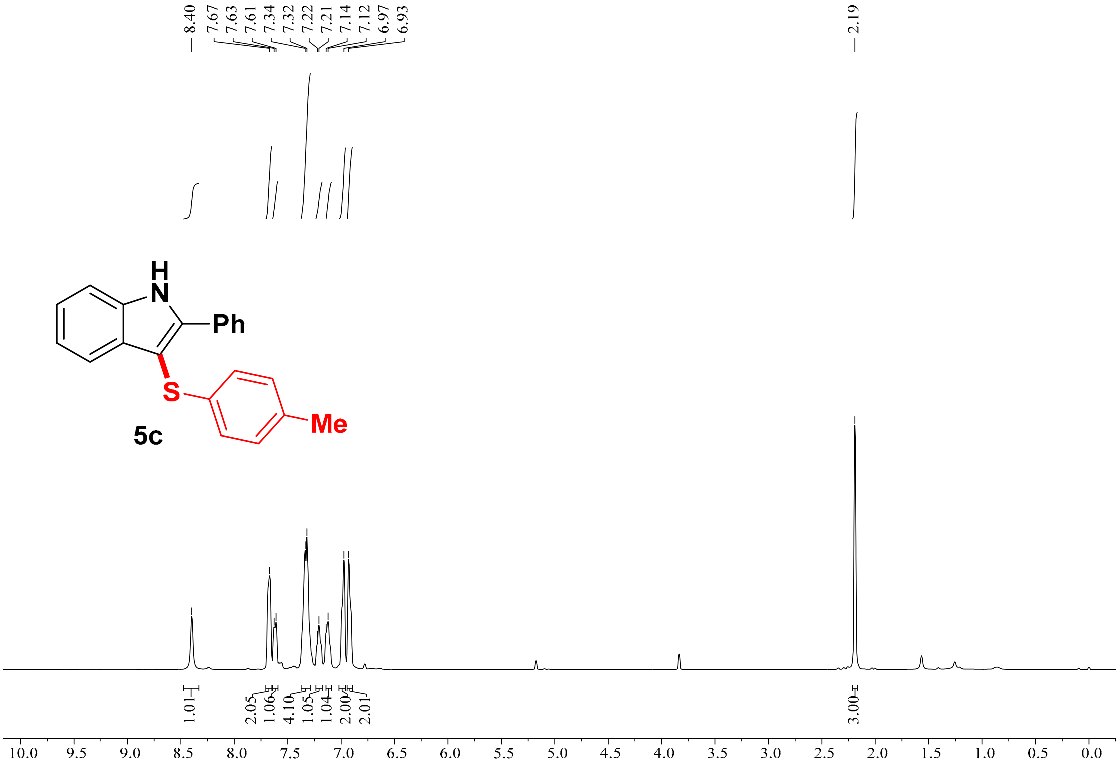
8.40
7.67
7.63
7.61
7.34
7.32
7.22
7.21
7.14
7.12
6.97
6.93
2.19
H
N
Ph
S
Me
5c
1.01
2.05
1.06
4.10
1.05
1.04
2.00
2.01
3.00
10.0 9.5 9.0 8.5 8.0 7.5 7.0 6.5 6.0 5.5 5.0 4.5 4.0 3.5 3.0 2.5 2.0 1.5 1.0 0.5 0.0

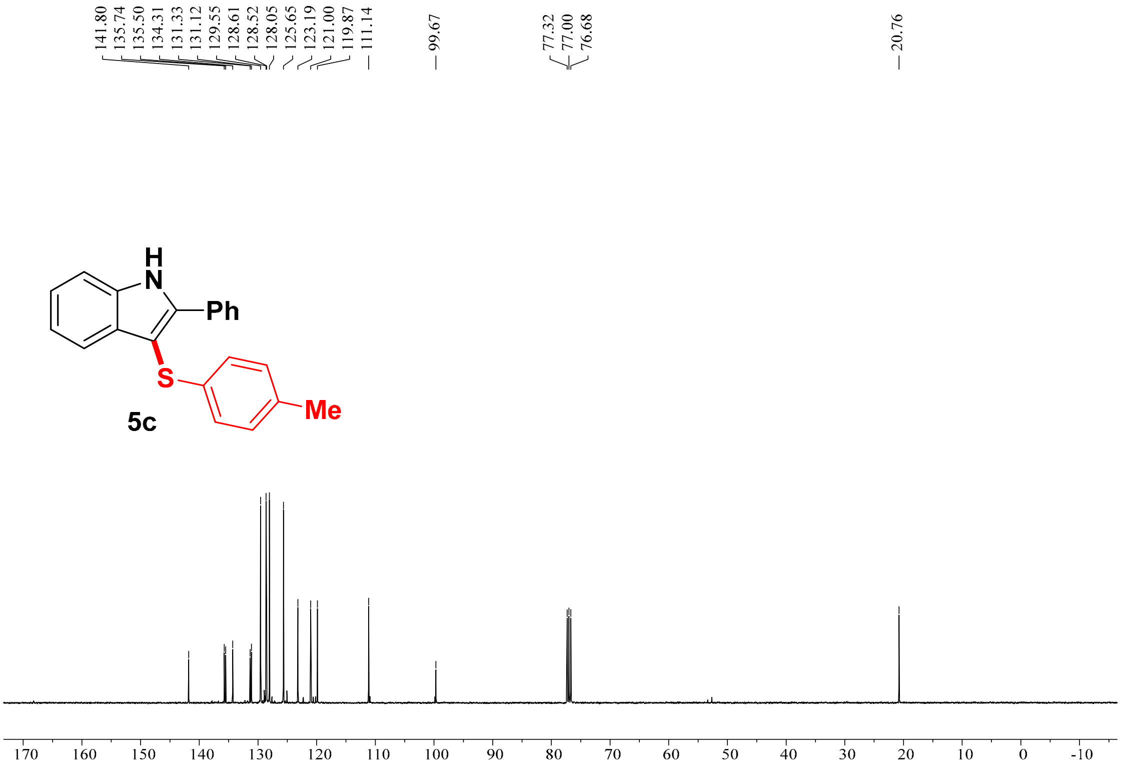
141.80
135.74
135.50
134.31
131.33
131.12
129.55
128.61
128.52
128.05
125.65
123.19
121.00
119.87
111.14
99.67
77.32
77.00
76.68
20.76
H
N
Ph
S
Me
5c
170 160 150 140 130 120 110 100 90 80 70 60 50 40 30 20 10 0 -10

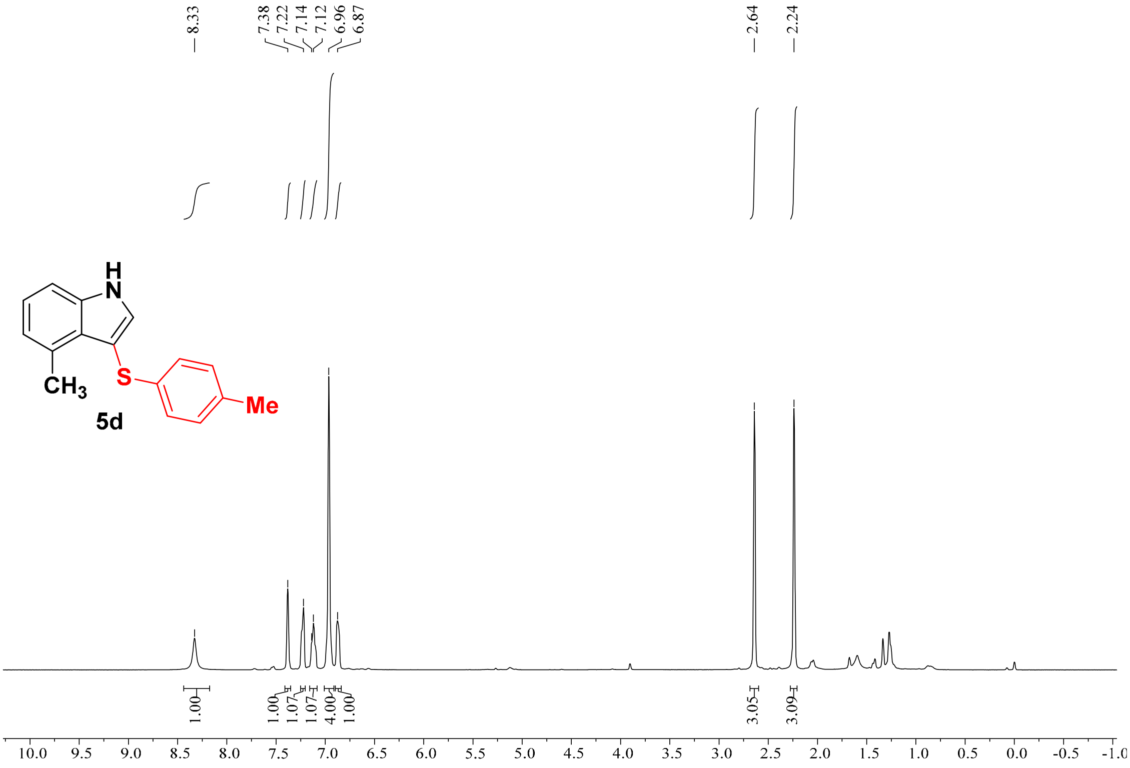
8.33
7.38
7.22
7.14
7.12
6.96
6.87
2.64
2.24
H
N
S
CH3
Me
5d
1.00
1.00
1.07
1.07
4.00
1.00
3.05
3.09
10.0 9.5 9.0 8.5 8.0 7.5 7.0 6.5 6.0 5.5 5.0 4.5 4.0 3.5 3.0 2.5 2.0 1.5 1.0 0.5 0.0 -0.5 -1.0

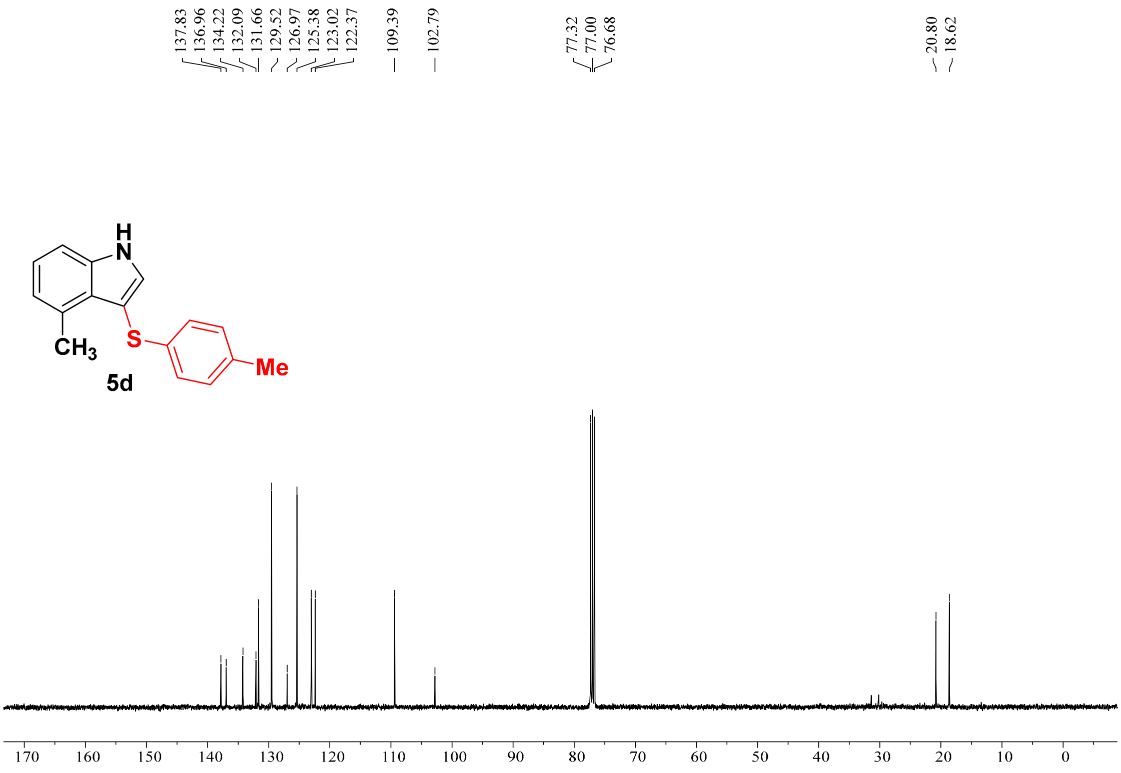
137.83
136.96
134.22
132.09
131.66
129.52
126.97
125.38
123.02
122.37
109.39
102.79
77.32
77.00
76.68
20.80
18.62
H
N
S
CH3
Me
5d
170 160 150 140 130 120 110 100 90 80 70 60 50 40 30 20 10 0

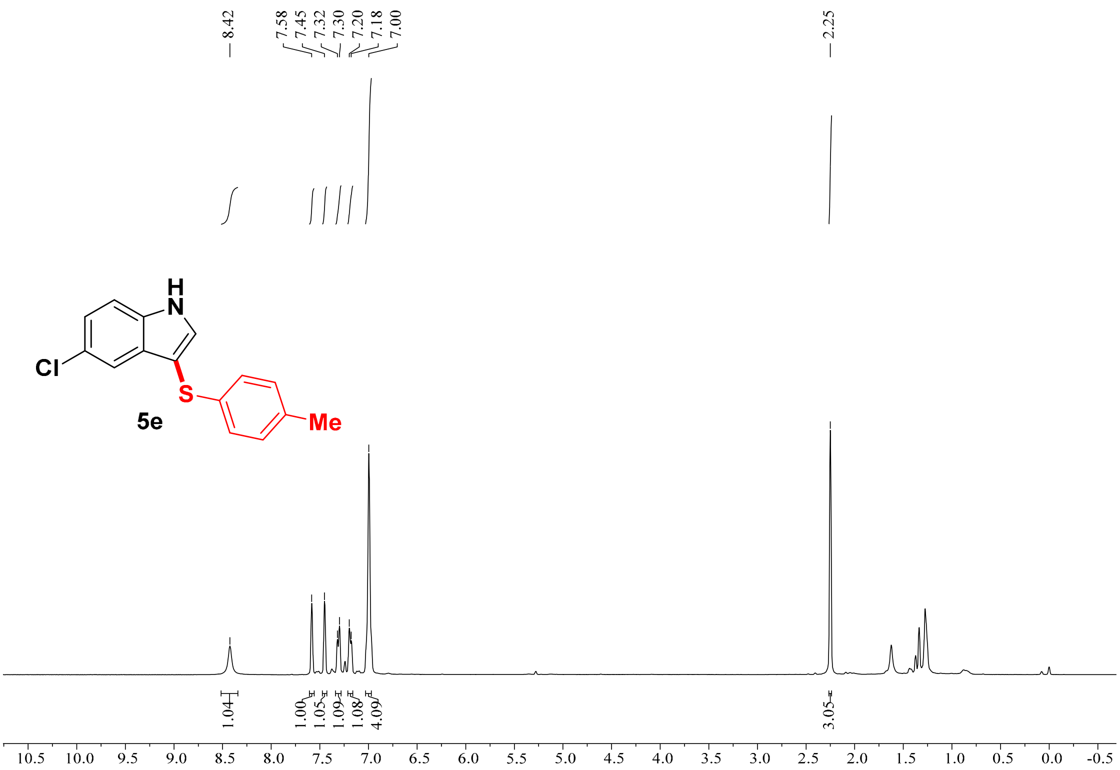
8.42
7.58
7.45
7.32
7.30
7.20
7.18
7.00
2.25
H
N
Cl
S
Me
5e
1.04
1.00
1.05
1.09
1.08
4.09
3.05
10.5 10.0 9.5 9.0 8.5 8.0 7.5 7.0 6.5 6.0 5.5 5.0 4.5 4.0 3.5 3.0 2.5 2.0 1.5 1.0 0.5 0.0 -0.5

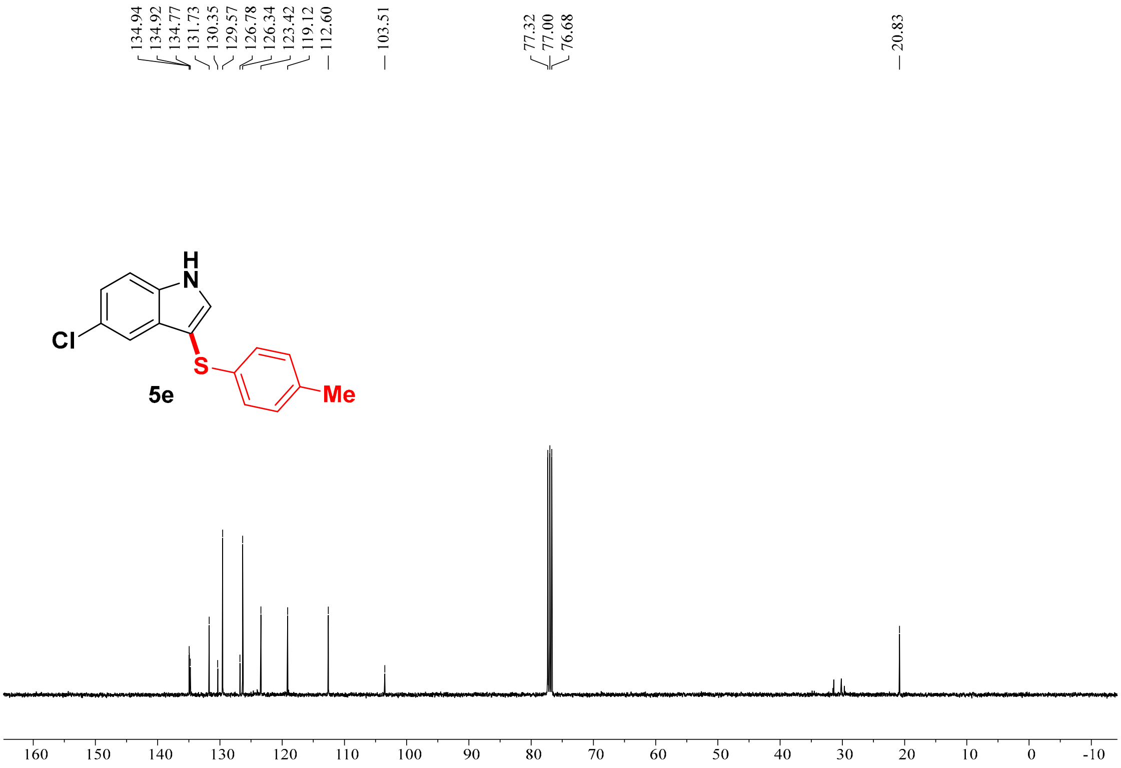
134.94
134.92
134.77
131.73
130.35
129.57
126.78
126.34
123.42
119.12
112.60
103.51
77.32
77.00
76.68
20.83
H
N
Cl
S
Me
5e
160 150 140 130 120 110 100 90 80 70 60 50 40 30 20 10 0 -10

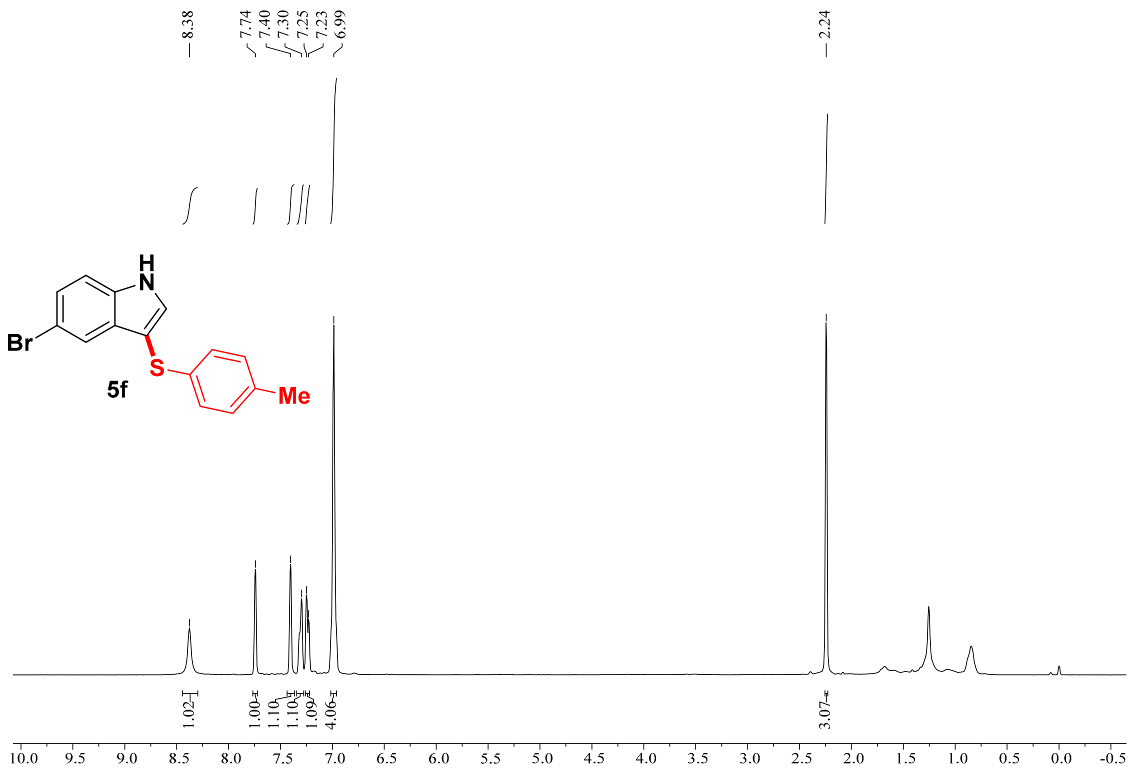
8.38
7.74
7.40
7.30
7.25
7.23
6.99
2.24
Br
H
N
S
Me
5f
1.02
1.00
1.10
1.10
1.09
4.06
3.07
10.0
9.5
9.0
8.5
8.0
7.5
7.0
6.5
6.0
5.5
5.0
4.5
4.0
3.5
3.0
2.5
2.0
1.5
1.0
0.5
0.0
-0.5

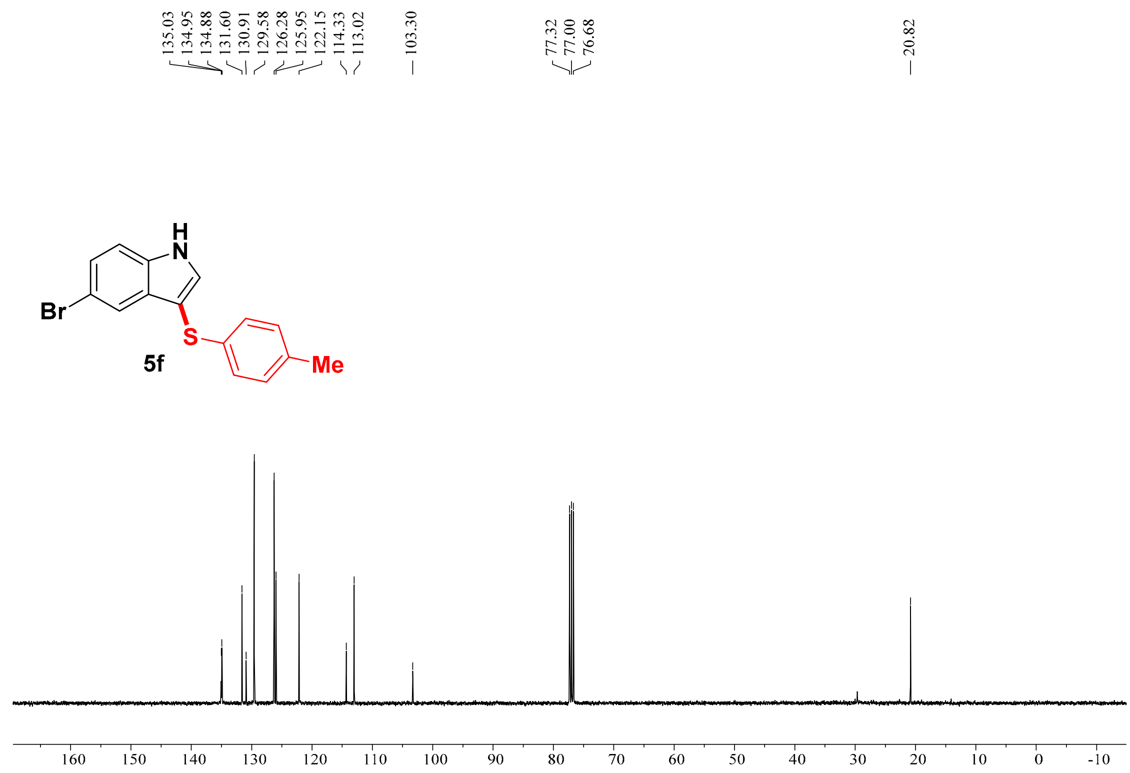
135.03
134.95
134.88
131.60
130.91
129.58
126.28
125.95
122.15
114.33
113.02
103.30
77.32
77.00
76.68
20.82
Br
H
N
S
Me
5f
160
150
140
130
120
110
100
90
80
70
60
50
40
30
20
10
0
-10

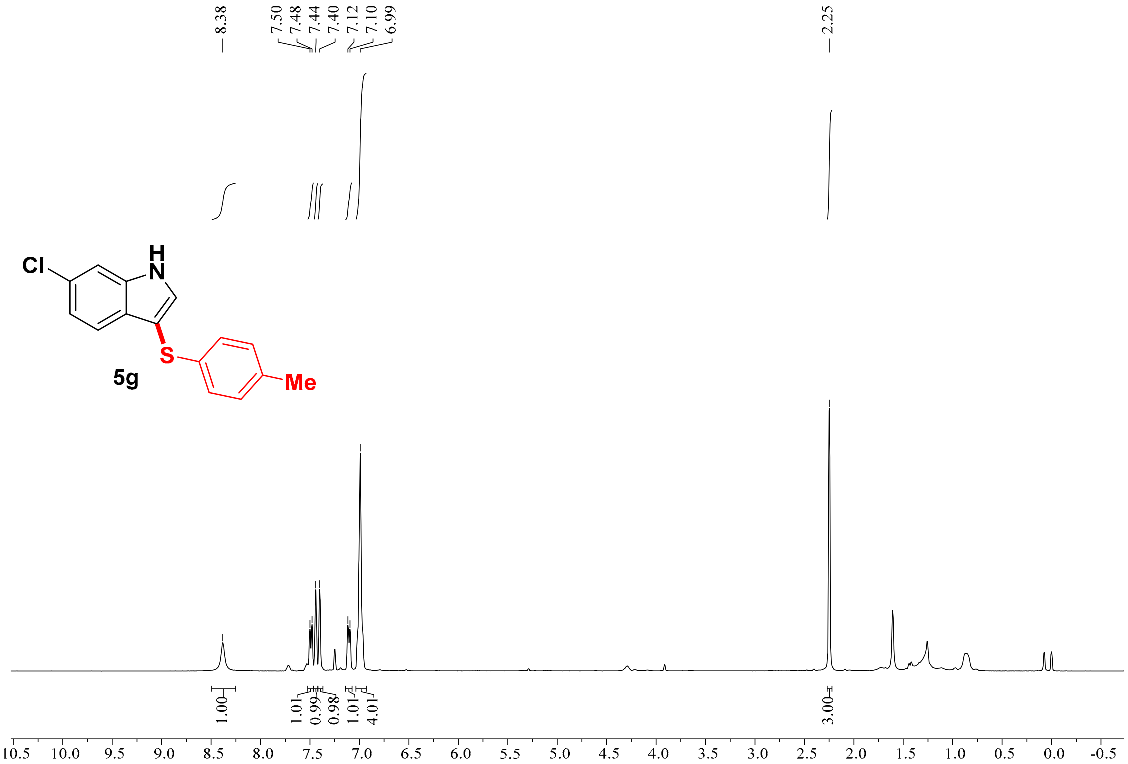
8.38
7.50
7.48
7.44
7.40
7.12
7.10
6.99
2.25
Cl
H
N
S
5g
Me
1.00
1.01
0.99
0.98
1.01
4.01
3.00
10.5
10.0
9.5
9.0
8.5
8.0
7.5
7.0
6.5
6.0
5.5
5.0
4.5
4.0
3.5
3.0
2.5
2.0
1.5
1.0
0.5
0.0
-0.5

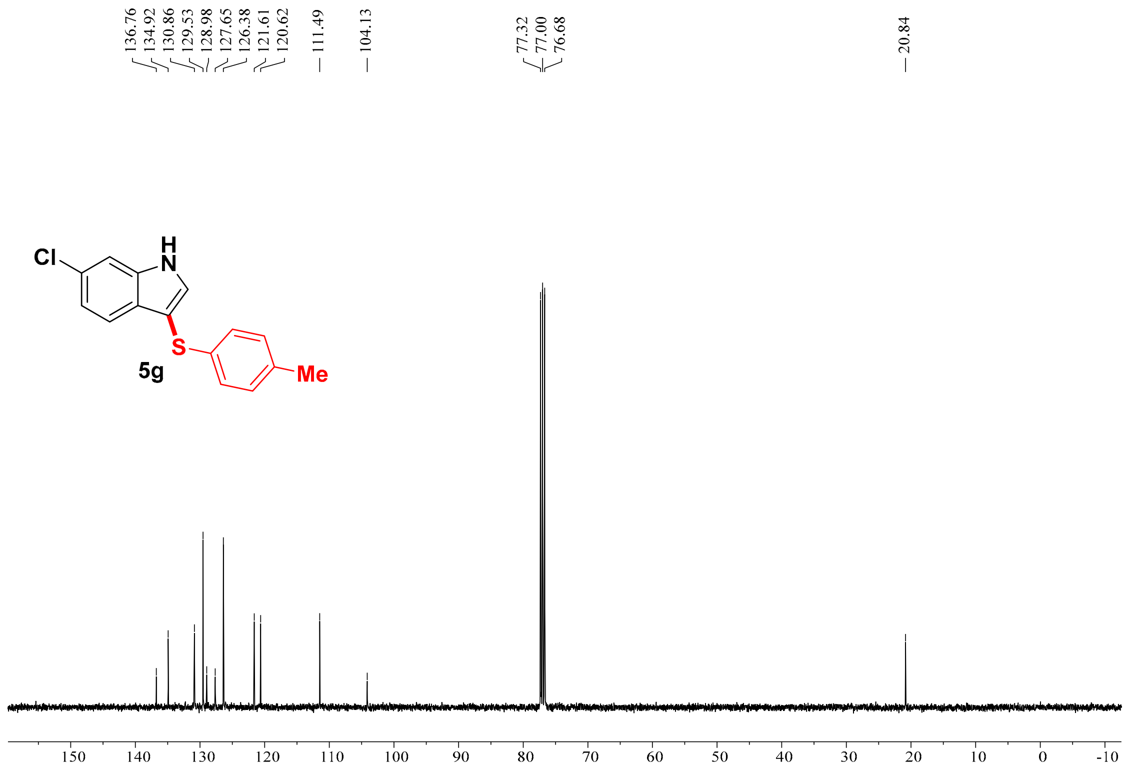
136.76
134.92
130.86
129.53
128.98
127.65
126.38
121.61
120.62
111.49
104.13
77.32
77.00
76.68
20.84
Cl
H
N
S
5g
Me
150
140
130
120
110
100
90
80
70
60
50
40
30
20
10
0
-10

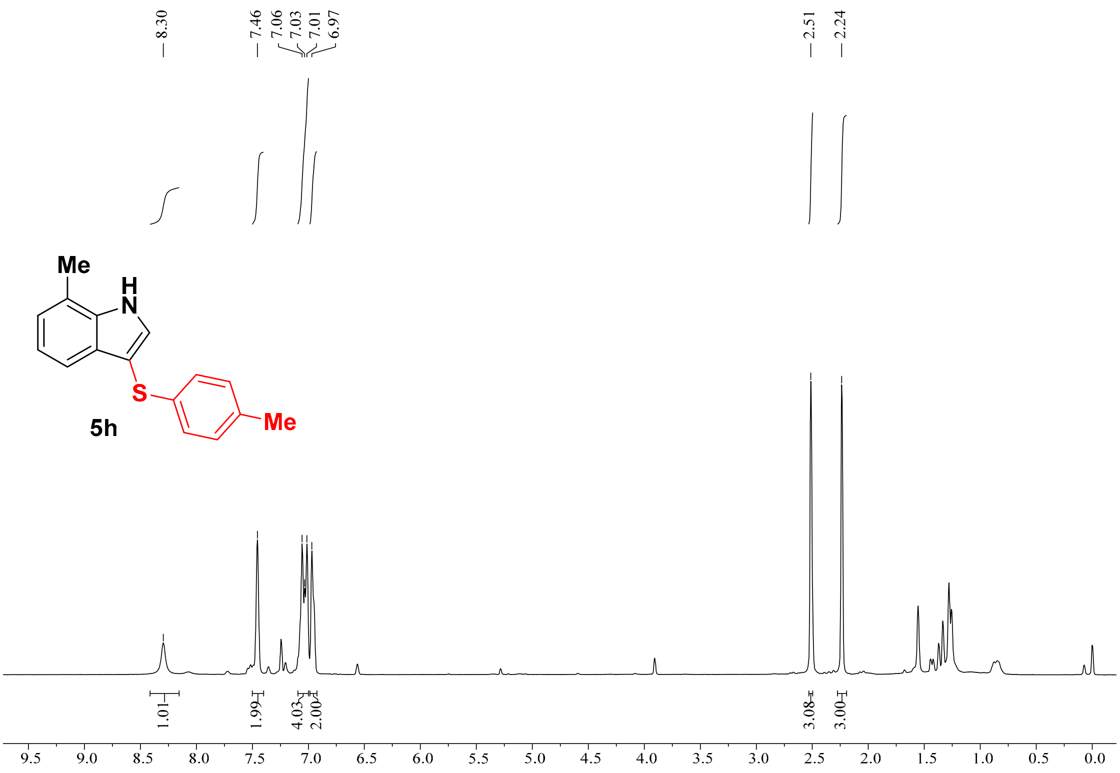
8.30
7.46
7.06
7.03
7.01
6.97
2.51
2.24
Me
H
N
S
Me
5h
1.01
1.99
4.03
2.00
3.08
3.00
9.5
9.0
8.5
8.0
7.5
7.0
6.5
6.0
5.5
5.0
4.5
4.0
3.5
3.0
2.5
2.0
1.5
1.0
0.5
0.0

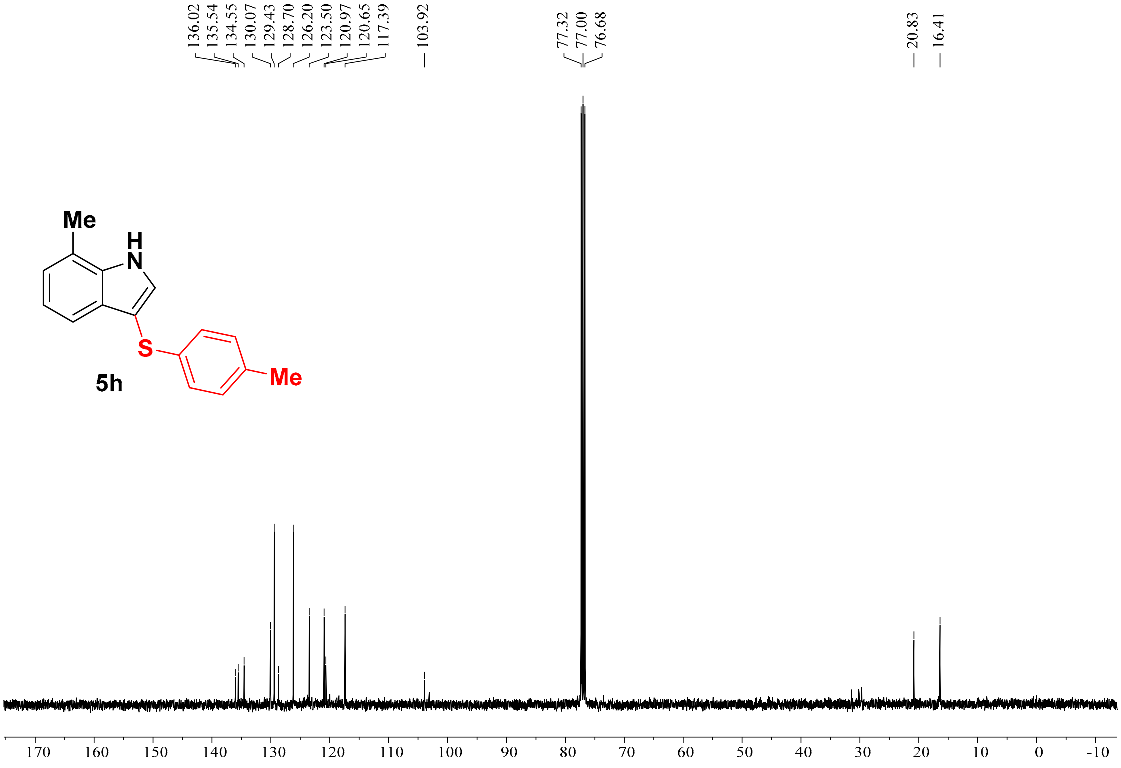
136.02
135.54
134.55
130.07
129.43
128.70
126.20
123.50
120.97
120.65
117.39
103.92
77.32
77.00
76.68
20.83
16.41
Me
H
N
S
Me
5h
170
160
150
140
130
120
110
100
90
80
70
60
50
40
30
20
10
0
-10

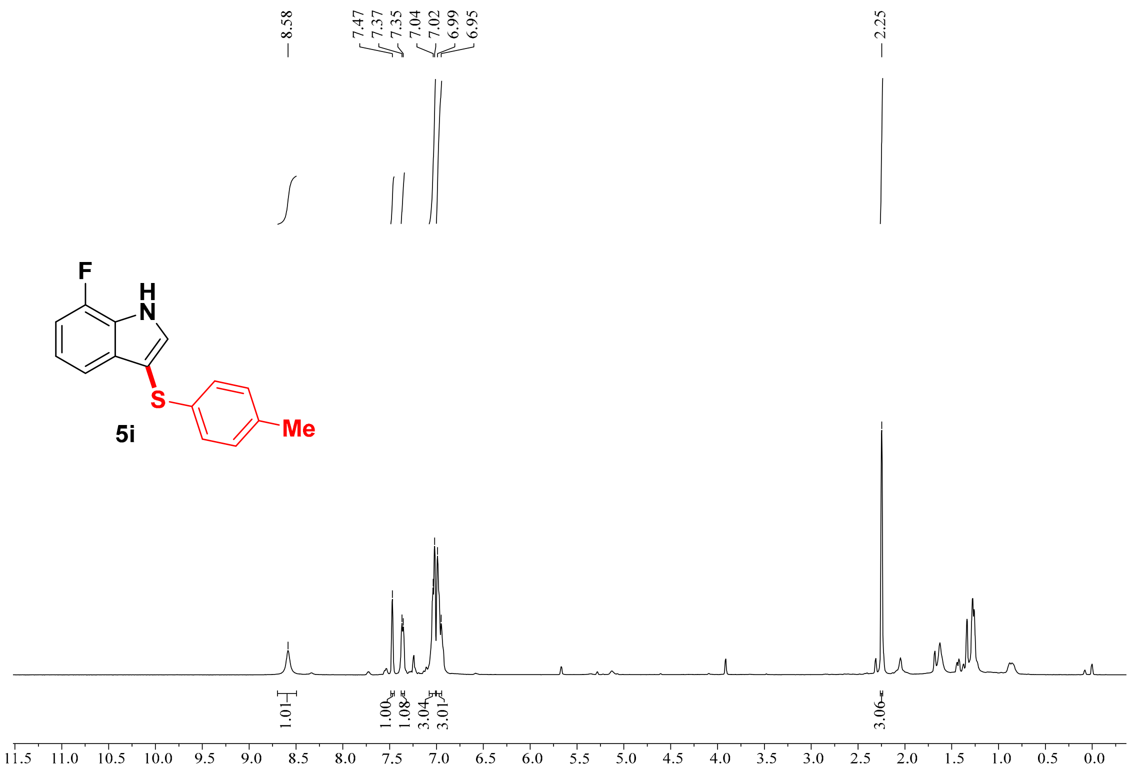
8.58
7.47
7.37
7.35
7.04
7.02
6.99
6.95
2.25
F
H
N
S
Me
5i
1.01
1.00
1.08
3.04
3.01
3.06
11.5 11.0 10.5 10.0 9.5 9.0 8.5 8.0 7.5 7.0 6.5 6.0 5.5 5.0 4.5 4.0 3.5 3.0 2.5 2.0 1.5 1.0 0.5 0.0

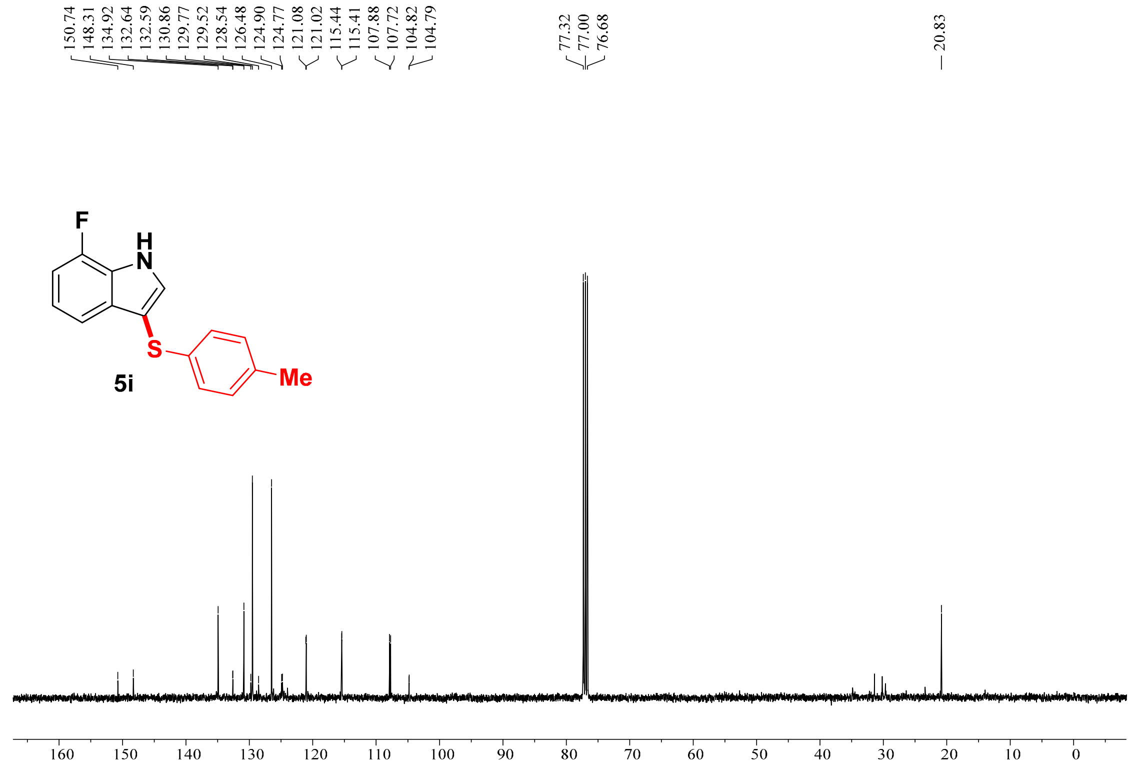
150.74
148.31
134.92
132.64
132.59
130.86
129.77
129.52
128.54
126.48
124.90
124.77
121.08
121.02
115.44
115.41
107.88
107.72
104.82
104.79
77.32
77.00
76.68
20.83
F
H
N
S
Me
5i
160 150 140 130 120 110 100 90 80 70 60 50 40 30 20 10 0

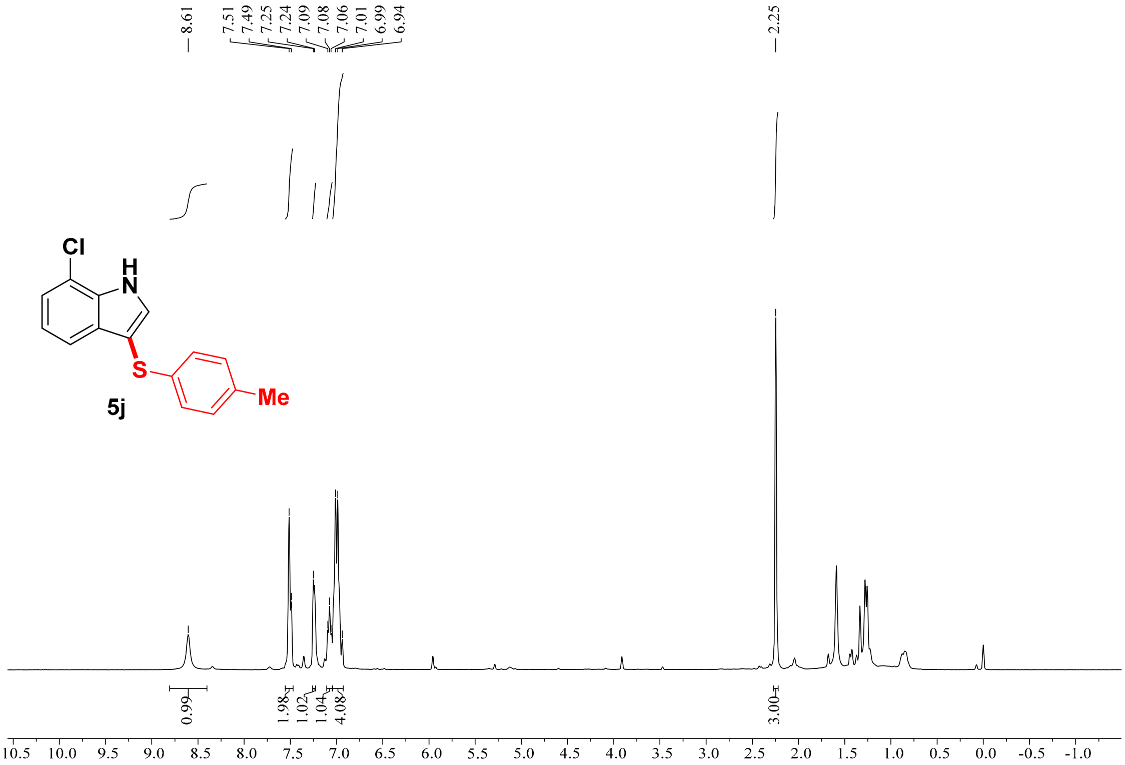
8.61
7.51
7.49
7.25
7.24
7.09
7.08
7.06
7.01
6.99
6.94
2.25
Cl
H
N
S
Me
5j
0.99
1.98
1.02
1.04
4.08
3.00
10.5 10.0 9.5 9.0 8.5 8.0 7.5 7.0 6.5 6.0 5.5 5.0 4.5 4.0 3.5 3.0 2.5 2.0 1.5 1.0 0.5 0.0 -0.5 -1.0

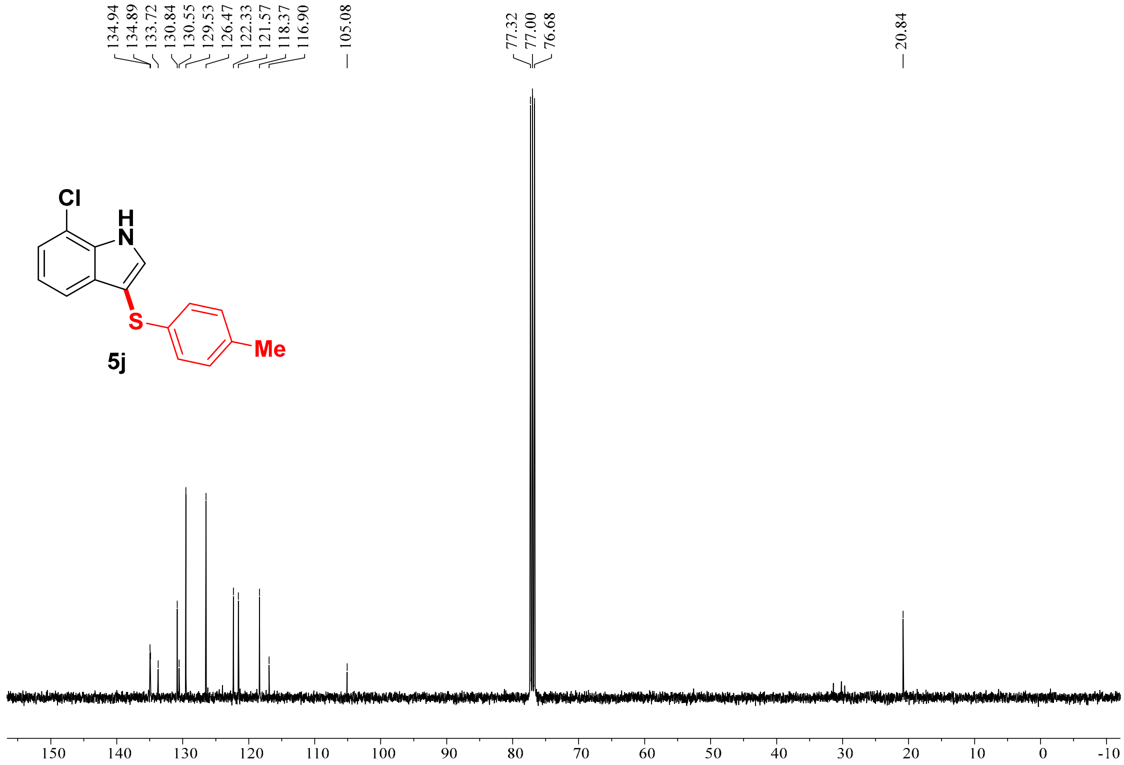
134.94
134.89
133.72
130.84
130.55
129.53
126.47
122.33
121.57
118.37
116.90
105.08
77.32
77.00
76.68
20.84
Cl
H
N
S
Me
5j
150 140 130 120 110 100 90 80 70 60 50 40 30 20 10 0 -10

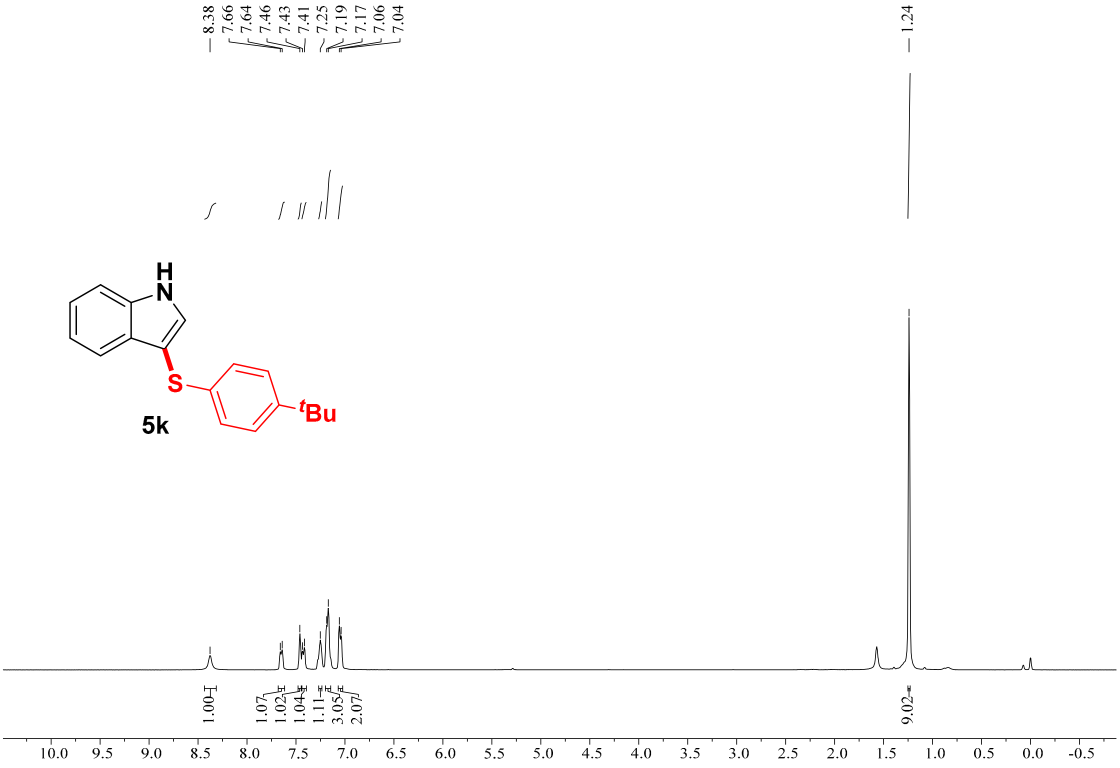
8.38
7.66
7.64
7.46
7.43
7.41
7.25
7.19
7.17
7.06
7.04
1.24
5k
1.00
1.07
1.02
1.04
1.11
3.05
2.07
9.02
10.0 9.5 9.0 8.5 8.0 7.5 7.0 6.5 6.0 5.5 5.0 4.5 4.0 3.5 3.0 2.5 2.0 1.5 1.0 0.5 0.0 -0.5

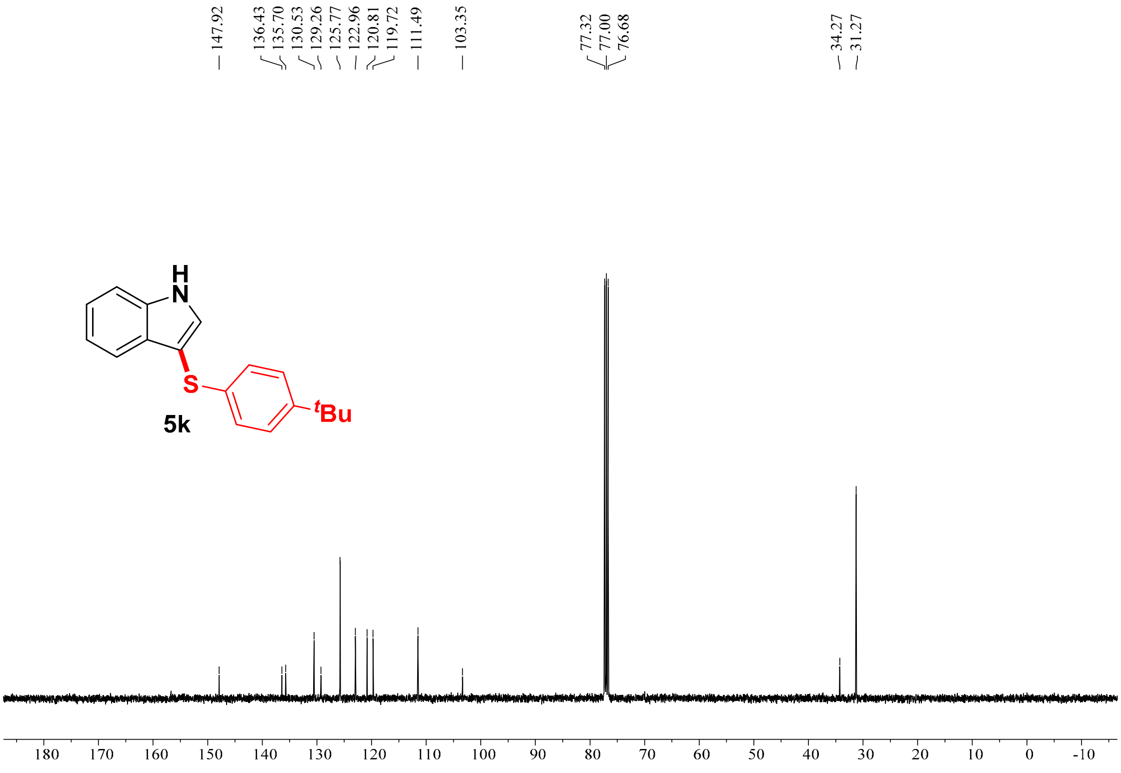
147.92
136.43
135.70
130.53
129.26
125.77
122.96
120.81
119.72
111.49
103.35
77.32
77.00
76.68
34.27
31.27
5k
180 170 160 150 140 130 120 110 100 90 80 70 60 50 40 30 20 10 0 -10

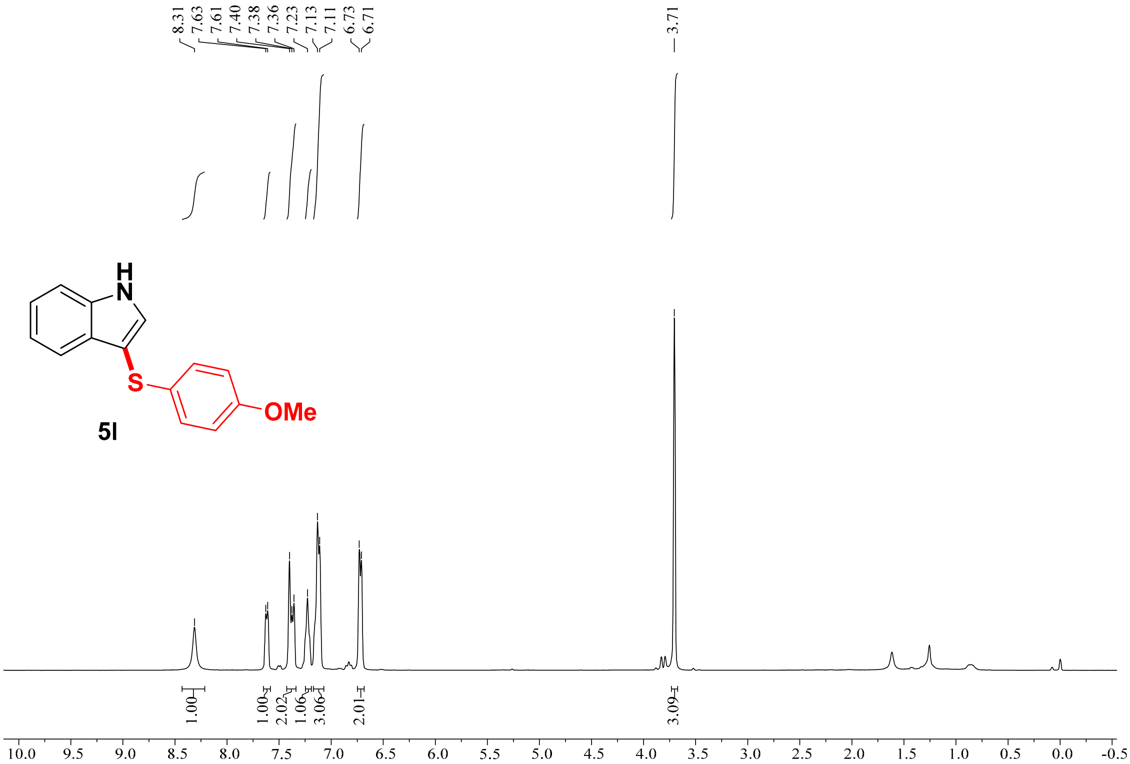
8.31
7.63
7.61
7.40
7.38
7.36
7.23
7.13
7.11
6.73
6.71
3.71
5l
OMe
1.00
1.00
2.02
1.06
3.06
2.01
3.09
10.0 9.5 9.0 8.5 8.0 7.5 7.0 6.5 6.0 5.5 5.0 4.5 4.0 3.5 3.0 2.5 2.0 1.5 1.0 0.5 0.0 -0.5

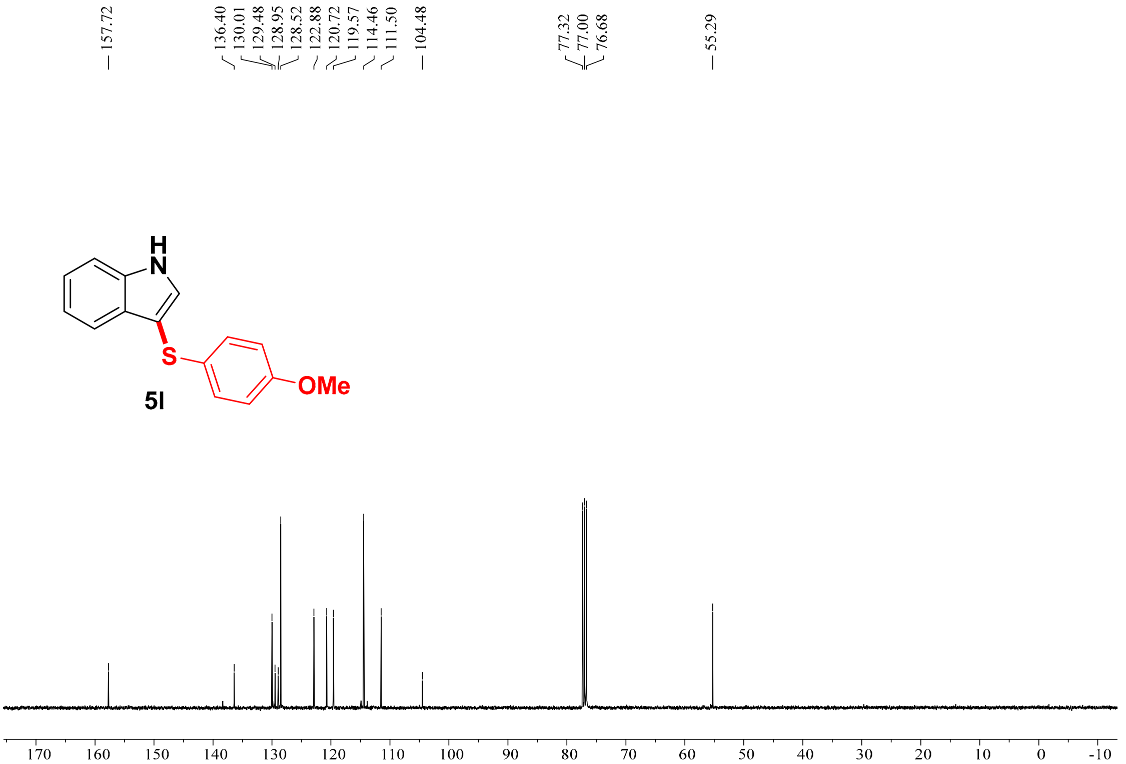
157.72
136.40
130.01
129.48
128.95
128.52
122.88
120.72
119.57
114.46
111.50
104.48
77.32
77.00
76.68
55.29
5l
OMe
170 160 150 140 130 120 110 100 90 80 70 60 50 40 30 20 10 0 -10

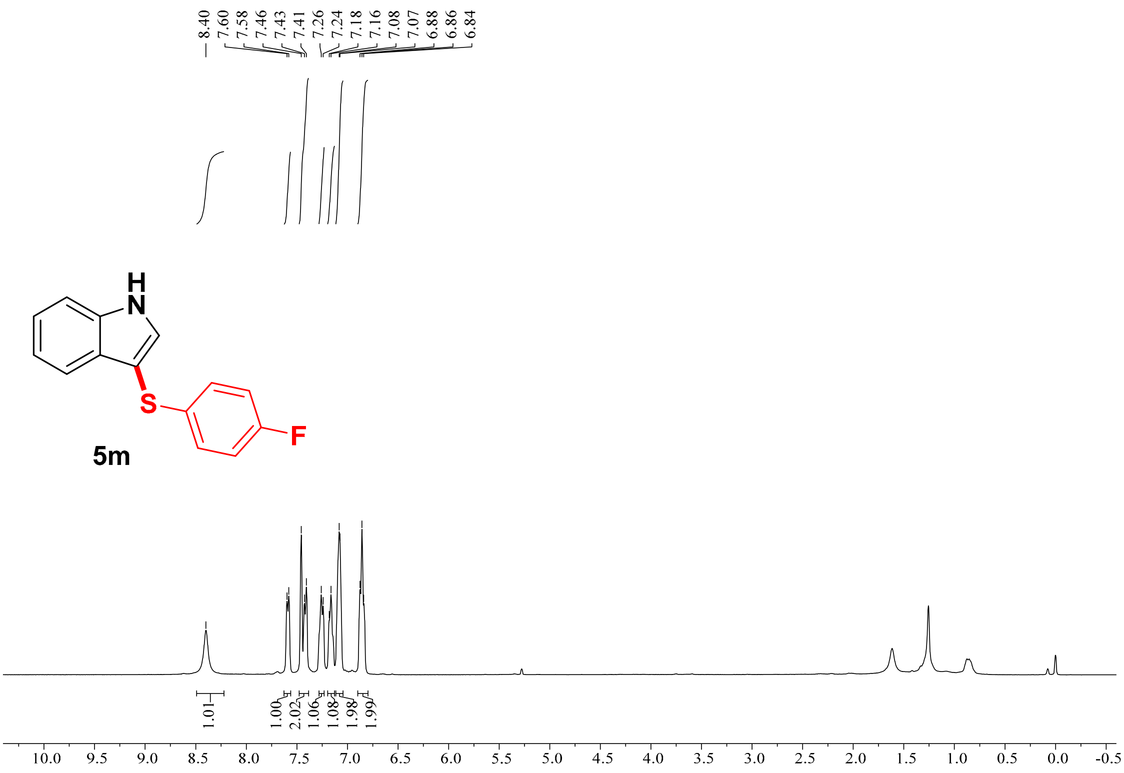
8.40
7.60
7.58
7.46
7.43
7.41
7.26
7.24
7.18
7.16
7.08
7.07
6.88
6.86
6.84
H
N
S
F
5m
1.01
1.00
2.02
1.06
1.08
1.98
1.99
10.0 9.5 9.0 8.5 8.0 7.5 7.0 6.5 6.0 5.5 5.0 4.5 4.0 3.5 3.0 2.5 2.0 1.5 1.0 0.5 0.0 -0.5

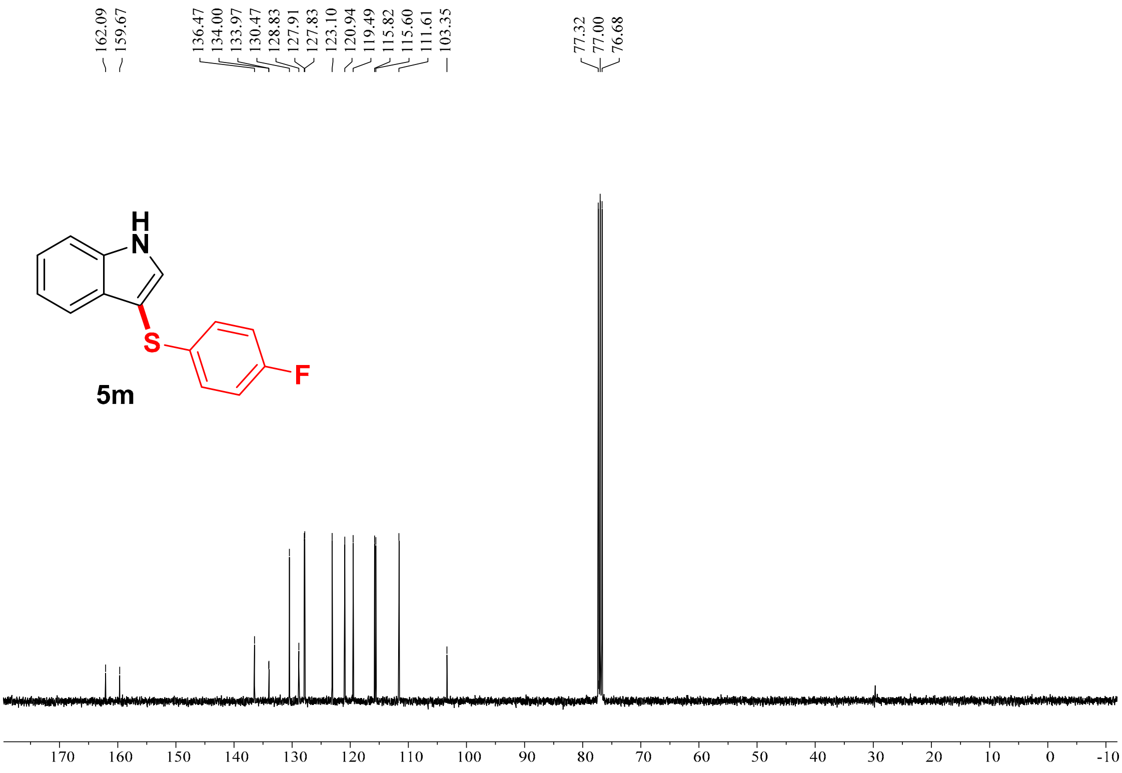
162.09
159.67
136.47
134.00
133.97
130.47
128.83
127.91
127.83
123.10
120.94
119.49
115.82
115.60
111.61
103.35
77.32
77.00
76.68
H
N
S
F
5m
170 160 150 140 130 120 110 100 90 80 70 60 50 40 30 20 10 0 -10

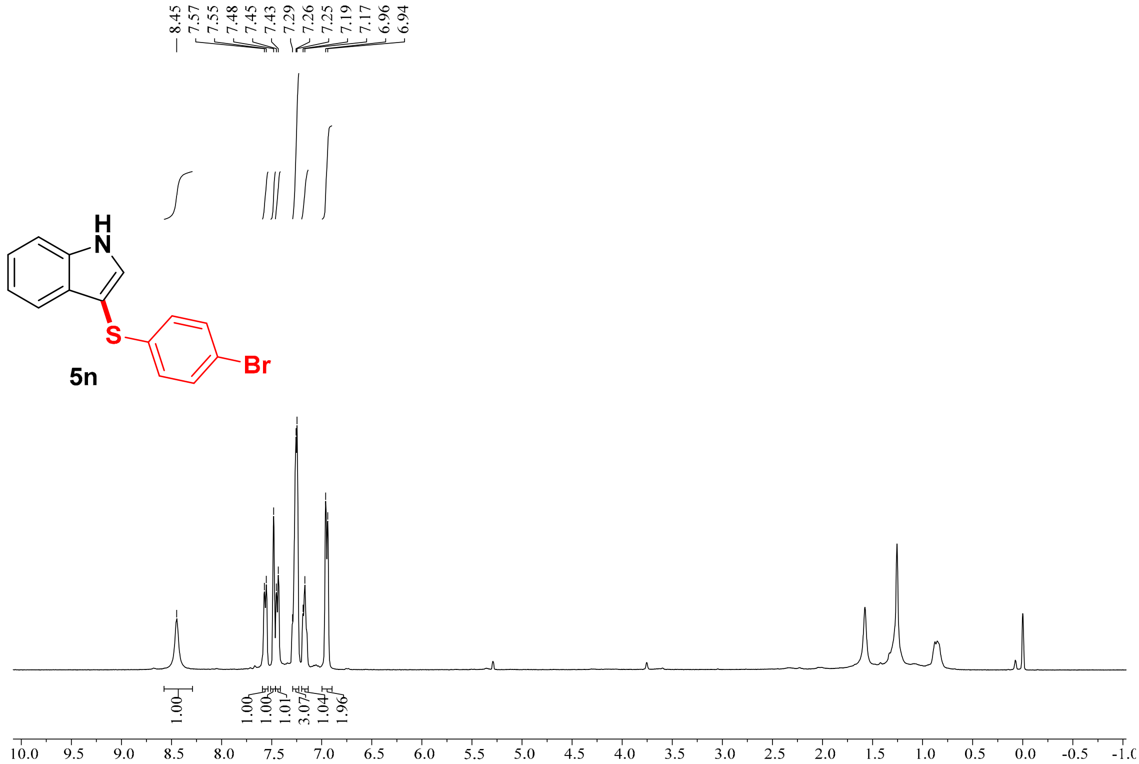
8.45
7.57
7.55
7.48
7.45
7.43
7.29
7.26
7.25
7.19
7.17
6.96
6.94
5n
Br
1.00
1.00
1.00
1.01
3.07
1.04
1.96
10.0 9.5 9.0 8.5 8.0 7.5 7.0 6.5 6.0 5.5 5.0 4.5 4.0 3.5 3.0 2.5 2.0 1.5 1.0 0.5 0.0 -0.5

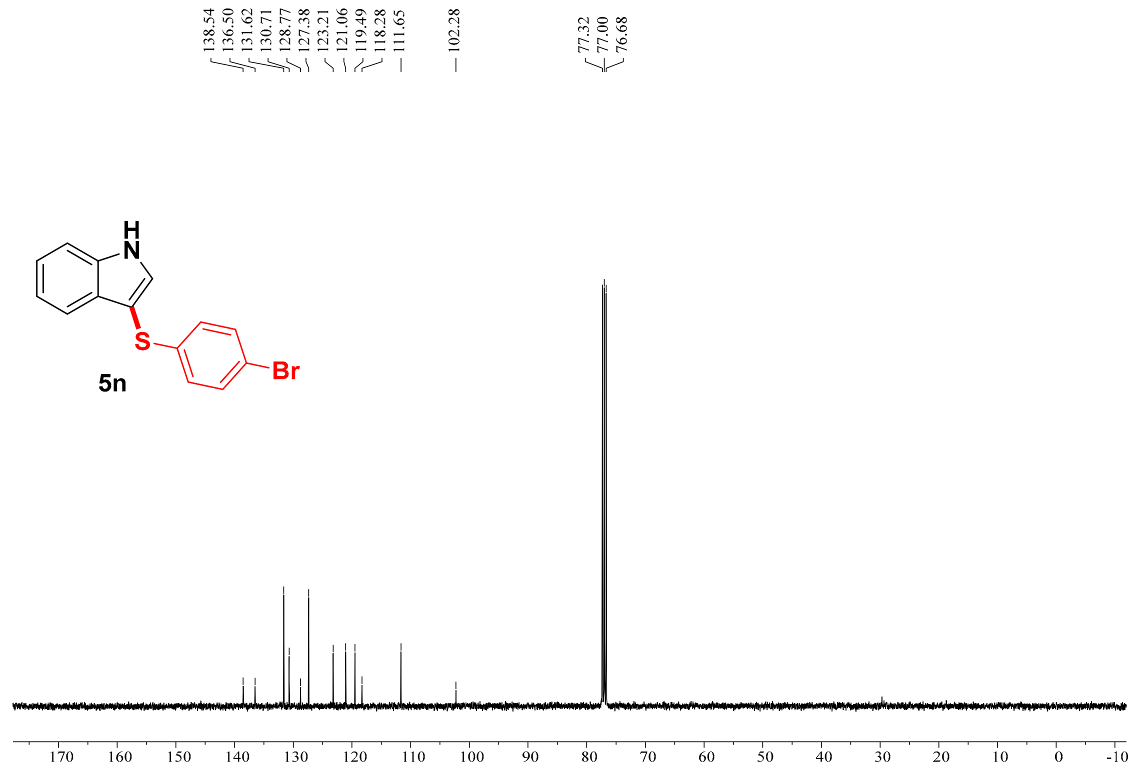
138.54
136.50
131.62
130.71
128.77
127.38
123.21
121.06
119.49
118.28
111.65
102.28
77.32
77.00
76.68
5n
Br
170 160 150 140 130 120 110 100 90 80 70 60 50 40 30 20 10 0 -10

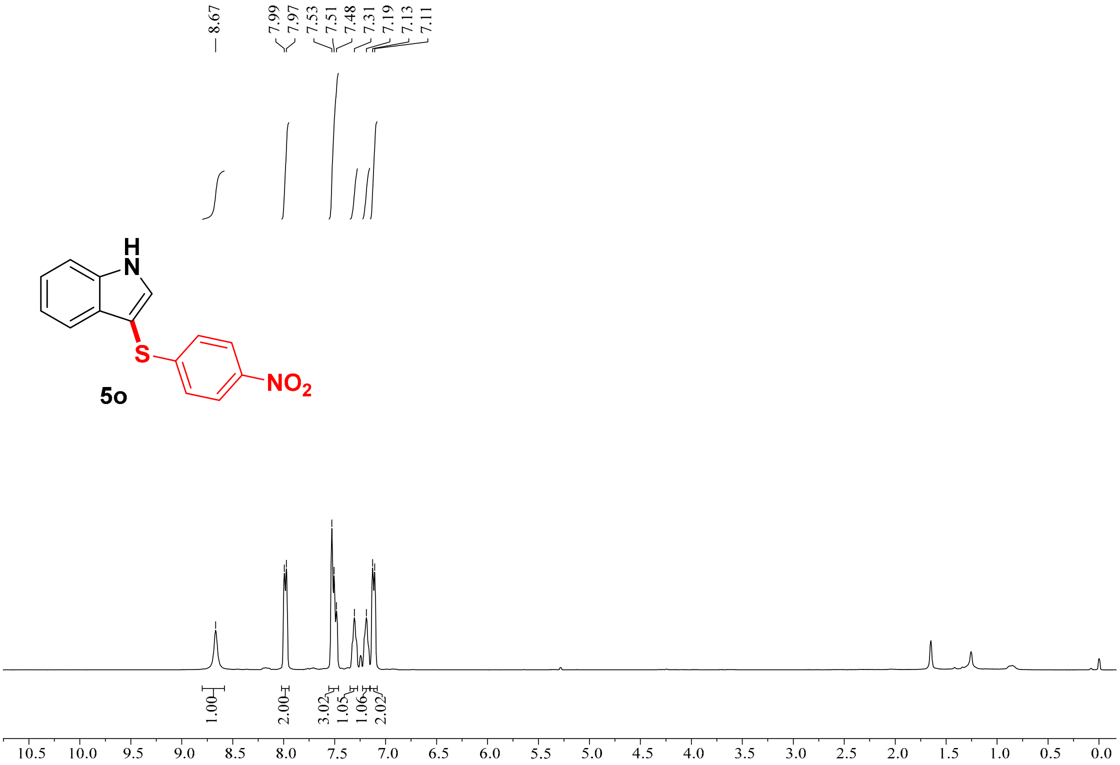
8.67
7.99
7.97
7.53
7.51
7.48
7.31
7.19
7.13
7.11
5o
NO_2
1.00
2.00
3.02
1.05
1.06
2.02
10.5 10.0 9.5 9.0 8.5 8.0 7.5 7.0 6.5 6.0 5.5 5.0 4.5 4.0 3.5 3.0 2.5 2.0 1.5 1.0 0.5 0.0

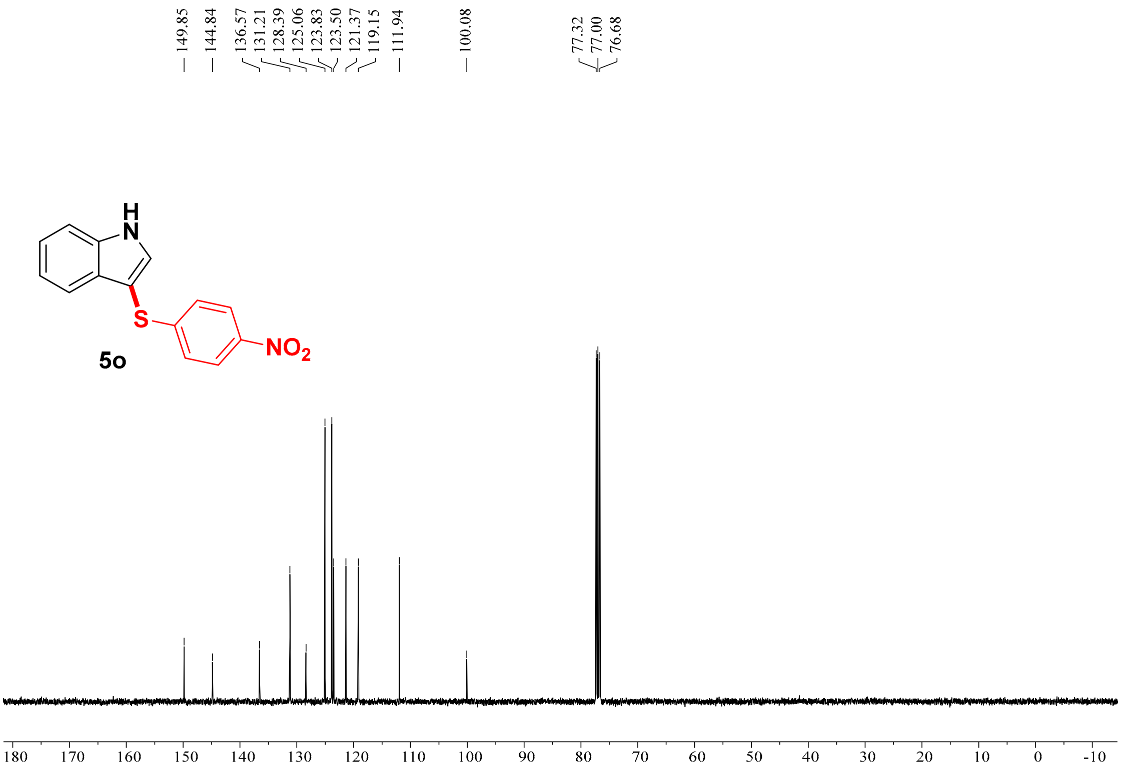
149.85
144.84
136.57
131.21
128.39
125.06
123.83
123.50
121.37
119.15
111.94
100.08
77.32
77.00
76.68
5o
NO_2
180 170 160 150 140 130 120 110 100 90 80 70 60 50 40 30 20 10 0 -10

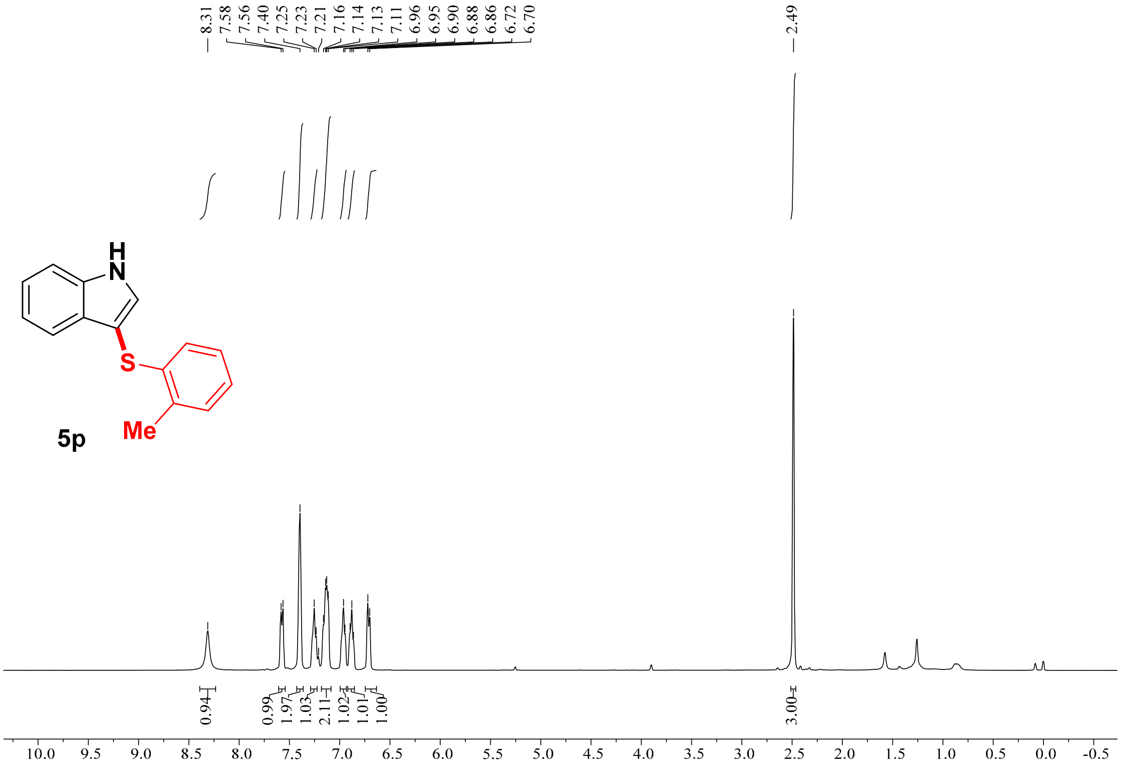
8.31
7.58
7.56
7.40
7.25
7.23
7.21
7.16
7.14
7.13
7.11
6.96
6.95
6.90
6.88
6.86
6.72
6.70
2.49
H
N
S
Me
5p
0.94
0.99
1.97
1.03
2.11
1.02
1.01
1.00
3.00
10.0 9.5 9.0 8.5 8.0 7.5 7.0 6.5 6.0 5.5 5.0 4.5 4.0 3.5 3.0 2.5 2.0 1.5 1.0 0.5 0.0 -0.5

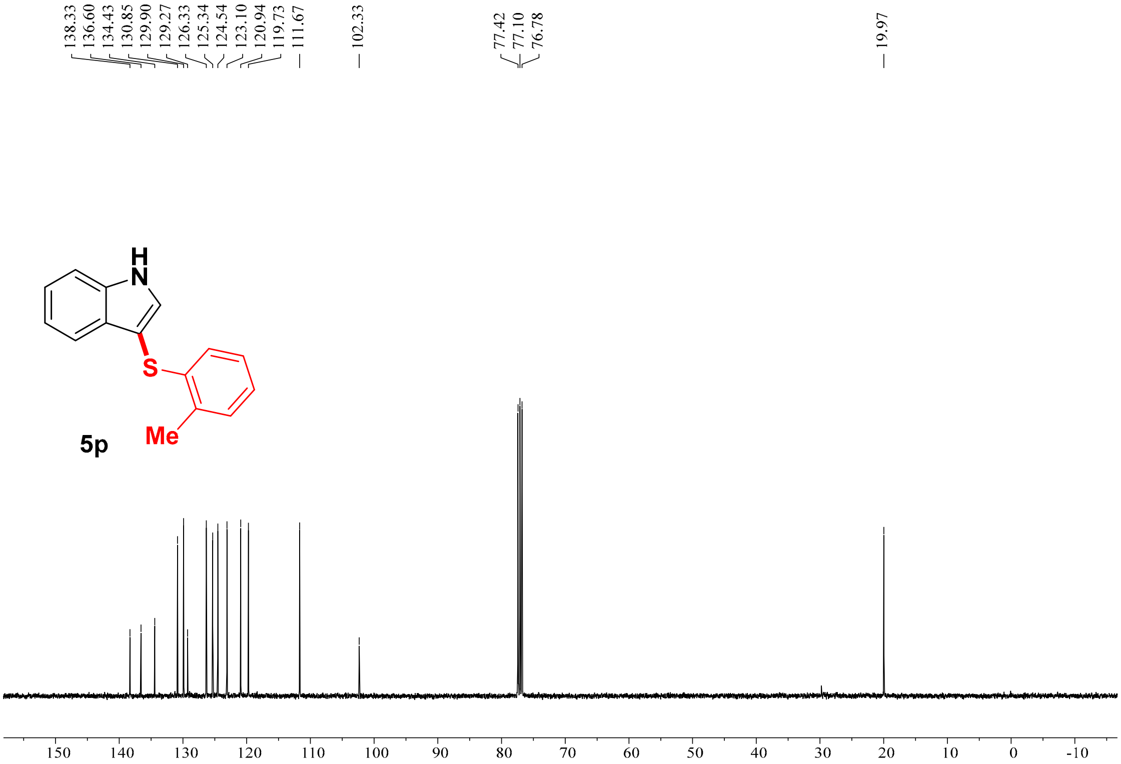
138.33
136.60
134.43
130.85
129.90
129.27
126.33
125.34
124.54
123.10
120.94
119.73
111.67
102.33
77.42
77.10
76.78
19.97
H
N
S
Me
5p
150 140 130 120 110 100 90 80 70 60 50 40 30 20 10 0 -10

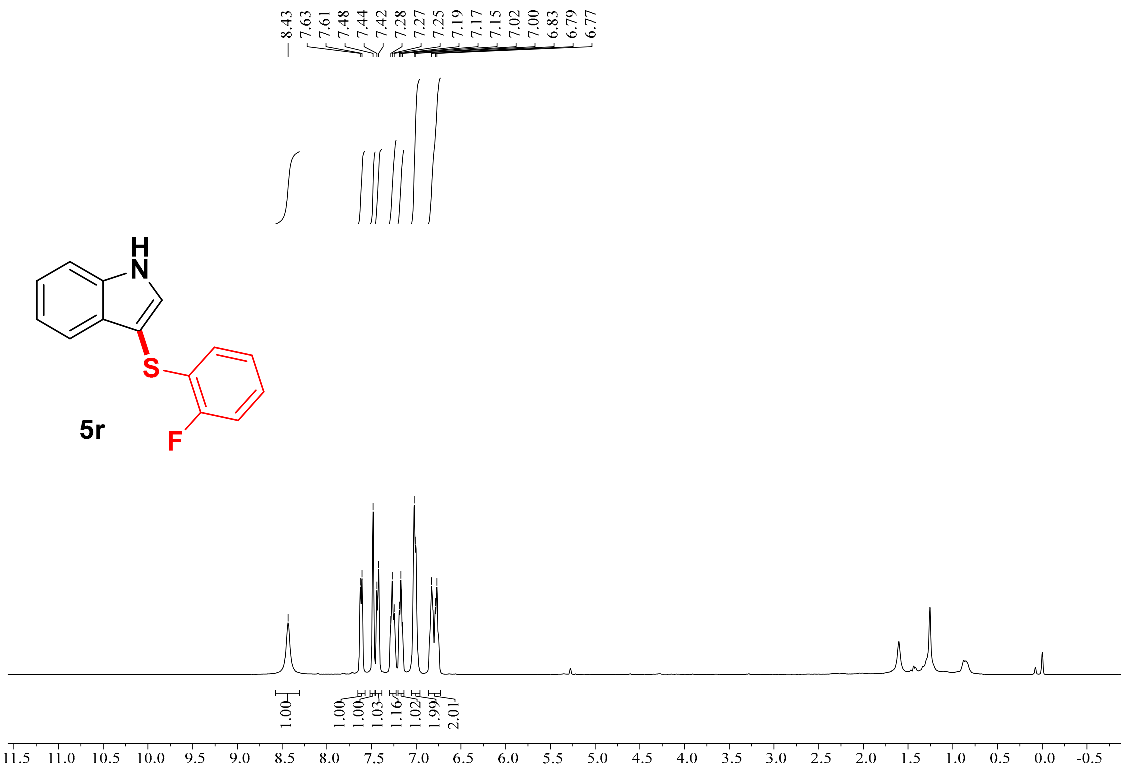

8.43
7.63
7.61
7.48
7.44
7.42
7.28
7.27
7.25
7.19
7.17
7.15
7.02
7.00
6.83
6.79
6.77
5r
1.00
1.00
1.00
1.03
1.16
1.02
1.99
2.01
11.5 11.0 10.5 10.0 9.5 9.0 8.5 8.0 7.5 7.0 6.5 6.0 5.5 5.0 4.5 4.0 3.5 3.0 2.5 2.0 1.5 1.0 0.5 0.0 -0.5

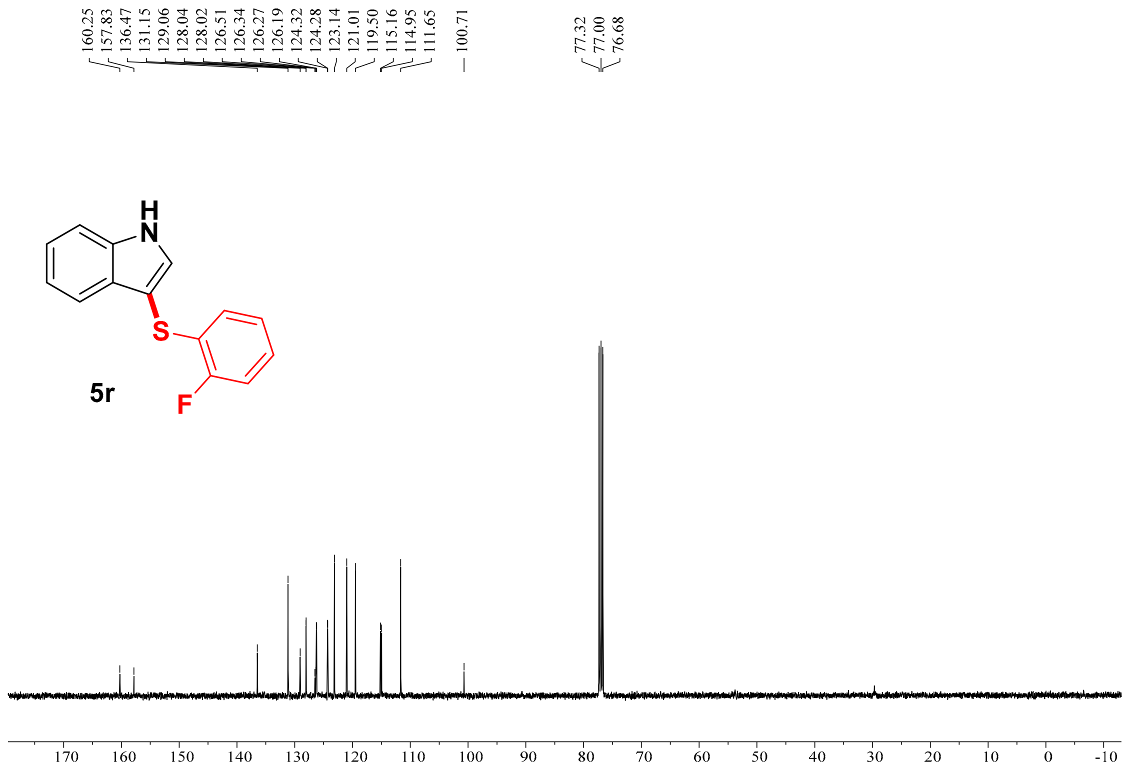

160.25
157.83
136.47
131.15
129.06
128.04
128.02
126.51
126.34
126.27
126.19
124.32
124.28
123.14
121.01
119.50
115.16
114.95
111.65
100.71
77.32
77.00
76.68
5r
170 160 150 140 130 120 110 100 90 80 70 60 50 40 30 20 10 0 -10

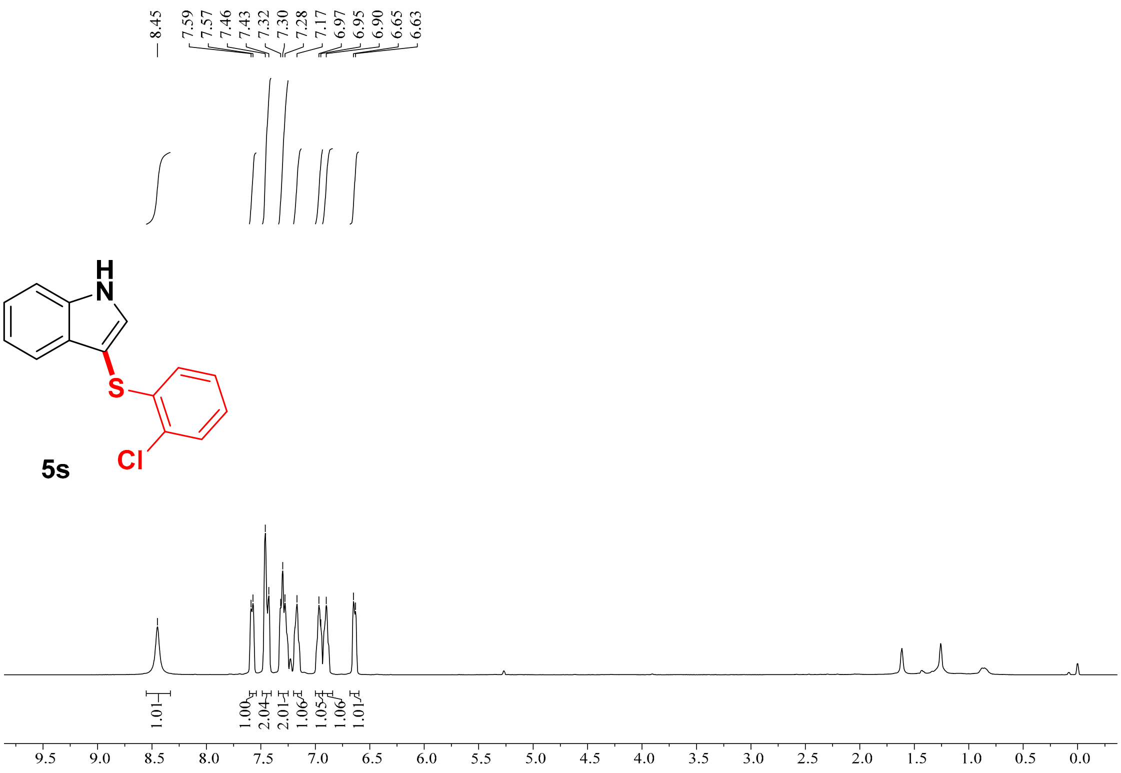
8.45
7.59
7.57
7.46
7.43
7.32
7.30
7.28
7.17
6.97
6.95
6.90
6.65
6.63
H
N
S
Cl
5s
1.01
1.00
2.04
2.01
1.06
1.05
1.06
1.01
9.5 9.0 8.5 8.0 7.5 7.0 6.5 6.0 5.5 5.0 4.5 4.0 3.5 3.0 2.5 2.0 1.5 1.0 0.5 0.0

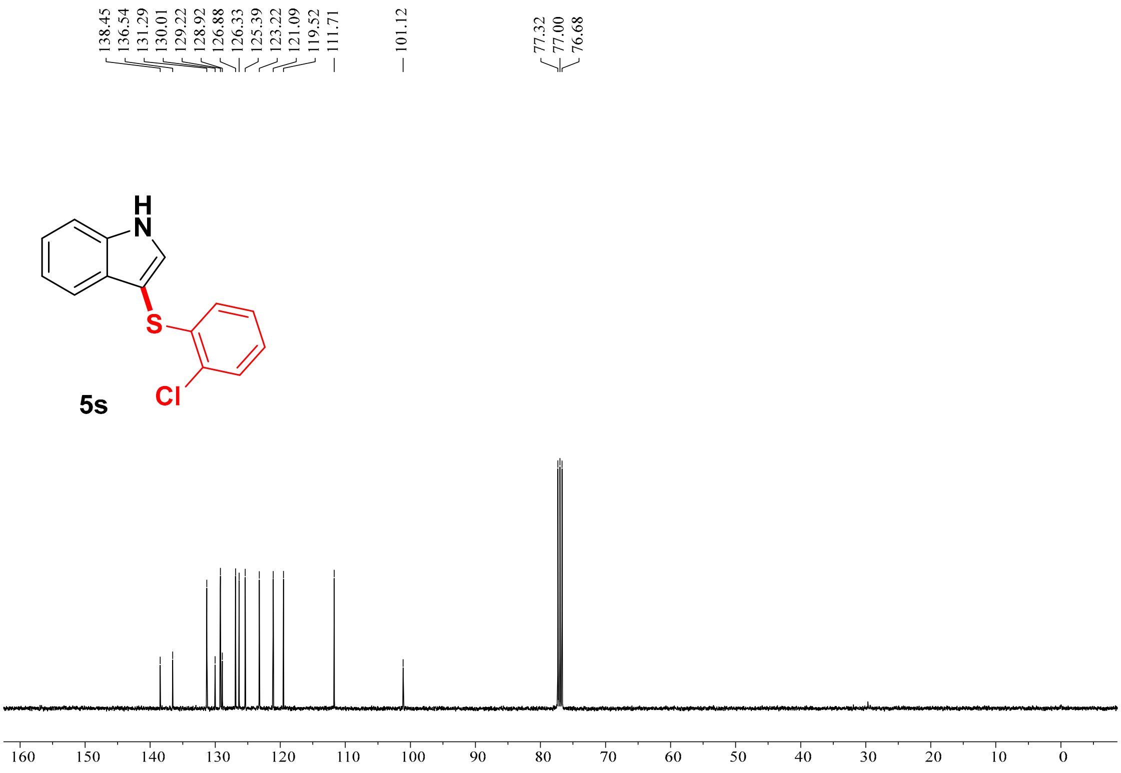
138.45
136.54
131.29
130.01
129.22
128.92
126.88
126.33
125.39
123.22
121.09
119.52
111.71
101.12
77.32
77.00
76.68
H
N
S
Cl
5s
160 150 140 130 120 110 100 90 80 70 60 50 40 30 20 10 0

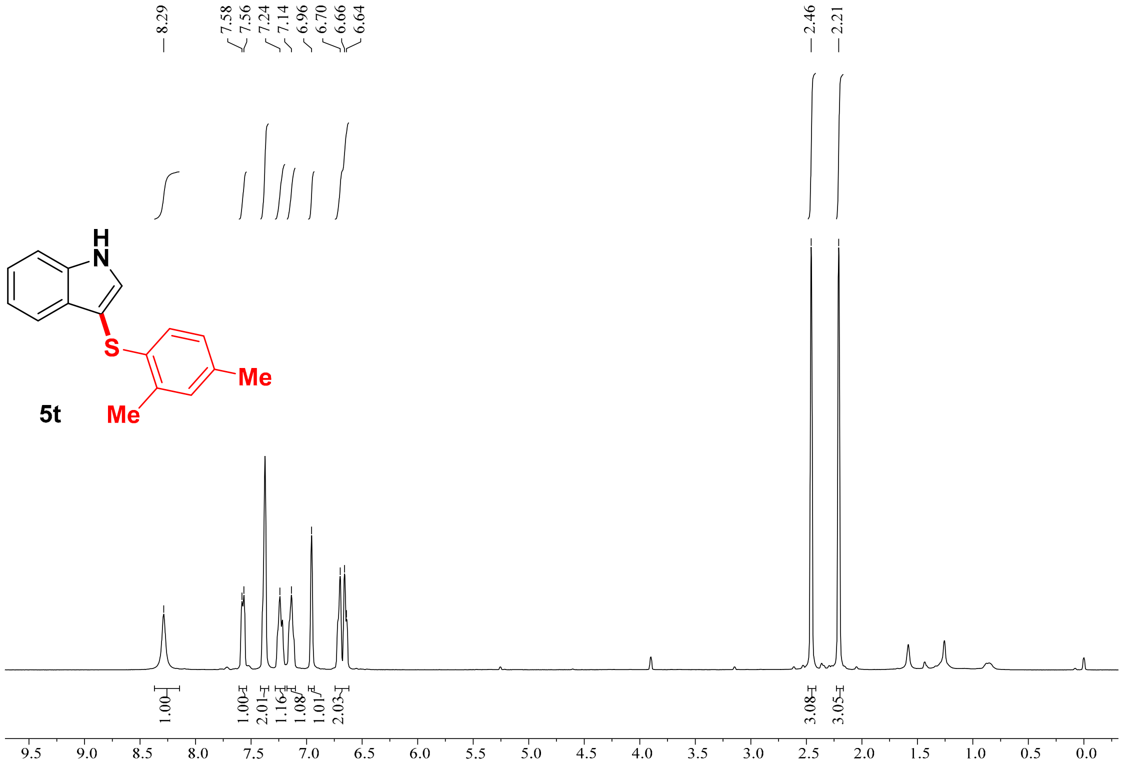
8.29
7.58
7.56
7.24
7.14
6.96
6.70
6.66
6.64
2.46
2.21
H
N
S
Me
Me
5t
1.00
1.00
2.01
1.16
1.08
1.01
2.03
3.08
3.05
9.5 9.0 8.5 8.0 7.5 7.0 6.5 6.0 5.5 5.0 4.5 4.0 3.5 3.0 2.5 2.0 1.5 1.0 0.5 0.0

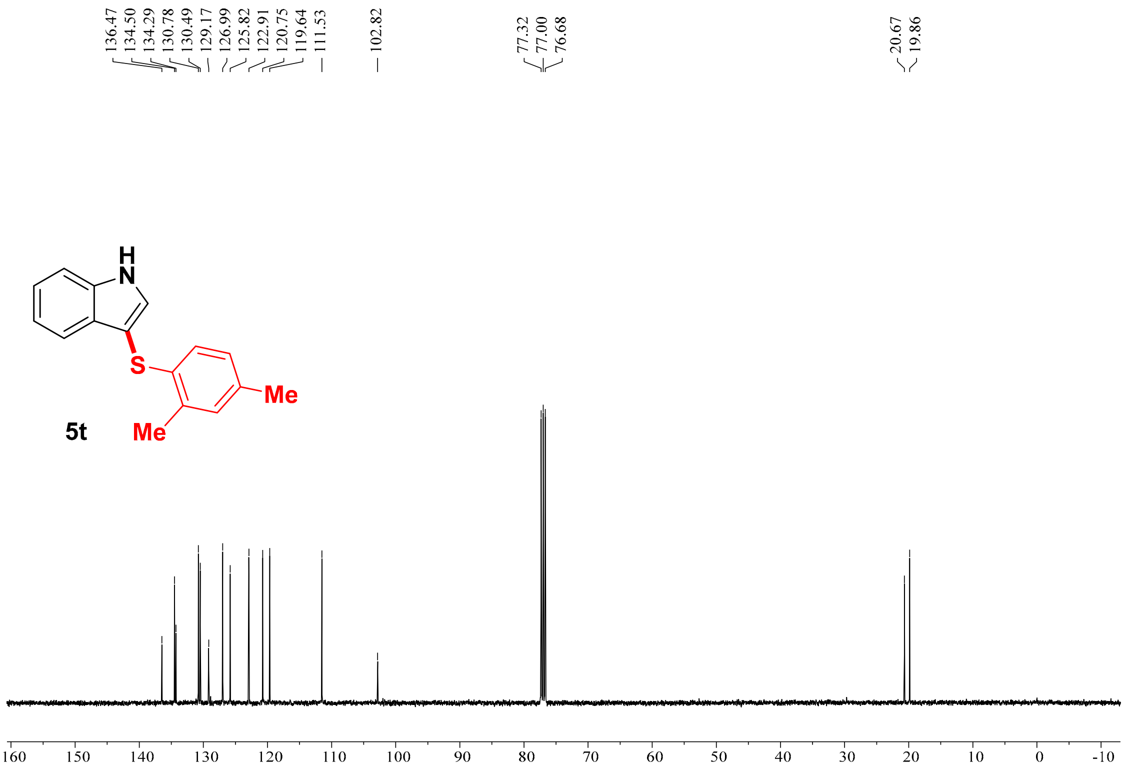
136.47
134.50
134.29
130.78
130.49
129.17
126.99
125.82
122.91
120.75
119.64
111.53
102.82
77.32
77.00
76.68
20.67
19.86
H
N
S
Me
Me
5t
160 150 140 130 120 110 100 90 80 70 60 50 40 30 20 10 0 -10
